# Label-Free Quantitative Proteomic Analysis Reveals Inflammatory Pattern Associated with Obesity and Periodontitis in Pregnant Women

**DOI:** 10.3390/metabo12111091

**Published:** 2022-11-10

**Authors:** Gerson Aparecido Foratori-Junior, Talita Mendes Oliveira Ventura, Larissa Tercilia Grizzo, Guy Howard Carpenter, Marília Afonso Rabelo Buzalaf, Silvia Helena de Carvalho Sales-Peres

**Affiliations:** 1 Department of Pediatric Dentistry, Orthodontics and Public Health, Bauru School of Dentistry, University of São Paulo, Bauru 17012-901, Brazil; 2 Centre for Host-Microbiome Interactions, Faculty of Dental, Oral & Craniofacial Sciences, Guy’s Campus, King’s College London, London SE1 1UL, UK; 3 Department of Biological Sciences, Bauru School of Dentistry, University of São Paulo, Bauru 17012-901, Brazil

**Keywords:** obesity, periodontitis, pregnancy, proteomics, saliva

## Abstract

Obesity and pregnancy may have synergistic effects on periodontal condition, and proteomics could be an ideal approach to highlight the pathophysiological mechanisms associated with these outcomes. This study analyzed the salivary proteomics related to obesity and periodontitis in women during pregnancy (T1) and after delivery (T2). Initially, 126 women were recruited and forty were allocated into groups: with obesity and periodontitis (OP); with obesity, but without periodontitis (OWP); with normal BMI, but with periodontitis (NP); with normal BMI and without periodontitis (NWP). Whole-mouth saliva was collected in T1 and T2, and proteins were extracted and individually processed by label-free proteomics (*nLC-ESI-MS/MS*). The up-regulations of *Heat shock 70 kDa protein 1A*, *1B*, and *1-like* were related to both obesity and periodontitis, separately. *Albumin* and *Thioredoxin* were up-regulated in periodontitis cases, while *Cystatins* (mainly *S*, *SA*, *SN*) and *Lactotransferrin* were down-regulated. The high abundances of *Submaxillary gland androgen-regulated protein 3B*, *Protein S100-A8*, *Matrix metalloproteinase-9*, *Heat shock 70 kDa protein 2* and *6*, *Putative Heat shock 70 kDa protein 7*, *Heat shock 71 kDa protein*, *Haptoglobin* and *Plastin-1* were significant in the combination of obesity and periodontitis. Obesity and periodontitis remarkably altered the proteome of the saliva during pregnancy with substantial alterations after delivery.

## 1. Introduction

High levels of progesterone and estrogen during pregnancy are associated with increased oral inflammation, as they reduce the patients’ immunity and exacerbate the inflammatory response in the presence of bacterial dental plaque [1]. Bacteremia related to periodontitis in pregnancy triggers an acute hepatic response phase, resulting in the production of cytokines, *prostaglandins* (PGE 2) and *interleukins* (IL-6 and IL-8), which can affect the health of the pregnant woman and the baby [1].

The same mechanism may be observed in individuals with obesity since their adipose tissue secretes inflammatory cytokines and adipokines, such as *Tumor Necrosis Factor alpha* (TNF-α), IL-6, *adiponectin*, *leptin*, *adipocytokine*, and cytoplasmic enzymes [2], which reduce the host’s immune response and cause a generalized inflammatory state of the body, including the periodontium [3].

Considering the strong potential to aid in the diagnosis of systemic diseases and its easy access for being a non-invasive method, the analysis of proteins found in saliva is an important field of scientific research. It is possible to find more than 2000 proteins and peptides in saliva that are involved with a multitude of different biological functions in the oral cavity [4].

Studies on proteomic analysis of saliva have contributed to the rapid assessment of multiple biomarkers over the years, enlightening specific signatures associated with periodontal diseases [5,6,7,8]. Changes in the salivary proteomic profile mediated by the presence of periodontitis have previously been reported in patients with obesity [9]. *Alpha-defensins* seem to play an important role in gingival inflammation and, furthermore, they may be involved in the increased susceptibility of individuals with obesity to periodontal diseases [9]. A wide range of salivary proteins of various functions were found to be significantly reduced in individuals with chronic periodontitis, whereas *Salivary acidic proline-rich phosphoprotein*, *Submaxillary gland androgen-regulated protein*, *Histatin-1*, *Fatty acid-binding protein*, *Thioredoxin* and *Cystatin-SA* were correlated with signs of periodontal attachment loss and inflammation [7].

Despite the association between overweight and periodontitis during pregnancy being reported previously [10], as well as the metabolomic profiles from plasma and saliva of pregnant women with obesity and periodontitis [11], there are no studies in the literature that have sought to evaluate salivary proteins related to obesity and periodontitis during pregnancy. Therefore, the aim of this study was to evaluate the salivary proteomic profile related to obesity and periodontitis in women during pregnancy and after delivery.

## 2. Materials and Methods

This observational, prospective, and analytical study followed the *Strengthening the Reporting of Observational Studies in Epidemiology* (STROBE) guidelines [12] and was registered in the ReBEC (https://ensaiosclinicos.gov.br/rg/RBR-2xwgc74 (accessed on 8 October 2022)) under the protocol RBR-2xwgc74.

### 2.1. Ethical Statement

In accordance with The Code of Ethics of the World Medical Association (Declaration of Helsinki—published in 1975 and revised in 2013), this study was approved by the Internal Ethics Committee from the Bauru School of Dentistry, University of São Paulo (CAAE 06624519.3.0000.5417; protocol code 3.284.822; approved on 17 April 2019). Individuals were included after approval and the signature of the written informed consent form, and there was no identification of the subjects in this study.

### 2.2. Sample Selection

Inclusion criteria were pregnant women aged 18–40 years, who were in the 3rd trimester of pregnancy (27th–39th gestational week), with regular follow-up with the obstetrician and who had adequate cognitive function during pregnancy, without impairments that required absolute rest. Exclusion criteria were twin pregnancy, patients with neuromotor impairment, arterial hypertension during pregnancy (blood pressure ≥ 140/90 mmHg), Gestational Diabetes Mellitus (hyperglycemia: ≥92 mg/dl—fasting level; ≥180 mg/dl—after 1 h; and ≥153 mg/dl—after 2 h), malnutrition (BMI < 18.50 kg/m^2^), overweight (BMI between 25.00 kg/m^2^ and 29.99 kg/m^2^), confirmed or suspected diagnosis of SARS-CoV-2 infection, with hyposalivation (<0.25 mL/min flow rate), subjects who were using or who used at some point in pregnancy antibiotics or any medication that could interfere with periodontal status and/or salivary flow (e.g., immunosuppressive, anticonvulsant or calcium channel-blocking drugs, such as cyclosporine, phenytoin, or nifedipine, respectively), who were under orthodontic treatment or any dental treatment with another professional, participants with cavitated caries lesions, with severe dental wear, with diagnosis of stages I and IV of periodontitis, with multiple tooth loss (more than two teeth per hemiarch), with self-reported systemic disease besides obesity, ex-smokers/smokers, and users of alcohol/illicit drugs.

**Figure 1:**
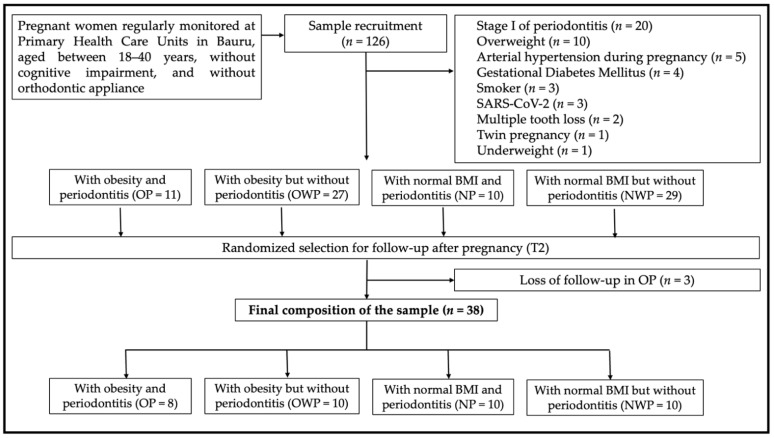
Flowchart showing sample composition.

A total of 126 women were consecutively recruited during the 3rd trimester of pregnancy from the Primary Health Care public units of Bauru, São Paulo, Brazil, between November 2020 and March 2021. A total of 49 patients were excluded from the sample and the reasons are highlighted in Figure 1, which details the selection, follow-up, and final composition of the sample (OP = 8; OWP = 10; NP = 10; NWP = 10) (Figure 1). The sample size of this study was based on previous in vivo individual salivary proteomic analysis by mass spectrometry [4,13]. The follow-up of the participants was carried out at least 1 month after delivery, between May 2021 and August 2021. Three patients in the OP group were lost to follow-up.

### 2.3. Grouping Variables

Participants were classified according to their pregestational BMI, based on the classification proposed by the World Health Organization, according to previous studies [14,15,16,17,18,19,20]. Participants with BMI ≥ 30.00 kg/m^2^ were allocated into OP and OWP groups, and those with normal BMI (18.50–25.00 kg/m^2^) were allocated into NP and NWP groups.

Regarding the periodontal classification, data collections were performed by one calibrated dentist (*kappa* = 0.95). Probing pocket depth (PPD) and clinical attachment level (CAL)/attachment loss (AL) were assessed using a standard periodontal clinical probe (Quinelato, Schobell Industrial Ltda., Rio Claro, São Paulo, Brazil). Participants were diagnosed with periodontitis as described by Tonetti and collaborators (2018) [21]. After this, according to interdental AL, periodontitis was classified in stages II and III of periodontitis [21]. Participants classified with periodontitis stages I and IV were not considered to ensure a more homogenous sample for proteomic analysis. Considering the limitations (indications and contraindications) of the gestational period, and respecting the ethical statements, the severity of periodontitis was based only on clinical parameters, and no dental radiographs were taken to avoid unnecessary exposure of pregnant women to X-rays.

### 2.4. Co-Variables

Contextual variables were related to age, educational level, and household monthly income. Education level and household monthly income categorizations were based on previous descriptions [14,15,16,17,18,19,20]. These variables were assessed to confirm that the sample was homogeneous, minimizing the bias in the proteomic analysis.

In addition to the pre-pregnancy BMI, BMIs during the 3rd trimester of pregnancy and after delivery were also obtained. Percentage of sites with bleeding on probing (BOP) [22], of dental surfaces with visible biofilm/dental plaque [23], and oral hygiene behaviors (daily toothbrushing and flossing) were registered.

### 2.5. Saliva Collection

Saliva collection was performed before periodontal examination. Patients were instructed not to consume any food or drink prior to the consultation and to properly clean their mouths before saliva collection. The consultations were carried out in the morning (09:00–11:00), considering the circadian rhythm. Patients were asked to remain seated, erect, and at rest for 15 min prior to saliva collection. First, the patients rinsed their mouths with 5 mL of deionized water before collection. After this period, unstimulated whole-mouth saliva was collected from the patients, who expelled saliva in a sterilized plastic falcon tube (50 mL) immersed on ice. After collection, saliva was centrifuged at 4500× *g* for 15 min at 4 °C in order to remove all debris. The supernatant of each sample was collected and stocked in a freezer at −80 °C until the moment of analysis [4,13].

### 2.6. Sample Preparation for Proteomic Analysis

Proteomic analysis was performed exactly as in a previous protocol [4] and all reagents were LC-MS grade and suitable for mass spectrometry. The samples (1000 µL each) were analyzed individually. Proteins were extracted using a solution (1000 µL for each sample) containing 6 M urea (≥99.9%; Sigma-Aldrich Corp. Milwaukee, WI, USA), 2 M thiourea (99.0%; Sigma-Aldrich Corp. Milwaukee, WI, USA) in 50 mM NH_4_HCO_3_, pH 7.8. Samples were three times vortexed for 10 min at 4 °C (Vortex-Genie^®^, Sigma-Aldrich Corp. Milwaukee, WI, USA), sonicated for 5 min (Cristófoli LTDA. Campo Mourão, PR, Brazil), and centrifuged at 20,817× *g* at 4 °C for 10 min (Eppendorf Ltd., Hamburg, Germany). Then, the samples were concentrated in Amicon tubes (Amicon Ultra-15 Centrifugal Filter Units—Merck Millipore^®^, Tullagreen, County Cork, Ireland) to a volume of approximately 150 μL. Proteins were reduced with 5 mM dithiothreitol for 40 min at 37 °C and alkylated with 10 mM iodoacetamide for 30 min in the dark. Samples were digested for 14 h at 37 °C by addition of 2% *trypsin* (*w*/*w*) (Thermo Scientific Pierce Trypsin Protease, Rockford, IL, USA). Digestion was stopped by the addition of 10 μL of 5% Trifluoroacetic acid and then the samples were desalted and purified using C18 Spin columns (Thermo Scientific, Rockford, IL, USA). In order to perform normalization based on total protein, an aliquot of 1 μL was taken from each sample for total protein quantification by the Bradford method (Bio-Rad, Hercules, CA, USA). The samples were then resuspended in a solution containing 3% acetonitrile and 0.1% formic acid and subjected to mass spectrometry (nanoLC-ESI-MS/MS) (Waters, Manchester, New Hampshire, UK).

### 2.7. Shotgun Label-Free Quantitative Proteomic Analysis nLC-ESI-MS/MS

Peptide identification was performed using a Xevo G2 QTof mass spectrometer coupled to a nanoACQUITY system (Waters, Manchester, New Hampshire, UK), which was operated in positive ionic nanoelectrospray mode. Data were collected using the MSE method at high energy (19–45 V) that allows data acquisition of both precursor and fragment ions in a single injection. Data acquisition scan range was 50–2000 Da. The lockspray used to ensure accuracy and reproducibility was operated with a solution of [Glu1]-fibrinopeptide (1 pmol/µL) at a flow of 0.5 µL/min.

*ProteinLynx Global Server* (PLGS) version 3.0.3 software (Waters Co., Manchester, UK) was used to process and search the continuous LC-MSE data. Proteins were identified using the software’s ion counting algorithm, and a search was performed in the *Homo sapiens* database (reviewed only, UniProtKB/Swiss-Prot) downloaded in May 2022 by UniProtKB (http://www.uniprot.org/ (accessed on 25 May 2022)). *UniProt* was used to analyze each protein by their access number. Repeated and reverse proteins, as well as fragments, were excluded. All proteins identified with a confidence level greater than 95% were included in the quantitative analysis.

### 2.8. Statistical Analysis and Bioinformatics

For clinical parameters, the Kolmogorov–Smirnov test was initially applied to verify the normality of variables. Analysis of Variance (repeated measures ANOVA) with Scheffé test as post hoc test was applied for quantitative variables with normal distribution (age, prevalence of dental plaque, and prevalence of bleeding on probing); Kruskal–Wallis with Dunn as post hoc test was applied for quantitative variables without normal distribution (maternal BMI, PPD, and CAL).

For proteomic analysis, the difference in expression between groups was expressed as *p* < 0.05 for the down-regulated proteins and 1-*p* > 0.95 for the up-regulated proteins [4]. The difference in expression between the groups was analyzed by *t* test (*p* < 0.05). The relevant comparations were performed intergroups and periods.

Protein categories were based on gene ontology (GO) annotation of the broad Biological Process, Molecular Function, Immune System, and Cell Component using ClueGo^®^ plugins of the Cytoscape^®^ 3.8.2 software. The functional distribution of proteins identified with differential expression (up- and down-regulated) in the comparison between groups and periods was performed. Terms of significance (*κ* = 0.04) and distribution were according to the percentage of the number of associated genes. The mass spectrometric proteomic data were deposited to the *ProteomeXchange Consortium* via the *PRIDE* partner repository with the data set identifier (PXD034328).

STRING^®^ database (https://string-db.org/cgi/network.pl (accessed on 29 May 2022)) was accessed for the interaction networks, establishing the interaction between up-regulated proteins identified in each group during pregnancy. False Discovery Rates (FDRs) were presented for protein interactions.

## 3. Results

Table 1 shows the systemic and periodontal parameters. Groups were homogeneous in terms of socioeconomic levels. Most individuals in all groups indicated that they had complete (total average of 50%) or incomplete (total average of 27.5%) secondary education. Regarding household monthly income, most of the sample indicated receiving up to one minimum wage (MW, approximately $240.00) (17.5%), up to two MW (35%), or up to three MW (22.5%) monthly.

Groups were also homogeneous regarding unstimulated salivary flow, showing no changes in this parameter over time as well (*p* = 0.140). The means of unstimulated salivary flow per group were 0.56, 0.55, 0.57, and 0.56 mL/min in T1 and 0.55, 0.53, 0.56, and 0.56 mL/min in T2 for OP, OWP, NP, and NWP, respectively. Most individuals from the periodontitis groups (five women from OP group and eight from NP group) were classified as stage II of periodontitis, but three pregnant women from OP and two from NP group were classified as stage III (Table 1).

**Figure 2:**
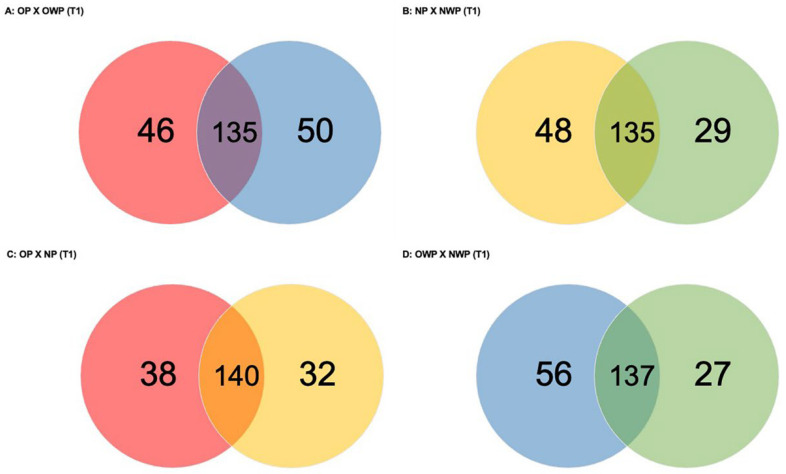
Venn diagrams showing the number of proteins identified exclusively in each group and in common between the groups for all comparations in T1.

Considering the comparison between OP and OWP in T1 (Figure 2A), a total of 231 proteins was found (46 and 50 proteins were exclusively identified in OP and OWP, respectively). Groups had 135 proteins in common: 65 were up-regulated in OP (i.e., *Protein S100-A8*, *Histatin-3*, *Submaxillary gland androgen-regulated protein 3B*—up-regulated more than 2-fold; *Matrix metalloproteinase-9*—up-regulated more than 6-fold; 5 isoforms of *Immunoglobulin*, 5 isoforms of *Alpha-amylase*, 7 isoforms of *Hemoglobin*–*Hemoglobin subunit alpha* and *beta* were up-regulated more than 10-fold; and *Statherin*—up-regulated more than 29-fold); and 38 proteins were down-regulated in OP (*Lysozyme C*, *Protein S100-A9*, *Mucin-7*, 5 isoforms of *Cystatin*, *Neutrophil defensin 1* and *3*, *Basic salivary proline-rich protein 1* and *2*—down-regulated more than 4-fold, *Lactotransferrin*—down-regulated more than 5-fold, and *WAP four-disulfide core domain protein 2*—down-regulated more than 12-fold).

In the comparison between NP and NWP in T1 (Figure 2B), a total of 212 proteins was found (48 and 29 proteins were exclusively identified in NP and NWP, respectively). Groups had 135 proteins in common: 58 were up-regulated in NP (i.e., *Cystatin-B, Submaxillary gland androgen-regulated protein 3B*, 6 isoforms of *Hemoglobin*, 10 isoforms of *Immunoglobulin*, *Albumin*, and *Serotransferrin*—up-regulated more than 2-fold, *Protein S100-A8*—up-regulated more than 3-fold, *Alpha-2-macroglobulin*—up-regulated more than 4-fold, *Protein S100-A9* and *Deleted in malignant brain tumors 1 protein*—up-regulated more than 5-fold); and 41 proteins were down-regulated in NP (i.e., *Mucin-7*, 5 isoforms of *Cystatin*, *Lactotransferrin*, 7 isoforms of *proline-rich protein*—*Salivary acidic proline-rich phosphoprotein 1/2* was down-regulated more than 5-fold, *BPI fold-containing family B member 1*—down-regulated 5-fold and *Carbonic anhydrase 6*—down-regulated 10-fold).

In the comparison between OP and NP in T1 (Figure 2C), a total of 210 proteins was found (38 and 32 were exclusively identified in OP and NP, respectively). Groups had 140 proteins in common: 73 were up-regulated in OP (i.e., *Protein S100-A8* and *A9*, *Matrix metalloproteinase-9*, 13 isoforms of *Immunoglobulin*, 8 isoforms of *proline-rich protein*, *Mucin-2*—up-regulated more than 2-fold, *Cystatin-B* and *Serotransferrin* — up-regulated more than 3-fold, 6 isoforms of *Hemoglobin*—with *Haptoglobin* and *Haptoglobin-related protein* up-regulated more than 9-fold, *Hemoglobin subunit gamma-2*—up-regulated 19-fold, *Hemoglobin subunit gamma-1* and *subunit epsilon*—up-regulated more than 20-fold, and *Hemoglobin subunit alpha*—up-regulated more than 36-fold); and 31 proteins were down-regulated in OP (i.e., *Lactotransferrin, Albumin, Neutrophil defensin 3*, *Lysozyme C*, *Carbonic anhydrase 6*, *Proline-rich protein 4*—down-regulated 3-fold, *Mucin-7*—down-regulated 4-fold, *Prolactin-inducible protein*—down-regulated 5-fold, and 4 isoforms of *Cystatin*—*Cystatin-SA* and *Cystatin-D* were down-regulated more than 3-fold and 5-fold, respectively).

In the comparison between OWP and NWP in T1 (Figure 2D), a total of 220 proteins was found (56 and 27 were exclusively identified in OWP and NWP, respectively). Groups had 137 proteins in common: 65 were up-regulated in OWP (i.e., *Albumin*, 12 isoforms of *Immunoglobulin*, *Lactotransferrin*, *Lysozyme C*, *Platin-2*, *Protein S100-A9*, *Neutrophil defensin 1* and *3*, *Cystatin-B*—up-regulated 4-fold, *Serotransferrin*—up-regulated more than 6-fold, *Complement C3*—up-regulated 7-fold, *Myeloblastin*—up-regulated 8-fold, 7 isoforms of *Hemoglobin–Hemoglobin subunit delta* was up-regulated more than 7-fold, *subunit gamma-2* was up-regulated more than 8-fold, and *subunits gamma-1* and *epsilon* were up-regulated more than 9-fold, and *WAP four-disulfide core domain protein 2*—up-regulated more than 15-fold); and 32 proteins were down-regulated in OWP (i.e., 5 isoforms of *Cystatin*, *Histatin-3*—down-regulated 2-fold, *Proline-rich protein 4*—down-regulated 4-fold, *Carbonic anhydrase 6*—down-regulated 6-fold, *Mucin-7*—down-regulated 7-fold, *Salivary acidic proline-rich phosphoprotein ½* and *BPI fold-containing family B member 1*—both down-regulated 10-fold*,* and *Statherin*—down-regulated 50-fold in OWP).

Supplementary File S1 is composed by Table S1A–D that are full tables with all the identified proteins for each group comparation (OP versus OWP; NP versus NWP; OP versus NP; and OWP versus NWP) in T1. These tables highlight the down- and up-regulated proteins, the unique proteins in each group for each comparation, and the proteins with similar expression compared to controls in each comparation. In these tables, scores (natural logs), ratio (fold change), log(e), standard deviation (SD), and *p*-values are included. Scores (natural logs) were considered to analyze the initial (prior) probability that any given protein in the database is up- or down-rated. Fold change is the ratio of A/B condition; therefore, in this study it referred to the abundance of a given protein in one group divided by the abundance of another group (its respective control group). The False Discovery Rate (FDR) value applied was 4.

**Figure 3:**
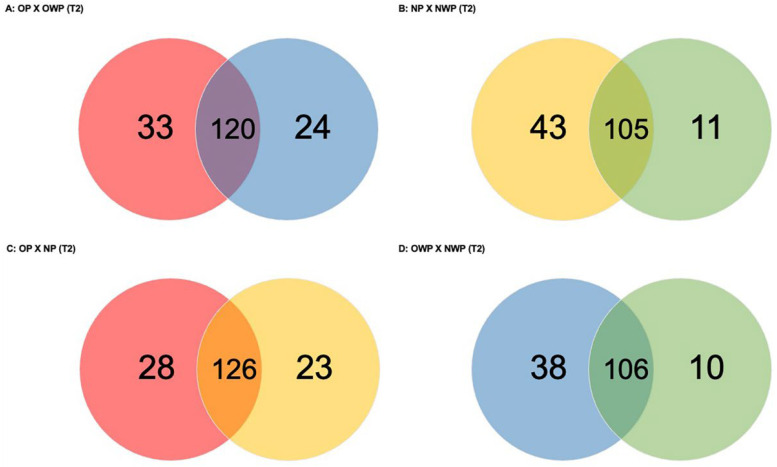
Venn diagrams showing the number of proteins identified exclusively in each group and in common between the groups for all comparations in T2.

In the comparison between OP and OWP in T2 (Figure 3A), a total of 177 proteins was found (33 and 24 were exclusively identified in OP and OWP, respectively). Groups had 120 proteins in common: 38 were up-regulated in OP (i.e., *Hemoglobin subunits delta*, *beta* and *alpha*, *Salivary acidic proline-rich phosphoprotein 1/2*, *Statherin*, and *Pyruvate kinase PKM* were up-regulated approximately 19-fold, 18-fold, 8-fold, 6-fold, 5-fold, and 4-fold, respectively); and 34 were down-regulated in OP (i.e., *Cystatin-D* and *-B*, *Hemoglobin subunits epsilon*, *gamma-1* and *-2*, *Mucin-7*, and *Protein S100-A9* were down-regulated approximately 5-fold).

In the comparison between NP and NWP in T2 (Figure 3B), a total of 159 proteins was found (43 and 11 were exclusively identified in NP and NWP, respectively). Groups had 105 proteins in common: 47 were up-regulated in NP (i.e., *Alpha-1-antitrypsin*, *Apolipoprotein A-I*, *Hemoglobin subunits delta* and *beta*, and *Neutrophil defensin-3* and *-1* were up-regulated approximately 7-fold, 6-fold, 5-fold, 4-fold, 4-fold, and 4-fold, respectively); and 33 were down-regulated in NP (i.e., *Basic salivary proline-rich protein 2*, *Basic salivary proline-rich protein 1*, and *Hemoglobin subunits gamma-1* and *-2* were down-regulated 15-fold, 9-fold, 7-fold, and 7-fold, respectively).

In the comparison between OP and NP in T2 (Figure 3C), a total of 177 proteins was found (28 and 23 were exclusively identified in OP and NP, respectively). Groups had 126 proteins in common: 42 were up-regulated in OP (i.e., *Fructose-bisphosphate aldolase A*, *6-phosphogluconate dehydrogenase, decarboxylating*, *Profilin-1*, and *Fatty acid-binding protein 5* were up-regulated approximately 3-fold); and 54 were down-regulated in OP (i.e., *LINE-1 type transposase domain-containing protein 1*, *Mucin-7*, and *Cystatin-D* were down-regulated 20-fold, 14-fold, and 10-fold, respectively).

In the comparison between OWP and NWP in T2 (Figure 3D), a total of 154 proteins was found (38 and 10 were exclusively identified in OWP and NWP, respectively). Groups had 106 proteins in common: 29 were up-regulated in OWP (i.e., *BPI fold-containing family B member 1*, *Mucin-7*, *Cystatin-B*, and *Thioredoxin* were up-regulated approximately 7-fold, 6-fold, 6-fold, and 3-fold, respectively); and 44 were down-regulated in OWP (i.e., *Histatin-3*, *Submaxillary gland androgen-regulated protein 3B*, *Putative Heat shock 70 kDa protein 7*, and *Statherin* were down-regulated 8-fold, 7-fold, 6-fold, and 5-fold, respectively).

Supplementary File S2 is composed by Table S2A–D that are full tables with all the identified proteins for each group comparation (OP versus OWP; NP versus NWP; OP versus NP; and OWP versus NWP) in T2. These tables highlight the down- and up-regulated proteins, the unique proteins in each group for each comparation, and the proteins with similar expression compared to controls in each comparation. In these tables, scores (natural logs), ratio (fold change), log(e), standard deviation (SD), and *p*-values are included.

**Figure 4:**
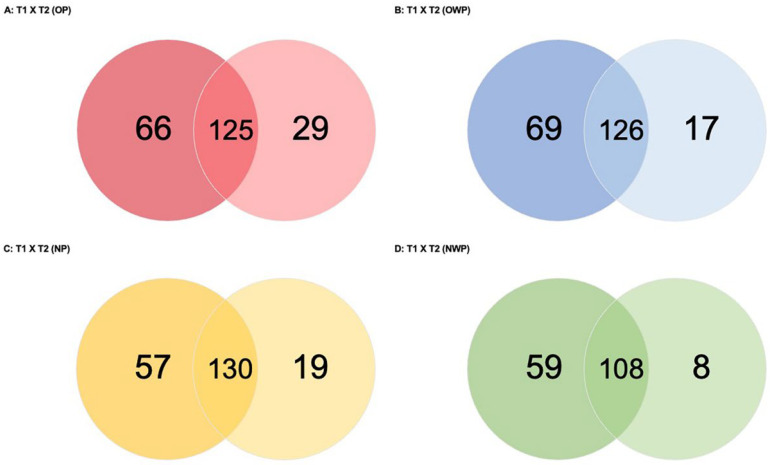
Venn diagrams showing the number of proteins identified exclusively in each period and in common between the periods for all comparations in each group.

In the comparison between periods for OP (T1 versus T2—OP; Figure 4A), a total of 220 proteins was found (66 and 29 were exclusively identified in T1 and T2, respectively). Periods had 125 proteins in common: 56 were up-regulated in T1 (i.e., *Hemoglobin subunits gamma-1*, *-2* and *subunit epsilon*, *Pregnancy zone protein*, *Protein S100-A9*, and *Haptoglobin-related protein* were up-regulated approximately 22-fold, 21-fold, 21-fold, 4-fold, 4-fold, and 4-fold, respectively); and 45 were down-regulated in T1 (i.e., *Profilin-1*, *Hemoglobin subunit delta*, *beta*, and *Fructose-bisphosphate aldolase A* were down-regulated 9-fold, 7-fold, 4-fold, and 4-fold, respectively).

In the comparison between periods for OWP (T1 versus T2—OWP; Figure 4B), a total of 212 proteins was found (69 and 17 were exclusively identified in T1 and T2, respectively). Periods had 126 proteins in common: 60 were up-regulated in T1 (i.e., *Basic salivary proline-rich protein 1*, *Lactotransferrin*; *WAP four-disulfide core domain protein 2*, and *Serotransferrin* were up-regulated approximately 29-fold, 8-fold, 6-fold, and 4-fold, respectively); and 38 were down-regulated in T1 (i.e., *Beta-2-microglobulin*, *Protein S100-A9*, and *Immunoglobulin mu heavy chain* were down-regulated 6-fold, 3-fold, and 3-fold, respectively).

In the comparison between periods for NP (T1 versus T2—NP; Figure 4C), a total of 206 proteins was found (57 and 19 were exclusively identified in T1 and T2, respectively). Periods had 130 proteins in common: 33 were up-regulated in T1 (i.e., *Protein S100-A8*, *Hemoglobin subunit epsilon*, and *Glucose-6-phosphate isomerase* were up-regulated approximately 8-fold, 7-fold, and 5-fold, respectively); and 59 were down-regulated in T1 (i.e., *Beta-2-microglobulin*, *Hemoglobin subunit zeta*, *Hemoglobin subunit alpha*, and *Haptoglobin* were down-regulated approximately 13-fold, 12-fold, 7-fold, and 5-fold, respectively).

In the comparison between periods for NWP (T1 versus T2—NWP; Figure 4D), a total of 175 proteins was found (59 and 8 were exclusively identified in T1 and T2, respectively). Periods had 108 proteins in common: 28 were up-regulated in T1 (i.e., *BPI fold-containing family B member 1*, *Statherin*, and *Lactotransferrin* were up-regulated approximately 11-fold, 4-fold, and 4-fold, respectively); and 47 were down-regulated in T1 (i.e., *Hemoglobin subunits beta*, *delta*, *epsilon*, and *gamma-2* were down-regulated approximately 20-fold).

Supplementary File S3 is composed by Table S3A–D that are full tables with all the identified proteins for the comparations between T1 and T2 for each group (G1—T1 versus T2; G2—T1 versus T2; G3—T1 versus T2; and G4—T1 versus T2). These tables highlight the down- and up-regulated proteins, the unique proteins in each period for each comparation, and the proteins with similar expression compared to controls in each comparation. In these tables, scores (natural logs), ratio (fold change), log(e), standard deviation (SD), and *p*-values are included.

The detailed functional analysis with the most significant terms according to the Biological Process, Immune System, Cellular Component, and Molecular Function by Gene Ontologies for group comparations in T1 can be found in Supplementary Figure S1. In short, for the Biological Process, the categories with the highest percentages of genes among OP and OWP in T1 were humoral immune response (25.7%), negative regulation of endopeptidase activity (17.1%), and antibacterial humoral response (9.5%); while for the Immune System, they were antimicrobial humoral response (37.5%), humoral immune response mediated by circulating immunoglobulin (32.5%), and antimicrobial humoral immune response mediated by antimicrobial peptide (Supplementary Figure S1A). Among NP and NWP in T1 for the Biological Process, they were humoral immune response (25.7%), retina homeostasis (20.8%), negative regulation of endopeptidase activity (13.9%), and antioxidant activity (10.9%), while for the Immune System, they were humoral immune response mediated by circulating immunoglobulin (44.1%), antimicrobial humoral response (35.3%), and mucosal immune response (8.8%) (Supplementary Figure S1B). When OP was compared to NP in T1, the highest percentages of genes for the Biological Process were related to the defense response to bacterium (22.4%) and tissue homeostasis (21.4%), while for the Immune System they were antimicrobial humoral response (44.4%) and complement activation (37%) (Supplementary Figure S1C).

In the same way, the detailed functional analysis with the most significant terms according to the Biological Process, Immune System, Cellular Component, and Molecular Function by Gene Ontologies for group comparations in T2 can be found in Supplementary Figure S2. In short, for the Biological Process, the categories with the highest percentages of genes among OP and OWP in T2 were retina homeostasis (24.6%), antioxidant activity (15.4%), antimicrobial humoral response (12.3%), and cysteine-type endopeptidase inhibitor activity (12.3%), while for Immune System, they were antimicrobial humoral response (40%) and complement activation, classical pathway (35%) (Supplementary Figure S2A). Among NP and NWP in T2, for the Biological Process, the categories with the highest percentages were defense response to bacterium (26.1%), humoral immune response (23.9%), and retina homeostasis (18.5%), while for Immune System, they were humoral immune response mediated by circulating immunoglobulin (62.5%) and antimicrobial humoral response (37.5%) (Supplementary Figure S2B). When OP was compared to NP in T2, the highest percentages of genes for the Biological Process were related to the defense response to bacterium (22.5%), humoral immune response (20%), and retina homeostasis (15.8%), while for the Immune System, they were humoral immune response mediated by circulating immunoglobulin (46.7%) and antimicrobial humoral response (40%) (Supplementary Figure S2C).

Finally, the detailed functional analysis with the most significant terms according to the Biological Process, Immune System, Cellular Component, and Molecular Function by Gene Ontologies for comparations between periods can be found in Supplementary Figure S6. In short, when the Biological Process by GO was compared between T1 and T2 for OP, the main terms were defense response to bacterium (20.3%), humoral immune response (19.6%), and retina homeostasis (15.2%) (Supplementary Figure S6A), while for the Immune System, they were humoral immune response mediated by circulating immunoglobulin (45.7%) and antimicrobial humoral response (37.1%). For the Biological Process compared between periods for NP, the following percentages for the same main terms were found: defense response to bacterium (21.1%), humoral immune response (19.5%), and retina homeostasis (14.6%), while for the Immune System, they were humoral immune response mediated by circulating immunoglobulin (57.7%) and antibacterial humoral response (26.9%) (Supplementary Figure S3C).

**Figure 5:**
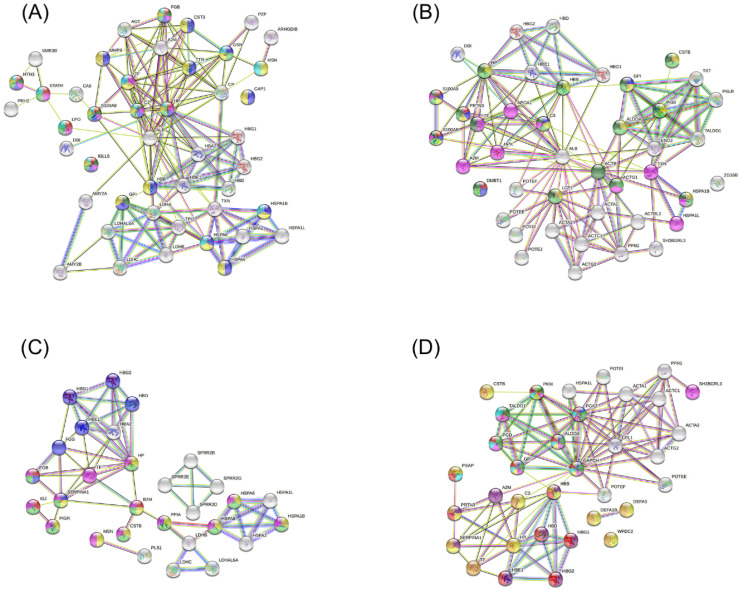
Interaction networks between up-regulated proteins identified in each group during pregnancy. (**A**) OP versus OWP comparation. Proteins marked according to some biological processes followed by the False Discovery Rate (FDR) value: red (defense response to bacterium, FDR = 0.001), dark green (humoral immune response, FDR = 0.009), pink (response to bacterium, FDR = 0.003), dark blue (immune response, FDR < 0.001), light green (defense response to other organism, FDR = 0.033), yellow (immune system process, FDR = 0.001), and light blue (interspecies interactions between organisms, FDR = 0.049). (**B**) NP versus NWP comparation. Proteins marked according to some biological processes: red (antimicrobial humoral response, FDR = 0.044), dark blue (humoral immune response, FDR = 0.005), yellow (leukocyte activation involved in immune response), light green (immune response, FDR = 0.001), pink (regulation of response to stress, FDR = 0.030), and dark green (immune system process, FDR = 0.009). (**C**) OP versus NP comparation. Proteins marked according to some biological processes: red (antibacterial humoral response, FDR = 0.029), dark blue (blood coagulation, FDR < 0.001), yellow (leukocyte activation, FDR < 0.001), dark green (immune response, FDR = 0.006), and pink (immune system process, FDR = 0.016). (**D**) OWP versus NWP comparation. Proteins marked according to some biological processes: dark blue (gluconeogenesis, FDR < 0.001), dark green (glucose metabolic process, FDR < 0.001), purple (blood coagulation), yellow (humoral immune response, FDR < 0.001), pink (generation of precursor metabolites and energy, FDR < 0.001), light blue (carbohydrate derivative metabolic process, FDR = 0.032), red (catabolic process, FDR = 0.004), orange (immune system process, FDR = 0.001).

Figure 5 shows the interaction networks between up-regulated proteins identified in each group during pregnancy for the following comparations: OP versus OWP (Figure 5A), NP versus NWP (Figure 5B), OP versus NP (Figure 5C), and OWP versus NWP (Figure 5D). Regarding the comparations between OP and OWP, the proteins that had the greatest number of functions were *Fibrinogen beta chain* (FGB), *Histatin-3* (HTN3), *S100-A8* (S100A8), *Complement C3* (C3), *Haptoglobin-related protein* (HP), and *Immunoglobulin lambda like polypeptide 5* (IGLL5) (Figure 5A). For the comparations between NP and NWP, they were *S100-A9* (S100A9), *S100-A8* (S100A8), *Myeloblastin* (PRTN3), and *Complement C3* (C3) (Figure 5B). For the comparation between OP and NP, the proteins with the greatest number of functions were *Beta-2-microglobulin* (B2M), *Alpha-1-antitrypsin* (SERPINA1), and *Fibrinogen beta chain* (FGB) (Figure 5C). For the comparations between OWP and NWP, they were *Pyruvate kinase* (PKM), *Phosphoglycerate kinase 2* (PGK2), *6-phosphogluconate dehydrogenase, Decarboxylating* (PGD), *Fructose-bisphosphate aldolase A* (ALDOA), *Glucose-6-phosphate isomerase* (GPI), and *Glyceraldehyde-3-phosphate dehydrogenase* (GAPDH) (Figure 5D). The main functions of these proteins can be found in the legend of Figure 5.

## 4. Discussion

With technological advances, many studies sought to identify disease biomarkers through body fluids. The analysis of saliva biomarkers has been widely performed because it is an easy, non-invasive, and, consequently, painless method. Before the detection of salivary biomarkers, the mapping of differentially expressed proteins in the saliva of the target population is necessary. Our results highlighted several differentially expressed proteins associated with obesity and periodontitis separately. Nonetheless, this study calls attention to the importance of those up- or down-regulated proteins when obesity and periodontitis are present in combination during pregnancy, such as *Submaxillary gland androgen-regulated protein 3B, Protein S100-A8*, *Matrix metalloproteinase-9* (MMP9), *Heat shock 70 kDa protein 2* and *6*, Putative *Heat shock 70 kDa protein 7*, *Heat shock 71 kDa protein*, *Haptoglobin*, *Plastin-1, Prolactin-inducible protein*, and *Alpha-defensins 1* and *3*.

An important and recent report on the longitudinal changes in salivary proteomics across term pregnancy showed that most differentially regulated proteins that they found were associated with neutrophil degranulation, the regulation of Toll-like receptor by endogenous ligands, antimicrobial peptide function, platelet function regulation, and glucose metabolism [24]. Among the proteins related to these mechanisms they cited *Heat shock protein cognate 71 kDa*, *Protein S100-A8, Protein S100-A9, Cathepsin-D*, MMP9, *Fructose-bisphosphate aldolase A, Complement C3, Lactotransferrin, Myeloblastin, Enolase-1*, and *Galectin-3-binding protein* [24].

Higher abundances of MMP9, *S100 proteins* (*S100-A6*/*-A8*/*-A9*), *Complement C3*, *profilin-1*, *Alpha-2-macroglobulin*, *Haptoglobin*, *Submaxillary gland androgen-regulated protein*, *Histatin-1*, *Fatty acid-binding protein*, *Thioredoxin*, and *Albumin* were previously linked to periodontitis [6,7,9,25,26,27,28,29,30]. In contrast, lower levels of *Lactotransferrin*, *Prolactin-inducible proteins*, *Salivary acid proline-rich phosphoprotein 1/2*, and *Cystatin* (mainly *S*, *SA*, and *SN*) were shown to be associated with periodontitis. Our findings are in line with these previous studies and the main highlights are discussed in detail below.

In this study, pregnant women with periodontitis (OP and NP groups) had higher levels of *Albumin* when compared to their respective controls (OWP and NWP groups, respectively). We hypothesized *Albumin* levels were influenced by gestational hormones since all groups showed an increase in albumin levels after delivery. The main function of *Albumin* is the regulation of the colloidal osmotic pressure of blood and some hormones, acting as an ion transporter as well [7]. It is believed that some periodontal microbes that trigger an inflammatory response, such as *T. denticola*, increase the levels of salivary *Albumin*. Also, these microbes use *Albumin*, as well as *Immunoglobulins,* as potential energy sources [31].

*Submaxillary gland androgen-regulated protein 3B* is a secreted endopeptidase that inhibits the cleavage of peptide bonds of non-terminal amino acids and has a role in the regulation of sensory perception of pain [7]. Yet, previous evidence found that lipopolysaccharide of *P. gingivalis* binds to *Submaxillary gland androgen-regulated protein 3B* [32], and probably it is related in promoting angiogenesis and establishing microvasculature, associating with periodontal diseases. In this study, OP and NP also had higher levels of *Submaxillary gland androgen-regulated protein 3B* when compared to OWP and NWP, but the level was more than 2-fold higher in OP when compared to NP. Curiously, women with obesity (OP and OWP) showed a decrease in this protein after delivery, while eutrophic women (NP and NWP) had an increase. *Thioredoxin* is related to cellular oxidant detoxification, cellular responses to a toxic substance, cell redox homeostasis, and the inhibition of caspase-3 activity that interferes with cellular apoptosis [7]. In this study, *Thioredoxin* was approximately 3-fold and 2-fold up-regulated in OP and NP, respectively, when compared to their controls (OWP and NWP), but with no difference for the comparation of OP versus NP.

In this study, *Protein S100-A8* was also up-regulated in the saliva of pregnant women with obesity and periodontitis, and after delivery the level of this protein increased even more in this group. *Protein S100-A8* and *S100-A9* were up-regulated approximately 4-fold and 5-fold, respectively, in NP when compared to NWP. These proteins are calcium- and zinc-binding proteins with functions involving proinflammatory, antimicrobial, oxidant-scavenging, and apoptosis-inducing activities. Their proinflammatory activity includes the recruitment of leukocytes, promotion of cytokine and chemokine production, and regulation of leukocyte adhesion and migration [24,33]. Previous evidence pointed out that these proteins are involved in neutrophil migration to inflammatory sites [34], corroborating our findings. Nonetheless, our results suggest their up-expression was more significative in OP than in NP, therefore being possible biomarkers of the combination of obesity and periodontitis during pregnancy, and this should be evaluated in future studies.

MMP9 was separately associated with both obesity and periodontitis in this study, but it was even more expressed in the saliva of pregnant women with the combination of these outcomes, as a result of those two distinct inflammatory processes. In contrast, it was not present in pregnant women with normal BMI and without periodontitis. MMP9 is an enzyme known to degrade many components of the extracellular matrix, having a role both in the physiological tissue remodeling and in the pathological tissue destruction [33]. Previous studies highlighted that MMP9 is one of the major collagen-degrading enzymes in saliva [33,35], which is associated with periodontitis. Interestingly, our report shows that pregnant women with obesity and periodontitis expressed higher levels of *Histatin-3* and *Metalloproteinase inhibitor 1* (MMP1), with the latter being a protein uniquely expressed in OP. *Histatin-3* is a salivary protein that exhibits antibacterial and antifungal activities, and it has the His3-(20–43)-peptide, which is a potent inhibitor of MMP2 and MMP9 [36], similarly to MMP1. Therefore, we hypothesized that this is a physiological defense mechanism that occurs in OP to prevent pathological tissue destruction upon the high levels of MMP9.

An important result from this study that deserves attention is related to the expression of *Heat shock proteins* (HSPs). In addition to being related to the immune response and interspecies interactions between organisms (Figure 5), HSPs also actively participate in the metabolic and catabolic processes of the organism. HSP70 and HSP71 are examples of different classes of this protein. HSP70 has intracellular and extracellular activities, including cytoprotection and immune modulation response. Due to its protective role and inhibition of apoptosis, HSP70 protects cells from tissue destruction [37]. Previous evidence showed a positive expression of HSPs in the basal layer of periodontal pockets, highlighting that there is an increase in the infiltration of mononuclear inflammatory cells below the basal layer of periodontal pockets [37,38,39]. Therefore, periodontal bacteria stimulate the periodontal cells to increase the expression of HSPs, which, in turn, stimulate macrophages and other inflammatory cells to produce proinflammatory cytokines, a mechanism that is involved in the tissue destruction of periodontitis [37,38,39].

In this study, *Heat shock 70 kDa protein 1A*, *1B* and *1-like* were more expressed in OP and NP when compared to their respective controls (OWP and NWP, respectively). However, when comparing OWP with NWP, these proteins were also more expressed in the former, showing their relationship with the inflammatory process resulting from obesity as well. Nevertheless, our findings call for attention to be paid to the greater expression of *Heat shock 70 kDa protein 2* and *6*, *Putative Heat shock 70 kDa protein 7*, and *Heat shock 71 kDa protein*, which seem to be proteins that mark the combination of obesity and periodontitis during pregnancy. Interestingly, *Heat shock 70 kDa protein 6* and *Putative Heat shock 70 kDa protein 7* were not found in the saliva samples of women from the OP group after delivery. Similarly, *Heat shock 70 kDa protein 6*, *Putative Heat shock 70 kDa protein 7*, *Heat shock 70 kDa protein 2*, and *Heat shock cognate 71 kDa protein* were present in the saliva samples of the NP group in T1 only. We suggest that future studies should be carried out to establish a deeper understanding of HSPs as potential biomarkers of periodontitis in pregnancy, being associated, or not, with obesity.

A classical study had already found higher levels of *Haptoglobin* in periodontitis cases [40]. That result was confirmed by Haigh and collaborators (2010), who indicated that this protein was associated with host defense [27]. Similarly, our results showed that *Haptoglobin* was an important up-regulated protein in the saliva of pregnant women with obesity and periodontitis. Despite this protein being more abundant in the saliva of NP in comparation to NWP, the level of this protein was even higher in individuals with both obesity and periodontitis. *Haptoglobin* is a hemoglobin-binding acute-phase protein that possesses anti-inflammatory and antioxidative properties. This protein may act as a bacteriostatic agent and, indirectly, as an antioxidant due to its facility to bind free hemoglobin and promote its clearance by macrophages. Considering that Langerhans cells in the epithelium can also synthesize *Haptoglobin,* its levels may be in increased expression in local or systemic inflammation [41], justifying our results related to the higher levels of this protein in obese women with periodontitis.

A recent study showed an underrepresentation of cysteine endopeptidase inhibitor activity in healthy pregnant women and pregnant women with gingivitis (mediated by *type-2 cystatins*, mainly *Cystatin-S*, *-SA*, and *-SN*) [42]. Previous evidence also showed lower levels of the *S-type salivary cystatins* during gingivitis and periodontitis [43,44]. These results are in line with our study. When compared to OWP, pregnant women with obesity and periodontitis showed a down-regulation of *Cystatin-S*, *-SA*, *-SN*, *-B*, and *-D*, while *Cystatin-C* was up-regulated. When NP was compared to NWP, *Cystatin-S*, *-SA*, *-SN*, *-C*, and *-D* were down-regulated, and *Cystatin-B* was up-regulated. We hypothesize that as *Cystatin-C* is related to hormonal influence, endotheliosis, and glomerular filtration rate [45], its higher expression was expected in OP due to the physiological mechanisms related to obesity and pregnancy.

*Cystatins* are protease inhibitors abundantly found in saliva and they have an important role in inhibiting tissue-destructive proteases in inflammatory processes, such as lysosomal cathepsins B, H, and L in the oral cavity [42]. Additionally, despite *cystatins* not being able to inactivate the proteases from bacterial origin, they play an important role in inhibiting the growth of species associated with periodontal impairment, such as *P. gingivalis* and *A. actinomycetemcomitans* [42], justifying our findings related to the low levels of *cystatins* in OP and NP. In addition, to reinforce this hypothesis, our results showed that other important antimicrobial proteins, such as *BPI fold-containing family A member 2* and *Lipocalin-1* (or *Putative lipocalin 1-like protein 1*) were less expressed in OP and NP, and, curiously, there was an increase in the levels of *Cystatin-C*, *-S*, *-SA*, *-SN*, and *Lipocalin-1* in OP after delivery, while *Cystatin-B* and *Cystatin-C* decreased even more. In the NP group, there was an increase in *Cystatin-SN* and *Lipocalin-1* levels after delivery, but *Cystatin-SA*, *-B*, *-C*, and *-D* decreased.

*Prolactin-inducible protein* (PIP) is responsible for the negative regulation of the T cell apoptotic process, positive regulation of gene expression, and proteolysis. PIP binds to a variety of oral bacteria, suggesting it has a role in protecting the oral mucosa by inhibiting bacterial colonization and growth [27]. Similarly, *Lactotransferrin* is an iron-binding protein, and its antibacterial effect is achieved by competing for iron with bacteria, thereby inhibiting bacterial growth [25]. In this study, both PIP and *Lactotransferrin* were down-regulated in pregnant women with periodontitis (OP and NP) when compared to OWP and NWP (*Lactotransferrin* was down-regulated 5-fold and 3-fold in OP and NP, respectively). When OP was compared to NP, PIP was more than 5-fold down-regulated in OP, showing its down-regulation is strongly associated with the combination of obesity and periodontitis during pregnancy.

Interestingly, this study showed that *Plastin-1* is a specific protein present in saliva of pregnant women with periodontitis (both OP and NP), but with higher expression when periodontitis is associated with obesity (approximately 4-fold), and this protein is present in these groups only during pregnancy. To the best of our understanding, there is no previous evidence associating *Plastin-1* with periodontitis. Baliban et al. (2012) found *Plastin-1* and *-2* in the investigated samples (health and periodontitis cases), but there was no intergroup difference, and the authors did not indicate the plausible mechanism of this protein [46]. Previous evidence showed a positive correlation of *L-Plastin* (or *Plastin-2*) with the presence and severity of periodontitis, suggesting that L-Plastin-expressing cells are participating in local inflammatory responses, and the activation of *L-Plastin* mediates leukocyte adhesion and migration, as well as osteoclast adhesion and bone resorption [47]. Nevertheless, there is a gap in the literature regarding the role of *Plastin-1*. Recently, *Plastin-1* was shown to be highly homologous to *Plastin-3*, whose mutations are responsible for X-linked osteoporosis. In addition, it was revealed that *Plastin-1* promotes osteoblast differentiation by regulating intracellular Ca^2+^ [48]. Therefore, we hypothesize that the presence of *Plastin-1* in OP and NP only in T1 could be a compensatory mechanism in those pregnant women due to the high inflammatory process and bone loss, acting in bone homeostasis. Further investigations must pay attention to the role and the mechanism of *Plastin-1* in periodontitis individuals.

In this study, proteins related to glycolytic, carbohydrate catabolic, glucose metabolic, organophosphate metabolic, and carbohydrate derivative metabolic processes (such as *6-phosphogluconate dehydrogenase, Decarboxylating*; *Pyruvate kinase PKM*; *Glyceraldehyde-3-phosphate dehydrogenase*; *Transaldolase*; *Fructose-bisphosphate aldolase A*; *Phosphoglycerate kinase 2*; and *Glucose-6-phosphate isomerase*) were more expressed in individuals with obesity (Figure 5). In contrast, proteins related to the immune system, such as those associated with the antimicrobial humoral response and response to other organism (such as *BPI fold-containing family B member 1* and *2*, *Secretory leucocyte peptidase inhibitor*, *Mucin-7*, *Histatin-3*, and *Statherin*) were down-regulated in individuals with obesity (Figure 5). The literature is scarce regarding salivary proteomic analysis in association with obesity. Lamy et al. (2015) reported higher levels of *Zinc-α-2 glycoprotein* in individuals with obesity, as well as a tendency for them to present higher levels of *Carbonic Anhydrase 6* (CA-VI) [49]. Our findings are partially in accordance with that evidence. In this study, the CA-VI levels were decreased in individuals with obesity (OP and OWP) when compared to their controls (NP and NWP, respectively), while *Zinc-α-2 glycoprotein* was positively associated with obesity when we compared OP versus NP since it was a specific protein found only in OP; however, there was no intergroup difference when OWP was compared to NWP.

Rangé et al. (2012) suggested an overexpression of *alpha-defensins* in individuals with obesity and revealed that the down-expression of the *alpha-defensins* in the saliva of periodontitis patients versus non-periodontitis patients seems to be independent of the obesity status [9]. In this study, *Neutrophil defensin 1* and *3* were down-regulated in OP, even when they were compared to OWP and NP. We did not find intergroup differences for these proteins when we compared NP to NWP. Therefore, we suggest that *Neutrophil defensin 1* and *3* are further investigated as potential biomarkers of the combination between obesity and periodontitis during pregnancy.

This study has some limitations. Future evaluations must be carried out through the gestational trimesters and with more cut-time points after delivery to understand the cause and effect regarding the physiopathology of the disease during pregnancy. Moreover, in this study, individuals with stages II and III of periodontitis were included in the same sample group (for both OP and NP). Ideally, future longitudinal studies with larger samples should analyze specific proteins expressed in saliva considering the different stages of the periodontal diseases (including gingivitis as well), and of the BMI range (i.e., underweight, and overweight classifications), to ensure a better biological understanding regarding the progression of the diseases. Moreover, this study did not evaluate the nutritional/dietary profile of the sample. It is extremely important to understand how the dietary pattern would reflect in the salivary proteomic analysis of the population studied. For this, future transdisciplinary studies, involving well-defined protocols for characterizing the dietary pattern, should be conducted. Furthermore, this study did not access the bacterial profile of the sample, but metagenomic assessments would contribute to our understanding of the identified proteins and their association with outcomes, taking into account, for instance, qualitative and quantitative bacterial evaluations of saliva and gingival fluid. Finally, future studies using specific methods that isolate and quantify the proteins presented here are necessary to validate our results (i.e., enzyme-linked immunosorbent assay). Despite the limitations, this is the first study to investigate the biological mechanisms related to salivary proteome associated with obesity and periodontitis during pregnancy and after delivery using an individual label-free quantitative shotgun proteomic analysis.

## 5. Conclusions

In conclusion, obesity and periodontitis remarkably altered the proteome of the saliva during pregnancy with specific alterations after delivery. Proteins associated with the humoral immune response and antibacterial humoral response were highly expressed in women with periodontitis, while proteins related to glycolytic, carbohydrate catabolic, glucose metabolic, organophosphate metabolic, and carbohydrate derivative metabolic processes were more expressed in individuals with obesity.

## Figures and Tables

**Table 1:** Systemic and periodontal parameters of the sample.

	OP (n = 8) Mean ± SD Median [1st–3rd Quartiles]	OWP (n = 10) Mean ± SD Median [1st–3rd Quartiles]	NP (n = 10) Mean ± SD Median [1st–3rd Quartiles]	NWP (n = 10) Mean ± SD Median [1st–3rd Quartiles]	*p*
**Age (years)**	29.10 ± 5.02	25.00 ± 5.09	26.00 ± 4.18	28.40 ± 4.00	0.089 *
**Pre-pregnancy BMI (kg/m^2^)**	33.08 [31.32–36.28] A	34.55 [31.24–35.99] A	21.31 [18.73–23.66] B	23.71 [21.91–24.83] B	<0.001 ^†^
**Pregnancy BMI (kg/m^2^)**	37.44 [32.54–40.20] A	37.91 [33.74–39.35] A	24.79 [22.39–26.56] B	26.04 [25.07–30.09] B	**<0.001 ^†^**
**BMI after pregnancy (kg/m^2^)**	34.49 [29.39–36.45] A	35.00 [31.64–36.00] A	22.61 [20.81–24.91] B	24.05 [22.65–25.21] B	**<0.001 ^†^**
**Dental plaque (%)**	77.74 ± 18.48	65.70 ± 28.30	77.19 ± 21.57	67.92 ± 25.26	0.565 *
**BOP (%)**	69.98 ± 17.90 A	14.28 ± 14.33 B	74.74 ± 13.17 A	12.84 ± 13.13 B	<0.001 *
**PPD (mm)**	2.65 [2.45–2.81] A	2.04 [1.96–2.10] B	2.56 [2.42–2.79] A	2.02 [2.00–2.16] B	**<0.001 ^†^**
**CAL (mm)**	2.66 [2.45–2.82] A	2.07 [2.02–2.10] B	2.58 [2.44–2.79] A	2.03 [2.00–2.16] B	**<0.001 ^†^**

OP, obesity and periodontitis; OWP, obesity without periodontitis; NP normal BMI and periodontitis; NWP, normal BMI without periodontitis; SD, standard deviation; *p*, significance level; BMI, body mass index; BOP, bleeding on probing; PPD, probing pocket depth; CAL, clinical attachment level; * ANOVA (post hoc test: Scheffé); ^†^ Kruskal–Wallis (post hoc test: Dunn). Different letters mean statistically significant differences between groups. Bold values mean significance level lower than 5%.

## Data Availability

Data presented in this study are available in the article and Supplementary Materials. In addition, data will be made available upon request to the corresponding authors.

## Supplementary Materials

The following supporting information can be downloaded at https://www.mdpi.com/article/10.3390/metabo12111091/s1. Supplementary file S1 is composed of Table S1: (A) Proteins identified in saliva of OP and OWP during T1 and their differences in expression; (B) Proteins identified in saliva of NP and NWP during T1 and their differences in expression; (C) Proteins identified in saliva of OP and NP during T1 and their differences in expression; (D) Proteins identified in saliva of OWP and NWP during T1 and their differences in expression. Supplementary file S2 is composed of Table S2: (A) Proteins identified in saliva of OP and OWP during T2 and their differences in expression; (B) Proteins identified in saliva of NP and NWP during T2 and their differences in expression; (C) Proteins identified in saliva of OP and NP during T2 and their differences in expression; (D) Proteins identified in saliva of OWP and NWP during T2 and their differences in expression. Supplementary file S3 is composed of Table S3: (A) Proteins identified in saliva in OP and their differences in expression during T1 and T2; (B) Proteins identified in saliva of OWP and their differences in expression during T1 and T2; (C) Proteins identified in saliva from NP and their differences in expression during T1 and T2; (D) Proteins identified in saliva of NWP and their differences in expression during T1 and T2. Figure S1: Functional analysis of the distribution of proteins identified with differential expression between groups in T1. Protein categories based on GO annotation of the broad Biological Process, Molecular Function, Immune System Process, and Cell Component. Terms of significance (Kappa = 0.04) and distribution according to the percentage of the number of associated genes. The number of access of proteins was provided by UNIPROT. The gene ontology was evaluated according to the ClueGo^®^ plugins of the software Cytoscape^®^ 3.7.2. Figure S2: Functional analysis of the distribution of proteins identified with differential expression between groups in T2. Protein categories based on GO annotation of the broad Biological Process, Molecular Function, Immune System Process, and Cell Component. Terms of significance (Kappa = 0.04) and distribution according to the percentage of the number of associated genes. The number of access of proteins was provided by UNIPROT. The gene ontology was evaluated according to the ClueGo^®^ plugins of the software Cytoscape^®^ 3.7.2. Figure S3: Functional analysis of the distribution of proteins identified with differential expression between periods for each group. Protein categories based on GO annotation of the broad Biological Process, Molecular Function, Immune System Process, and Cell Component. Terms of significance (Kappa = 0.04) and distribution according to the percentage of the number of associated genes. The number of access of proteins was provided by UNIPROT. The gene ontology was evaluated according to the ClueGo^®^ plugins of the software Cytoscape^®^ 3.7.2.
