# Supplementary material for: Label-Free Quantitative Proteomic Analysis Reveals Inflammatory Pattern Associated with Obesity and Periodontitis in Pregnant Women
Source: Metabolites. 2022 Nov 10;12(11):1091. doi: 10.3390/metabo12111091 (PMC9692340; doi:10.3390/metabo12111091)
Supplement: Supplementary file 1 [file metabolites-12-01091-s001.zip › Supplementary file S1.pdf]

S1–Table A. Proteins identified in saliva of OP and OWP during T1 and their differences in expression

Accession number	Protein name	Score	Ratio OP/OWP	Log(e)	SD	*p*	Expression differences
**P02808**	**Statherin**	**23896**	**29.67**	**3.39**	**0.11**	**< 0.01**	↑
**P69905**	**Hemoglobin subunit alpha**	**80**	**10.38**	**2.34**	**0.02**	**< 0.01**	↑
**P68871**	**Hemoglobin subunit beta**	**513**	**10.38**	**2.34**	**0.01**	**< 0.01**	↑
**P14780**	**Matrix metalloproteinase-9**	**179**	**6.62**	**1.89**	**0.07**	**< 0.01**	↑
**P69891**	**Hemoglobin subunit gamma-1**	**179**	**5.87**	**1.77**	**0.22**	**0.01**	↑
**P02100**	**Hemoglobin subunit epsilon**	**179**	**5.64**	**1.73**	**0.21**	**0.01**	↑
**P69892**	**Hemoglobin subunit gamma-2**	**179**	**5.16**	**1.64**	**0.09**	**< 0.01**	↑
**P26038**	**Moesin**	**110**	**4.95**	**1.60**	**0.14**	**< 0.01**	↑
**A8K2U0**	**Alpha-2-macroglobulin-like protein 1**	**20**	**3.39**	**1.22**	**0.27**	**< 0.01**	↑
**P10599**	**Thioredoxin**	**461**	**2.97**	**1.09**	**0.18**	**< 0.01**	↑
**P07108**	**Acyl-CoA-binding protein**	**155**	**2.86**	**1.05**	**0.12**	**< 0.01**	↑
**P07864**	**L-lactate dehydrogenase C chain**	**325**	**2.61**	**0.96**	**0.14**	**0.01**	↑
**Q6ZMR3**	**L-lactate dehydrogenase A-like 6A**	**351**	**2.56**	**0.94**	**0.15**	**0.01**	↑
**P20742**	**Pregnancy zone protein**	**25**	**2.48**	**0.91**	**0.10**	**< 0.01**	↑
**P07195**	**L-lactate dehydrogenase B chain**	**325**	**2.44**	**0.89**	**0.20**	**0.01**	↑
**Q6P5S2**	**Protein LEG1 homolog**	**270**	**2.44**	**0.89**	**0.19**	**< 0.01**	↑
**P02814**	**Submaxillary gland androgen-regulated protein 3B**	**8043**	**2.39**	**0.87**	**0.03**	**< 0.01**	↑
**P02787**	**Serotransferrin**	**207**	**2.36**	**0.86**	**0.02**	**< 0.01**	↑
**P00338**	**L-lactate dehydrogenase A chain**	**877**	**2.34**	**0.85**	**0.09**	**< 0.01**	↑
**P06396**	**Gelsolin**	**272**	**2.23**	**0.80**	**0.09**	**< 0.01**	↑
**P0DOX8**	**Immunoglobulin lambda-1 light chain**	**115**	**2.18**	**0.78**	**0.05**	**< 0.01**	↑
**P02768**	**Albumin**	**3664**	**2.16**	**0.77**	**0.01**	**< 0.01**	↑
**P00739**	**Haptoglobin-related protein**	**23**	**2.08**	**0.73**	**0.10**	**< 0.01**	↑
**P01023**	**Alpha-2-macroglobulin**	**44**	**2.00**	**0.69**	**0.05**	**< 0.01**	↑
P05109	Protein S100-A8	5810	1.95	0.67	0.07	< 0.01	↑
P00450	Ceruloplasmin	882	1.93	0.66	0.07	< 0.01	↑
P22079	Lactoperoxidase	55	1.93	0.66	0.05	< 0.01	↑
P52566	Rho GDP-dissociation inhibitor 2	206	1.92	0.65	0.10	< 0.01	↑
P01034	Cystatin-C	444	1.86	0.62	0.05	< 0.01	↑
P01019	Angiotensinogen	193	1.84	0.61	0.13	< 0.01	↑
P17066	Heat shock 70 kDa protein 6	270	1.82	0.60	0.14	< 0.01	↑
P15516	Histatin-3	1165	1.79	0.58	0.15	< 0.01	↑
P48741	Putative heat shock 70 kDa protein 7	270	1.77	0.57	0.15	< 0.01	↑
P01871	Immunoglobulin heavy constant mu	379	1.72	0.54	0.10	< 0.01	↑
P02675	Fibrinogen beta chain	2032	1.68	0.52	0.07	< 0.01	↑
P00738	Haptoglobin	211	1.67	0.51	0.08	< 0.01	↑
P54652	Heat shock-related 70 kDa protein 2	245	1.65	0.50	0.18	< 0.01	↑
P02042	Hemoglobin subunit delta	201	1.65	0.50	0.11	< 0.01	↑
P11142	Heat shock cognate 71 kDa protein	245	1.63	0.49	0.14	< 0.01	↑

P0DOX6	Immunoglobulin mu heavy chain	373	1.62	0.48	0.14	0.01	↑
A0M8Q6	Immunoglobulin lambda constant 7	201	1.60	0.47	0.05	< 0.01	↑
P0CF74	Immunoglobulin lambda constant 6	201	1.57	0.45	0.07	< 0.01	↑
Q01518	Adenylyl cyclase-associated protein 1	278	1.52	0.42	0.16	0.01	↑
P0DMV9	Heat shock 70 kDa protein 1B	330	1.52	0.42	0.13	0.01	↑
P0DOY2	Immunoglobulin lambda constant 2	201	1.52	0.42	0.04	< 0.01	↑
P0DOY3	Immunoglobulin lambda constant 3	201	1.52	0.42	0.05	< 0.01	↑
P01024	Complement C3	25	1.51	0.41	0.04	< 0.01	↑
P60174	Triosephosphate isomerase	520	1.49	0.40	0.22	0.04	↑
P02766	Transthyretin	255	1.48	0.39	0.16	0.04	↑
B9A064	Immunoglobulin lambda-like polypeptide 5	115	1.46	0.38	0.07	< 0.01	↑
P0CG04	Immunoglobulin lambda constant 1	115	1.43	0.36	0.06	< 0.01	↑
P0DMV8	Heat shock 70 kDa protein 1A	349	1.42	0.35	0.12	< 0.01	↑
P23280	Carbonic anhydrase 6	499	1.38	0.32	0.11	0.01	↑
P02810	Salivary acidic proline-rich phosphoprotein 1/2	1111	1.38	0.32	0.03	< 0.01	↑
P34931	Heat shock 70 kDa protein 1-like	299	1.31	0.27	0.09	0.01	↑
P0DOX5	Immunoglobulin gamma-1 heavy chain	603	1.23	0.21	0.03	< 0.01	↑
P01860	Immunoglobulin heavy constant gamma 3	108	1.22	0.20	0.05	< 0.01	↑
P0DOX7	Immunoglobulin kappa light chain	185	1.20	0.18	0.07	0.02	↑
P01857	Immunoglobulin heavy constant gamma 1	603	1.19	0.17	0.04	< 0.01	↑
P0DTE7	Alpha-amylase 1B	12079	1.17	0.16	0.01	< 0.01	↑
P06744	Glucose-6-phosphate isomerase	436	1.17	0.16	0.08	0.02	↑
P04746	Pancreatic alpha-amylase	8827	1.17	0.16	0.01	< 0.01	↑
P0DTE8	Alpha-amylase 1C	12079	1.16	0.15	0.01	< 0.01	↑
P0DUB6	Alpha-amylase 1A	12079	1.09	0.09	0.01	< 0.01	↑
P19961	Alpha-amylase 2B	10632	1.07	0.07	0.01	< 0.01	↑
P01877	Immunoglobulin heavy constant alpha 2	1437	0.90	-0.11	0.01	< 0.01	↓
P01876	Immunoglobulin heavy constant alpha 1	1734	0.86	-0.15	0.01	< 0.01	↓
P06733	Alpha-enolase	262	0.82	-0.20	0.06	< 0.01	↓
P61626	Lysozyme C	3669	0.81	-0.21	0.04	< 0.01	↓
Q96DR5	BPI fold-containing family A member 2	186	0.75	-0.29	0.05	< 0.01	↓
P06702	Protein S100-A9	1584	0.75	-0.29	0.05	< 0.01	↓
Q9UBG3	Cornulin	100	0.73	-0.31	0.11	0.03	↓
P02671	Fibrinogen alpha chain	360	0.73	-0.31	0.12	0.01	↓
Q9UBC9	Small proline-rich protein 3	1377	0.70	-0.35	0.21	< 0.01	↓
P52209	6-phosphogluconate dehydrogenase. decarboxylating	159	0.68	-0.39	0.14	< 0.01	↓
Q9UGM3	Deleted in malignant brain tumors 1 protein	35	0.67	-0.40	0.06	< 0.01	↓
P01036	Cystatin-S	4223	0.64	-0.45	0.03	< 0.01	↓
P14618	Pyruvate kinase PKM	118	0.61	-0.50	0.06	< 0.01	↓
P01009	Alpha-1-antitrypsin	75	0.60	-0.51	0.05	< 0.01	↓
Q8TAX7	Mucin-7	330	0.59	-0.53	0.07	< 0.01	↓
Q96DA0	Zymogen granule protein 16 homolog B	1084	0.59	-0.53	0.02	< 0.01	↓
Q9H299	SH3 domain-binding glutamic acid-rich-like protein 3	413	0.56	-0.58	0.22	0.01	↓

P01833	Polymeric immunoglobulin receptor	2946	0.55	-0.60	0.02	< 0.01	↓
P04075	Fructose-bisphosphate aldolase A	177	0.53	-0.64	0.09	< 0.01	↓
P09228	Cystatin-SA	513	0.51	-0.68	0.02	< 0.01	↓
P80188	Neutrophil gelatinase-associated lipocalin	2719	0.51	-0.68	0.06	< 0.01	↓
**Q5VSP4**	**Putative lipocalin 1-like protein 1**	**662**	**0.50**	**-0.70**	**0.06**	**< 0.01**	↓
**P06870**	**Kallikrein-1**	**107**	**0.43**	**-0.84**	**0.08**	**< 0.01**	↓
**P01037**	**Cystatin-SN**	**3683**	**0.39**	**-0.95**	**0.02**	**< 0.01**	↓
**P02790**	**Hemopexin**	**125**	**0.37**	**-1.00**	**0.09**	**< 0.01**	↓
**P12273**	**Prolactin-inducible protein**	**3571**	**0.36**	**-1.01**	**0.03**	**< 0.01**	↓
**P24158**	**Myeloblastin**	**379**	**0.35**	**-1.04**	**0.11**	**< 0.01**	↓
**P25311**	**Zinc-alpha-2-glycoprotein**	**101**	**0.35**	**-1.05**	**0.19**	**< 0.01**	↓
**P20061**	**Transcobalamin-1**	**127**	**0.32**	**-1.14**	**0.14**	**< 0.01**	↓
**P28325**	**Cystatin-D**	**114**	**0.25**	**-1.40**	**0.08**	**< 0.01**	↓
**P04080**	**Cystatin-B**	**240**	**0.23**	**-1.47**	**0.05**	**< 0.01**	↓
**P59665**	**Neutrophil defensin 1**	**2405**	**0.23**	**-1.47**	**0.05**	**< 0.01**	↓
**P59666**	**Neutrophil defensin 3**	**2405**	**0.23**	**-1.47**	**0.04**	**< 0.01**	↓
**P04406**	**Glyceraldehyde-3-phosphate dehydrogenase**	**250**	**0.22**	**-1.51**	**0.04**	**< 0.01**	↓
**P04280**	**Basic salivary proline-rich protein 1**	**85**	**0.22**	**-1.52**	**0.03**	**< 0.01**	↓
**P02812**	**Basic salivary proline-rich protein 2**	**85**	**0.22**	**-1.52**	**0.03**	**< 0.01**	↓
**P02788**	**Lactotransferrin**	**1044**	**0.19**	**-1.65**	**0.03**	**< 0.01**	↓
**Q14508**	**WAP four-disulfide core domain protein 2**	**1630**	**0.08**	**-2.50**	**0.06**	**< 0.01**	↓
P31947	14-3-3 protein sigma	448	-	-	-	-	OP
Q6P587	Acylpyruvase FAHD1. mitochondrial	568	-	-	-	-	OP
P12814	Alpha-actinin-1	62	-	-	-	-	OP
O43707	Alpha-actinin-4	50	-	-	-	-	OP
Q96LR9	Apolipoprotein L domain-containing protein 1	70	-	-	-	-	OP
Q8NHQ9	ATP-dependent RNA helicase DDX55	141	-	-	-	-	OP
P27482	Calmodulin-like protein 3	227	-	-	-	-	OP
Q92616	eIF-2-alpha kinase activator GCN1	45	-	-	-	-	OP
Q9P2K8	eIF-2-alpha kinase GCN2	306	-	-	-	-	OP
Q5W0V3	FHF complex subunit HOOK interacting protein 2A	152	-	-	-	-	OP
Q08380	Galectin-3-binding protein	99	-	-	-	-	OP
Q9UJ14	Glutathione hydrolase 7	114	-	-	-	-	OP
P06737	Glycogen phosphorylase. liver form	45	-	-	-	-	OP
P15515	Histatin-1	3253	-	-	-	-	OP
P01764	Immunoglobulin heavy variable 3-23	458	-	-	-	-	OP
P01768	Immunoglobulin heavy variable 3-30	458	-	-	-	-	OP
P0DP02	Immunoglobulin heavy variable 3-30-3	458	-	-	-	-	OP
P0DP03	Immunoglobulin heavy variable 3-30-5	458	-	-	-	-	OP
P01772	Immunoglobulin heavy variable 3-33	458	-	-	-	-	OP
P01767	Immunoglobulin heavy variable 3-53	458	-	-	-	-	OP
A0A0C4DH42	Immunoglobulin heavy variable 3-66	458	-	-	-	-	OP
A0A0B4J1X5	Immunoglobulin heavy variable 3-74	458	-	-	-	-	OP
P04433	Immunoglobulin kappa variable 3-11	692	-	-	-	-	OP

A0A0A0MRZ8	Immunoglobulin kappa variable 3D-11	692	-	-	-	-	OP
Q8WYH8	Inhibitor of growth protein 5	568	-	-	-	-	OP
Q5T7N2	LINE-1 type transposase domain-containing protein 1	35	-	-	-	-	OP
P01033	Metalloproteinase inhibitor 1	175	-	-	-	-	OP
Q02817	Mucin-2	45	-	-	-	-	OP
Q8NCY6	Myb/SANT-like DNA-binding domain-containing protein 4	165	-	-	-	-	OP
P80303	Nucleobindin-2	79	-	-	-	-	OP
Q8NGQ2	Olfactory receptor 6Q1	201	-	-	-	-	OP
Q14651	Plastin-1	88	-	-	-	-	OP
Q8N6L0	Protein KASH5	62	-	-	-	-	OP
Q9Y5F8	Protocadherin gamma-B7	47	-	-	-	-	OP
P50120	Retinol-binding protein 2	390	-	-	-	-	OP
P35326	Small proline-rich protein 2A	711	-	-	-	-	OP
P35325	Small proline-rich protein 2B	950	-	-	-	-	OP
P22532	Small proline-rich protein 2D	950	-	-	-	-	OP
P22531	Small proline-rich protein 2E	781	-	-	-	-	OP
Q96RM1	Small proline-rich protein 2F	243	-	-	-	-	OP
Q9BYE4	Small proline-rich protein 2G	539	-	-	-	-	OP
Q14515	SPARC-like protein 1	31	-	-	-	-	OP
P49770	Translation initiation factor eIF-2B subunit beta	36	-	-	-	-	OP
P36537	UDP-glucuronosyltransferase 2B10	23	-	-	-	-	OP
Q9BY64	UDP-glucuronosyltransferase 2B28	23	-	-	-	-	OP
Q9UJU3	Zinc finger protein 112	47	-	-	-	-	OP
Q8TDL5	BPI fold-containing family B member 1	58	-	-	-	-	OWP
Q5SW79	Centrosomal protein of 170 kDa	90	-	-	-	-	OWP
O15078	Centrosomal protein of 290 kDa	162	-	-	-	-	OWP
P10909	Clusterin	191	-	-	-	-	OWP
Q9Y281	Cofilin-2	139	-	-	-	-	OWP
A2RUR9	Coiled-coil domain-containing protein 144A	52	-	-	-	-	OWP
Q3MJ40	Coiled-coil domain-containing protein 144B	45	-	-	-	-	OWP
P0C0L4	Complement C4-A	72	-	-	-	-	OWP
P0C0L5	Complement C4-B	70	-	-	-	-	OWP
Q02487	Desmocollin-2	76	-	-	-	-	OWP
Q92993	Histone acetyltransferase KAT5	157	-	-	-	-	OWP
A0A075B6P5	Immunoglobulin kappa variable 2-28	184	-	-	-	-	OWP
A2NJV5	Immunoglobulin kappa variable 2-29	184	-	-	-	-	OWP
P06310	Immunoglobulin kappa variable 2-30	184	-	-	-	-	OWP
A0A087WW87	Immunoglobulin kappa variable 2-40	184	-	-	-	-	OWP
A0A0A0MRZ7	Immunoglobulin kappa variable 2D-26	184	-	-	-	-	OWP
P01615	Immunoglobulin kappa variable 2D-28	184	-	-	-	-	OWP
A0A075B6S2	Immunoglobulin kappa variable 2D-29	184	-	-	-	-	OWP
A0A075B6S6	Immunoglobulin kappa variable 2D-30	184	-	-	-	-	OWP
P01614	Immunoglobulin kappa variable 2D-40	184	-	-	-	-	OWP

O14782	Kinesin-like protein KIF3C	52	-	-	-	-	OWP
O95274	Ly6/PLAUR domain-containing protein 3	144	-	-	-	-	OWP
O60449	Lymphocyte antigen 75	18	-	-	-	-	OWP
P40925	Malate dehydrogenase. cytoplasmic	117	-	-	-	-	OWP
O75556	Mammaglobin-B	522	-	-	-	-	OWP
O00255	Menin	47	-	-	-	-	OWP
Q8NEM0	Microcephalin	50	-	-	-	-	OWP
O43318	Mitogen-activated protein kinase kinase kinase 7	42	-	-	-	-	OWP
P05164	Myeloperoxidase	87	-	-	-	-	OWP
O75161	Nephrocystin-4	62	-	-	-	-	OWP
P30041	Peroxiredoxin-6	153	-	-	-	-	OWP
Q8TBY8	Polyamine-modulated factor 1-binding protein 1	45	-	-	-	-	OWP
P07602	Prosaposin	96	-	-	-	-	OWP
P02760	Protein AMBP	149	-	-	-	-	OWP
P07237	Protein disulfide-isomerase	180	-	-	-	-	OWP
Q6NUI1	Putative coiled-coil domain-containing protein 144 N-terminal-like	39	-	-	-	-	OWP
Q8IYA2	Putative coiled-coil domain-containing protein 144C	50	-	-	-	-	OWP
P35241	Radixin	88	-	-	-	-	OWP
P35249	Replication factor C subunit 4	66	-	-	-	-	OWP
Q8N392	Rho GTPase-activating protein 18	59	-	-	-	-	OWP
Q96QB1	Rho GTPase-activating protein 7	85	-	-	-	-	OWP
Q9NTJ3	Structural maintenance of chromosomes protein 4	46	-	-	-	-	OWP
Q6PKC3	Thioredoxin domain-containing protein 11	17	-	-	-	-	OWP
Q9Y4F4	TOG array regulator of axonemal microtubules protein 1	52	-	-	-	-	OWP
Q9BXT4	Tudor domain-containing protein 1	19	-	-	-	-	OWP
P36941	Tumor necrosis factor receptor superfamily member 3	87	-	-	-	-	OWP
O94966	Ubiquitin carboxyl-terminal hydrolase 19	20	-	-	-	-	OWP
P04004	Vitronectin	65	-	-	-	-	OWP
Q96KN7	X-linked retinitis pigmentosa GTPase regulator-interacting protein 1	61	-	-	-	-	OWP
Q63HK3	Zinc finger protein with KRAB and SCAN domains 2	43	-	-	-	-	OWP
Q01469	Fatty acid-binding protein 5	707	1.65	0.50	0.29	0.92	SE
P30613	Pyruvate kinase PKLR	29	1.63	0.49	0.44	0.70	SE
P11021	Endoplasmic reticulum chaperone BiP	207	1.54	0.43	0.21	0.95	SE
P37837	Transaldolase	203	1.51	0.41	0.30	0.81	SE
Q9Y536	Peptidyl-prolyl cis-trans isomerase A-like 4A	40	1.32	0.28	0.40	0.49	SE
P03973	Antileukoproteinase	459	1.31	0.27	0.18	0.88	SE
P07205	Phosphoglycerate kinase 2	127	1.28	0.25	0.18	0.82	SE
P02679	Fibrinogen gamma chain	2273	1.26	0.23	0.15	0.90	SE
P29401	Transketolase	95	1.14	0.13	0.22	0.62	SE
P61769	Beta-2-microglobulin	457	1.12	0.11	0.11	0.88	SE

P07737	Profilin-1	449	1.11	0.10	0.07	0.89	SE
P18510	Interleukin-1 receptor antagonist protein	241	1.09	0.09	0.22	0.60	SE
P00558	Phosphoglycerate kinase 1	35	1.07	0.07	0.11	0.70	SE
P01861	Immunoglobulin heavy constant gamma 4	42	1.06	0.06	0.10	0.74	SE
P02765	Alpha-2-HS-glycoprotein	579	1.05	0.05	0.23	0.56	SE
P02647	Apolipoprotein A-I	141	1.05	0.05	0.08	0.70	SE
P62937	Peptidyl-prolyl cis-trans isomerase A	151	1.05	0.05	0.17	0.57	SE
P01591	Immunoglobulin J chain	1073	1.04	0.04	0.06	0.81	SE
P13796	Plastin-2	164	1.03	0.03	0.12	0.50	SE
P0DOX2	Immunoglobulin alpha-2 heavy chain	1386	1.02	0.02	0.02	0.79	SE
P01859	Immunoglobulin heavy constant gamma 2	42	1.01	0.01	0.14	0.57	SE
P01834	Immunoglobulin kappa constant	343	0.99	-0.01	0.03	0.34	SE
P31025	Lipocalin-1	1422	0.97	-0.03	0.04	0.30	SE
P09104	Gamma-enolase	105	0.95	-0.05	0.13	0.36	SE
P13929	Beta-enolase	105	0.93	-0.07	0.09	0.23	SE
P02774	Vitamin D-binding protein	1144	0.90	-0.11	0.11	0.18	SE
P09211	Glutathione S-transferase P	330	0.86	-0.15	0.27	0.36	SE
Q16378	Proline-rich protein 4	903	0.86	-0.15	0.65	0.20	SE
P02763	Alpha-1-acid glycoprotein 1	756	0.84	-0.17	0.70	0.23	SE
P54108	Cysteine-rich secretory protein 3	245	0.76	-0.28	0.20	0.12	SE
P13797	Plastin-3	34	0.74	-0.30	0.39	0.22	SE
P23528	Cofilin-1	930	0.68	-0.38	0.54	0.24	SE

Note: Ratio OP/OWP (fold change) = ratio between pregnant women with obesity and periodontitis and control group proteins (pregnant women with obesity but without periodontitis); Log(e) ("e" is a constant = 2.71); SD, standard deviation; *p*, statistical significance (adjusted by False Discovery Rate–FDR = 4); ↑ = up-regulated (1-p > 0.95); ↓ = down-regulated (p < 0.05); SE = similar expression compared to control group; bold lines refer to up- or down-regulated proteins by more than 2-fold

S1–Table B. Proteins identified in saliva of NP and NWP during T1 and their differences in expression

**Accession number**	**Protein name**	**Score**	**Ratio NP/NWP**	**Log(e)**	**SD**	***p***	**Expression differences**
**Q9UGM3**	**Deleted in malignant brain tumors 1 protein**	**393**	**5.58**	**1.72**	**0.03**	**< 0.01**	↑
**P06702**	**Protein S100-A9**	**181**	**5.26**	**1.66**	**0.03**	**< 0.01**	↑
**P01023**	**Alpha-2-macroglobulin**	**27**	**4.44**	**1.49**	**0.08**	**< 0.01**	↑
**P68032**	**Actin. alpha cardiac muscle 1**	**884**	**4.22**	**1.44**	**0.03**	**< 0.01**	↑
**P63267**	**Actin. gamma-enteric smooth muscle**	**884**	**4.10**	**1.41**	**0.02**	**< 0.01**	↑
**Q562R1**	**Beta-actin-like protein 2**	**626**	**4.01**	**1.39**	**0.03**	**< 0.01**	↑
**P01857**	**Immunoglobulin heavy constant gamma 1**	**184**	**3.90**	**1.36**	**0.04**	**< 0.01**	↑
**P01860**	**Immunoglobulin heavy constant gamma 3**	**103**	**3.86**	**1.35**	**0.07**	**< 0.01**	↑
**P0DOX5**	**Immunoglobulin gamma-1 heavy chain**	**184**	**3.82**	**1.34**	**0.04**	**< 0.01**	↑
**P02042**	**Hemoglobin subunit delta**	**151**	**3.78**	**1.33**	**0.06**	**< 0.01**	↑
**P05109**	**Protein S100-A8**	**250**	**3.74**	**1.32**	**0.04**	**< 0.01**	↑
**P01024**	**Complement C3**	**45**	**3.74**	**1.32**	**0.15**	**< 0.01**	↑

**P02647**	**Apolipoprotein A-I**	**171**	**3.22**	**1.17**	**0.05**	**< 0.01**	↑
**Q9H299**	**SH3 domain-binding glutamic acid-rich-like protein 3**	**305**	**3.13**	**1.14**	**0.34**	**< 0.01**	↑
**P02790**	**Hemopexin**	**108**	**3.03**	**1.11**	**0.09**	**< 0.01**	↑
**P01859**	**Immunoglobulin heavy constant gamma 2**	**30**	**2.97**	**1.09**	**0.11**	**< 0.01**	↑
**P07737**	**Profilin-1**	**859**	**2.89**	**1.06**	**0.08**	**< 0.01**	↑
**P24158**	**Myeloblastin**	**177**	**2.83**	**1.04**	**0.23**	**< 0.01**	↑
**A5A3E0**	**POTE ankyrin domain family member F**	**337**	**2.72**	**1.00**	**0.05**	**< 0.01**	↑
**P60709**	**Actin. cytoplasmic 1**	**1017**	**2.61**	**0.96**	**0.03**	**< 0.01**	↑
**P04075**	**Fructose-bisphosphate aldolase A**	**182**	**2.59**	**0.95**	**0.08**	**< 0.01**	↑
**P02787**	**Serotransferrin**	**391**	**2.56**	**0.94**	**0.04**	**< 0.01**	↑
**P63261**	**Actin. cytoplasmic 2**	**1014**	**2.51**	**0.92**	**0.03**	**< 0.01**	↑
**P62736**	**Actin. aortic smooth muscle**	**884**	**2.16**	**0.77**	**0.04**	**< 0.01**	↑
**P02100**	**Hemoglobin subunit epsilon**	**119**	**2.14**	**0.76**	**0.15**	**< 0.01**	↑
**P69892**	**Hemoglobin subunit gamma-2**	**119**	**2.12**	**0.75**	**0.18**	**< 0.01**	↑
**P13796**	**Plastin-2**	**123**	**2.08**	**0.73**	**0.09**	**< 0.01**	↑
**P69891**	**Hemoglobin subunit gamma-1**	**119**	**2.03**	**0.71**	**0.16**	**< 0.01**	↑
**P10599**	**Thioredoxin**	**4451**	**2.01**	**0.70**	**0.13**	**< 0.01**	↑
**P02768**	**Albumin**	**6357**	**2.00**	**0.69**	**0.01**	**< 0.01**	↑
P30613	Pyruvate kinase PKLR	91	1.95	0.67	0.28	< 0.01	↑
Q6S8J3	POTE ankyrin domain family member E	337	1.92	0.65	0.07	< 0.01	↑
P68871	Hemoglobin subunit beta	151	1.92	0.65	0.10	< 0.01	↑
A8K2U0	Alpha-2-macroglobulin-like protein 1	109	1.88	0.63	0.16	< 0.01	↑
P14618	Pyruvate kinase PKM	135	1.82	0.60	0.12	< 0.01	↑
P0CG38	POTE ankyrin domain family member I	83	1.79	0.58	0.06	< 0.01	↑
P68133	Actin. alpha skeletal muscle	884	1.77	0.57	0.05	< 0.01	↑
P29401	Transketolase	89	1.75	0.56	0.20	< 0.01	↑
P07108	Acyl-CoA-binding protein	297	1.68	0.52	0.21	< 0.01	↑
P0DOX7	Immunoglobulin kappa light chain	276	1.67	0.51	0.07	< 0.01	↑
P0CG39	POTE ankyrin domain family member J	83	1.65	0.50	0.10	< 0.01	↑
P01861	Immunoglobulin heavy constant gamma 4	122	1.63	0.49	0.12	< 0.01	↑
P37837	Transaldolase	199	1.62	0.48	0.16	< 0.01	↑
P01871	Immunoglobulin heavy constant mu	118	1.62	0.48	0.08	< 0.01	↑
P0DMV9	Heat shock 70 kDa protein 1B	116	1.54	0.43	0.13	< 0.01	↑
P00738	Haptoglobin	132	1.51	0.41	0.13	< 0.01	↑
P01834	Immunoglobulin kappa constant	1907	1.49	0.40	0.04	< 0.01	↑
P0DOX6	Immunoglobulin mu heavy chain	118	1.49	0.40	0.09	< 0.01	↑
P06744	Glucose-6-phosphate isomerase	96	1.49	0.40	0.20	0.04	↑
P34931	Heat shock 70 kDa protein 1-like	90	1.48	0.39	0.13	< 0.01	↑
P0DMV8	Heat shock 70 kDa protein 1A	116	1.46	0.38	0.13	< 0.01	↑
Q9BYX7	Putative beta-actin-like protein 3	254	1.38	0.32	0.07	< 0.01	↑
P09104	Gamma-enolase	124	1.32	0.28	0.11	< 0.01	↑
P02814	Submaxillary gland androgen-regulated protein 3B	51408	1.23	0.21	0.03	< 0.01	↑
P0DOY2	Immunoglobulin lambda constant 2	4884	1.17	0.16	0.07	0.02	↑

P0CF74	Immunoglobulin lambda constant 6	3630	1.15	0.14	0.06	< 0.01	↑
P04080	Cystatin-B	5550	1.12	0.11	0.06	0.03	↑
Q96DA0	Zymogen granule protein 16 homolog B	22215	1.08	0.08	0.02	< 0.01	↑
P22079	Lactoperoxidase	617	0.79	-0.23	0.09	0.02	↓
Q6P5S2	Protein LEG1 homolog	2046	0.79	-0.24	0.11	0.02	↓
P0DOX8	Immunoglobulin lambda-1 light chain	3256	0.70	-0.35	0.07	< 0.01	↓
B9A064	Immunoglobulin lambda-like polypeptide 5	3303	0.70	-0.36	0.07	< 0.01	↓
P0CG04	Immunoglobulin lambda constant 1	3086	0.69	-0.37	0.08	< 0.01	↓
P35326	Small proline-rich protein 2A	733	0.69	-0.37	0.15	0.01	↓
Q8N4F0	BPI fold-containing family B member 2	612	0.66	-0.42	0.08	< 0.01	↓
Q96DR5	BPI fold-containing family A member 2	944	0.64	-0.44	0.07	< 0.01	↓
Q8TAX7	Mucin-7	1497	0.64	-0.44	0.04	< 0.01	↓
P01833	Polymeric immunoglobulin receptor	16349	0.63	-0.47	0.03	< 0.01	↓
P31025	Lipocalin-1	5807	0.61	-0.49	0.06	< 0.01	↓
P01876	Immunoglobulin heavy constant alpha 1	9432	0.61	-0.50	0.01	< 0.01	↓
Q16378	Proline-rich protein 4	3936	0.61	-0.50	0.13	< 0.01	↓
P28325	Cystatin-D	333	0.60	-0.51	0.05	< 0.01	↓
P01036	Cystatin-S	16480	0.59	-0.53	0.02	< 0.01	↓
P12273	Prolactin-inducible protein	25257	0.59	-0.53	0.02	< 0.01	↓
P22531	Small proline-rich protein 2E	373	0.59	-0.53	0.23	0.01	↓
P35325	Small proline-rich protein 2B	373	0.58	-0.55	0.22	0.03	↓
P22532	Small proline-rich protein 2D	373	0.58	-0.55	0.23	0.01	↓
Q9BYE4	Small proline-rich protein 2G	347	0.58	-0.55	0.26	0.03	↓
P0DOY3	Immunoglobulin lambda constant 3	4884	0.55	-0.59	0.05	< 0.01	↓
P61769	Beta-2-microglobulin	132	0.52	-0.66	0.22	< 0.01	↓
**P01591**	**Immunoglobulin J chain**	**9177**	**0.44**	**-0.81**	**0.05**	**< 0.01**	↓
**P69905**	**Hemoglobin subunit alpha**	**95**	**0.43**	**-0.85**	**0.13**	**< 0.01**	↓
**P04746**	**Pancreatic alpha-amylase**	**28592**	**0.41**	**-0.89**	**0.01**	**< 0.01**	↓
**P0DUB6**	**Alpha-amylase 1A**	**41555**	**0.39**	**-0.94**	**0.01**	**< 0.01**	↓
**P03973**	**Antileukoproteinase**	**1419**	**0.37**	**-0.99**	**0.13**	**< 0.01**	↓
**P01037**	**Cystatin-SN**	**10770**	**0.37**	**-0.99**	**0.01**	**< 0.01**	↓
**P01877**	**Immunoglobulin heavy constant alpha 2**	**4178**	**0.37**	**-1.00**	**0.02**	**< 0.01**	↓
**P0DOX2**	**Immunoglobulin alpha-2 heavy chain**	**4092**	**0.36**	**-1.01**	**0.02**	**< 0.01**	↓
**P01034**	**Cystatin-C**	**3385**	**0.36**	**-1.03**	**0.08**	**< 0.01**	↓
**P02788**	**Lactotransferrin**	**425**	**0.36**	**-1.03**	**0.04**	**< 0.01**	↓
**P09228**	**Cystatin-SA**	**3237**	**0.32**	**-1.13**	**0.03**	**< 0.01**	↓
**Q9UJ14**	**Glutathione hydrolase 7**	**123**	**0.26**	**-1.33**	**0.08**	**< 0.01**	↓
**P0DTE7**	**Alpha-amylase 1B**	**41555**	**0.26**	**-1.35**	**0.01**	**< 0.01**	↓
**P19961**	**Alpha-amylase 2B**	**35774**	**0.26**	**-1.35**	**0.01**	**< 0.01**	↓
**Q5VSP4**	**Putative lipocalin 1-like protein 1**	**4592**	**0.22**	**-1.51**	**0.04**	**< 0.01**	↓
**P0DTE8**	**Alpha-amylase 1C**	**41555**	**0.19**	**-1.67**	**0.01**	**< 0.01**	↓
**Q8TDL5**	**BPI fold-containing family B member 1**	**912**	**0.19**	**-1.68**	**0.07**	**< 0.01**	↓
**P02810**	**Salivary acidic proline-rich phosphoprotein 1/2**	**3931**	**0.17**	**-1.75**	**0.17**	**0.01**	↓
**P23280**	**Carbonic anhydrase 6**	**1177**	**0.10**	**-2.29**	**0.02**	**< 0.01**	↓
P27216	Annexin A13	392	-	-	-	-	NP

Q8NHQ9	ATP-dependent RNA helicase DDX55	169	-	-	-	-	NP
P0DP23	Calmodulin-1	252	-	-	-	-	NP
P0DP24	Calmodulin-2	252	-	-	-	-	NP
P0DP25	Calmodulin-3	252	-	-	-	-	NP
Q8NEL0	Coiled-coil domain-containing protein 54	185	-	-	-	-	NP
Q9UBG3	Cornulin	258	-	-	-	-	NP
Q99543	DnaJ homolog subfamily C member 2	51	-	-	-	-	NP
P15311	Ezrin	20	-	-	-	-	NP
Q9P2Q2	FERM domain-containing protein 4A	24	-	-	-	-	NP
P02675	Fibrinogen beta chain	511	-	-	-	-	NP
P02679	Fibrinogen gamma chain	118	-	-	-	-	NP
Q5RHP9	Glutamate-rich protein 3	41	-	-	-	-	NP
O14556	Glyceraldehyde-3-phosphate dehydrogenase. testis-specific	93	-	-	-	-	NP
Q8NBJ4	Golgi membrane protein 1	63	-	-	-	-	NP
P02008	Hemoglobin subunit zeta	710	-	-	-	-	NP
P01764	Immunoglobulin heavy variable 3-23	928	-	-	-	-	NP
P01768	Immunoglobulin heavy variable 3-30	928	-	-	-	-	NP
P0DP02	Immunoglobulin heavy variable 3-30-3	928	-	-	-	-	NP
P0DP03	Immunoglobulin heavy variable 3-30-5	928	-	-	-	-	NP
P01772	Immunoglobulin heavy variable 3-33	928	-	-	-	-	NP
P01767	Immunoglobulin heavy variable 3-53	928	-	-	-	-	NP
A0A0C4DH42	Immunoglobulin heavy variable 3-66	928	-	-	-	-	NP
A0A0B4J1X5	Immunoglobulin heavy variable 3-74	928	-	-	-	-	NP
P04433	Immunoglobulin kappa variable 3-11	578	-	-	-	-	NP
A0A0A0MRZ8	Immunoglobulin kappa variable 3D-11	578	-	-	-	-	NP
P00338	L-lactate dehydrogenase A chain	117	-	-	-	-	NP
Q6ZMR3	L-lactate dehydrogenase A-like 6A	56	-	-	-	-	NP
P07195	L-lactate dehydrogenase B chain	56	-	-	-	-	NP
P07864	L-lactate dehydrogenase C chain	56	-	-	-	-	NP
Q6B0I6	Lysine-specific demethylase 4D	104	-	-	-	-	NP
P14780	Matrix metalloproteinase-9	82	-	-	-	-	NP
P26038	Moesin	50	-	-	-	-	NP
Q02817	Mucin-2	20	-	-	-	-	NP
P80188	Neutrophil gelatinase-associated lipocalin	366	-	-	-	-	NP
P47874	Olfactory marker protein	102	-	-	-	-	NP
Q14651	Plastin-1	33	-	-	-	-	NP
Q9Y2S7	Polymerase delta-interacting protein 2	70	-	-	-	-	NP
P20742	Pregnancy zone protein	212	-	-	-	-	NP
Q9UQ80	Proliferation-associated protein 2G4	41	-	-	-	-	NP
Q6MZM9	Proline-rich protein 27	248	-	-	-	-	NP
P80511	Protein S100-A12	209	-	-	-	-	NP
P35241	Radixin	19	-	-	-	-	NP
Q6UWP8	Suprabasin	60	-	-	-	-	NP
P26639	Threonine--tRNA ligase 1. cytoplasmic	92	-	-	-	-	NP

Q9Y4F4	TOG array regulator of axonemal microtubules protein 1	97	-	-	-	-	NP
P51809	Vesicle-associated membrane protein 7	100	-	-	-	-	NP
P02774	Vitamin D-binding protein	137	-	-	-	-	NP
Q15118	[Pyruvate dehydrogenase (acetyl-transferring)] kinase isozyme 1. mitochondrial	72	-	-	-	-	NWP
P01011	Alpha-1-antichymotrypsin	68	-	-	-	-	NWP
P04920	Anion exchange protein 2	45	-	-	-	-	NWP
Q9H115	Beta-soluble NSF attachment protein	84	-	-	-	-	NWP
P27482	Calmodulin-like protein 3	417	-	-	-	-	NWP
P23528	Cofilin-1	269	-	-	-	-	NWP
Q02487	Desmocollin-2	38	-	-	-	-	NWP
Q92616	eIF-2-alpha kinase activator GCN1	57	-	-	-	-	NWP
Q9GZZ8	Extracellular glycoprotein lacritin	2060	-	-	-	-	NWP
Q01469	Fatty acid-binding protein 5	557	-	-	-	-	NWP
Q5W0V3	FHF complex subunit HOOK interacting protein 2A	159	-	-	-	-	NWP
Q08380	Galectin-3-binding protein	92	-	-	-	-	NWP
P18510	Interleukin-1 receptor antagonist protein	113	-	-	-	-	NWP
O95274	Ly6/PLAUR domain-containing protein 3	118	-	-	-	-	NWP
Q96DR8	Mucin-like protein 1	606	-	-	-	-	NWP
Q15406	Nuclear receptor subfamily 6 group A member 1	48	-	-	-	-	NWP
Q9Y536	Peptidyl-prolyl cis-trans isomerase A-like 4A	54	-	-	-	-	NWP
P07602	Prosaposin	116	-	-	-	-	NWP
A8MUU1	Putative fatty acid-binding protein 5-like protein 3	79	-	-	-	-	NWP
Q53EL9	Seizure protein 6 homolog	70	-	-	-	-	NWP
P29508	Serpin B3	82	-	-	-	-	NWP
P48594	Serpin B4	82	-	-	-	-	NWP
Q96RM1	Small proline-rich protein 2F	75	-	-	-	-	NWP
Q8WXA9	Splicing regulatory glutamine/lysine-rich protein 1	63	-	-	-	-	NWP
P20061	Transcobalamin-1	201	-	-	-	-	NWP
P60174	Triosephosphate isomerase	181	-	-	-	-	NWP
Q8IXR9	Uncharacterized protein C12orf56	54	-	-	-	-	NWP
P11684	Uteroglobin	3363	-	-	-	-	NWP
P25311	Zinc-alpha-2-glycoprotein	290	-	-	-	-	NWP
P15516	Histatin-3	958	1.84	0.61	0.77	0.49	SE
P09211	Glutathione S-transferase P	1006	1.40	0.34	0.22	0.94	SE
P01615	Immunoglobulin kappa variable 2D-28	236	1.35	0.30	0.27	0.87	SE
P01614	Immunoglobulin kappa variable 2D-40	236	1.34	0.29	0.20	0.90	SE
P01009	Alpha-1-antitrypsin	95	1.34	0.29	0.26	0.82	SE
A0A087WW87	Immunoglobulin kappa variable 2-40	236	1.34	0.29	0.24	0.84	SE
A0A075B6S2	Immunoglobulin kappa variable 2D-29	236	1.30	0.26	0.24	0.82	SE
A2NJV5	Immunoglobulin kappa variable 2-29	236	1.28	0.25	0.22	0.85	SE
A0A075B6S6	Immunoglobulin kappa variable 2D-30	236	1.28	0.25	0.20	0.82	SE
A0A075B6P5	Immunoglobulin kappa variable 2-28	236	1.27	0.24	0.28	0.83	SE

P06310	Immunoglobulin kappa variable 2-30	236	1.27	0.24	0.28	0.83	SE
P52566	Rho GDP-dissociation inhibitor 2	370	1.27	0.24	0.15	0.94	SE
Q9Y6R7	IgGFc-binding protein	45	1.26	0.23	0.21	0.81	SE
A0A0A0MRZ7	Immunoglobulin kappa variable 2D-26	236	1.26	0.23	0.26	0.78	SE
P62937	Peptidyl-prolyl cis-trans isomerase A	226	1.26	0.23	0.17	0.94	SE
P00739	Haptoglobin-related protein	43	1.23	0.21	0.42	0.66	SE
P54108	Cysteine-rich secretory protein 3	885	1.22	0.20	0.13	0.89	SE
Q01518	Adenylyl cyclase-associated protein 1	135	1.16	0.15	0.21	0.66	SE
P07205	Phosphoglycerate kinase 2	83	1.16	0.15	0.54	0.65	SE
A0M8Q6	Immunoglobulin lambda constant 7	2152	1.08	0.08	0.12	0.76	SE
P04406	Glyceraldehyde-3-phosphate dehydrogenase	705	1.04	0.04	0.13	0.64	SE
P17066	Heat shock 70 kDa protein 6	95	1.03	0.03	0.34	0.53	SE
Q14508	WAP four-disulfide core domain protein 2	2229	1.00	0	0.18	0.51	SE
P00558	Phosphoglycerate kinase 1	83	0.98	-0.02	0.25	0.49	SE
P11142	Heat shock cognate 71 kDa protein	82	0.96	-0.04	0.24	0.42	SE
P61626	Lysozyme C	3743	0.95	-0.05	0.04	0.17	SE
Q9UBC9	Small proline-rich protein 3	1968	0.94	-0.06	0.07	0.23	SE
P59665	Neutrophil defensin 1	112	0.91	-0.09	0.09	0.22	SE
P59666	Neutrophil defensin 3	112	0.91	-0.09	0.08	0.18	SE
P11021	Endoplasmic reticulum chaperone BiP	101	0.90	-0.10	0.31	0.37	SE
P54652	Heat shock-related 70 kDa protein 2	101	0.88	-0.13	0.35	0.39	SE
P48741	Putative heat shock 70 kDa protein 7	95	0.88	-0.13	0.37	0.35	SE
P52209	6-phosphogluconate dehydrogenase, decarboxylating	144	0.84	-0.17	0.09	0.07	SE
P06396	Gelsolin	47	0.80	-0.22	0.29	0.26	SE
P15515	Histatin-1	17313	0.43	-0.84	0.66	0.27	SE
P02808	Statherin	46710	0.26	-1.33	0.38	0.06	SE

Note: Ratio NP/NWP (fold change) = ratio between pregnant women with normal BMI but with periodontitis and control group proteins (pregnant women with normal BMI and without periodontitis); Log(e) (“e” is a constant = 2.71); SD, standard deviation; *p*, statistical significance (adjusted by False Discovery Rate–FDR = 4); ↑ = up-regulated (1-p > 0.95); ↓ = down-regulated (p < 0.05); SE = similar expression compared to control group; bold lines refer to up- or down-regulated proteins by more than 2-fold

S1–Table C. Proteins identified in saliva of OP and NP during T1 and their differences in expression

Accession number	Protein name	Score	Ratio OP/NP	Log(e)	SD	*p*	Expression differences
**P69905**	**Hemoglobin subunit alpha**	**711**	**36.23**	**3.59**	**0.01**	**< 0.01**	↑
**P02100**	**Hemoglobin subunit epsilon**	**271**	**20.91**	**3.04**	**0.02**	**< 0.01**	↑
**P69891**	**Hemoglobin subunit gamma-1**	**271**	**20.09**	**3.00**	**0.02**	**< 0.01**	↑
**P69892**	**Hemoglobin subunit gamma-2**	**271**	**19.30**	**2.96**	**0.02**	**0.01**	↑
**P00739**	**Haptoglobin-related protein**	**16**	**9.87**	**2.29**	**0.08**	**< 0.01**	↑
**P00738**	**Haptoglobin**	**114**	**9.03**	**2.20**	**0.05**	**< 0.01**	↑
**Q14651**	**Plastin-1**	**33**	**3.82**	**1.34**	**0.23**	**< 0.01**	↑

**P02787**	**Serotransferrin**	**118**	**3.29**	**1.19**	**0.08**	**< 0.01**	↑
**P04080**	**Cystatin-B**	**1239**	**3.06**	**1.12**	**0.07**	**< 0.01**	↑
**P01009**	**Alpha-1-antitrypsin**	**180**	**2.92**	**1.07**	**0.08**	**< 0.01**	↑
**P26038**	**Moesin**	**50**	**2.89**	**1.06**	**0.11**	**< 0.01**	↑
**Q02817**	**Mucin-2**	**20**	**2.83**	**1.04**	**0.12**	**< 0.01**	↑
**P35325**	**Small proline-rich protein 2B**	**245**	**2.64**	**0.97**	**0.23**	**< 0.01**	↑
**P22531**	**Small proline-rich protein 2E**	**245**	**2.61**	**0.96**	**0.22**	**< 0.01**	↑
**P22532**	**Small proline-rich protein 2D**	**245**	**2.59**	**0.95**	**0.20**	**0.01**	↑
**Q9BYE4**	**Small proline-rich protein 2G**	**245**	**2.53**	**0.93**	**0.20**	**0.01**	↑
**P48741**	**Putative heat shock 70 kDa protein 7**	**52**	**2.51**	**0.92**	**0.20**	**< 0.01**	↑
**P02679**	**Fibrinogen gamma chain**	**118**	**2.48**	**0.91**	**0.10**	**< 0.01**	↑
**P62937**	**Peptidyl-prolyl cis-trans isomerase A**	**357**	**2.48**	**0.91**	**0.12**	**< 0.01**	↑
**P61769**	**Beta-2-microglobulin**	**638**	**2.44**	**0.89**	**0.18**	**< 0.01**	↑
**P17066**	**Heat shock 70 kDa protein 6**	**52**	**2.41**	**0.88**	**0.15**	**< 0.01**	↑
**P01591**	**Immunoglobulin J chain**	**697**	**2.39**	**0.87**	**0.04**	**< 0.01**	↑
**P07864**	**L-lactate dehydrogenase C chain**	**56**	**2.36**	**0.86**	**0.20**	**< 0.01**	↑
**P01833**	**Polymeric immunoglobulin receptor**	**810**	**2.29**	**0.83**	**0.02**	**< 0.01**	↑
**P54652**	**Heat shock-related 70 kDa protein 2**	**58**	**2.27**	**0.82**	**0.16**	**< 0.01**	↑
**P02814**	**Submaxillary gland androgen-regulated protein 3B**	**1091**	**2.25**	**0.81**	**0.02**	**< 0.01**	↑
**P02675**	**Fibrinogen beta chain**	**511**	**2.18**	**0.78**	**0.11**	**< 0.01**	↑
**P07195**	**L-lactate dehydrogenase B chain**	**56**	**2.18**	**0.78**	**0.23**	**0.02**	↑
**Q6ZMR3**	**L-lactate dehydrogenase A-like 6A**	**56**	**2.16**	**0.77**	**0.17**	**< 0.01**	↑
**P02042**	**Hemoglobin subunit delta**	**499**	**2.14**	**0.76**	**0.04**	**< 0.01**	↑
**P04746**	**Pancreatic alpha-amylase**	**2426**	**2.12**	**0.75**	**0.01**	**< 0.01**	↑
**Q8NHQ9**	**ATP-dependent RNA helicase DDX55**	**169**	**2.10**	**0.74**	**0.24**	**< 0.01**	↑
**P0DMV9**	**Heat shock 70 kDa protein 1B**	**184**	**2.08**	**0.73**	**0.09**	**< 0.01**	↑
**P11142**	**Heat shock cognate 71 kDa protein**	**67**	**2.08**	**0.73**	**0.14**	**< 0.01**	↑
**P0DUB6**	**Alpha-amylase 1A**	**3994**	**2.05**	**0.72**	**0.01**	**< 0.01**	↑
**P35326**	**Small proline-rich protein 2A**	**526**	**2.05**	**0.72**	**0.13**	**< 0.01**	↑
**P34931**	**Heat shock 70 kDa protein 1-like**	**181**	**2.01**	**0.70**	**0.09**	**< 0.01**	↑
**P11021**	**Endoplasmic reticulum chaperone BiP**	**30**	**2.00**	**0.69**	**0.19**	**< 0.01**	↑
**P0DMV8**	**Heat shock 70 kDa protein 1A**	**196**	**2.00**	**0.69**	**0.08**	**< 0.01**	↑
P01877	Immunoglobulin heavy constant alpha 2	496	1.93	0.66	0.05	< 0.01	↑
P80188	Neutrophil gelatinase-associated lipocalin	366	1.90	0.64	0.07	< 0.01	↑
P02812	Basic salivary proline-rich protein 2	366	1.88	0.63	0.11	< 0.01	↑
P52209	6-phosphogluconate dehydrogenase, decarboxylating	73	1.82	0.60	0.08	< 0.01	↑
P0DOX2	Immunoglobulin alpha-2 heavy chain	449	1.82	0.60	0.02	< 0.01	↑
P31025	Lipocalin-1	1539	1.82	0.60	0.11	< 0.01	↑
P20742	Pregnancy zone protein	212	1.82	0.60	0.09	< 0.01	↑
P30613	Pyruvate kinase PKLR	129	1.79	0.58	0.14	< 0.01	↑
P01876	Immunoglobulin heavy constant alpha 1	1300	1.77	0.57	0.01	< 0.01	↑
P0DOX7	Immunoglobulin kappa light chain	2520	1.73	0.55	0.05	< 0.01	↑
P22079	Lactoperoxidase	82	1.65	0.50	0.07	< 0.01	↑

P01857	Immunoglobulin heavy constant gamma 1	324	1.57	0.45	0.04	< 0.01	↑
P0DOX5	Immunoglobulin gamma-1 heavy chain	324	1.55	0.44	0.03	< 0.01	↑
P19961	Alpha-amylase 2B	3339	1.54	0.43	0.03	< 0.01	↑
P02774	Vitamin D-binding protein	137	1.51	0.41	0.17	< 0.01	↑
P09104	Gamma-enolase	32	1.49	0.40	0.11	< 0.01	↑
P0DOX6	Immunoglobulin mu heavy chain	254	1.48	0.39	0.07	< 0.01	↑
P01859	Immunoglobulin heavy constant gamma 2	85	1.45	0.37	0.11	< 0.01	↑
P01871	Immunoglobulin heavy constant mu	288	1.45	0.37	0.06	< 0.01	↑
A0M8Q6	Immunoglobulin lambda constant 7	320	1.45	0.37	0.11	< 0.01	↑
P0DTE8	Alpha-amylase 1C	3994	1.42	0.35	0.02	< 0.01	↑
P06702	Protein S100-A9	344	1.39	0.33	0.06	< 0.01	↑
P0DTE7	Alpha-amylase 1B	3994	1.38	0.32	0.01	< 0.01	↑
P13929	Beta-enolase	50	1.38	0.32	0.15	0.03	↑
P37837	Transaldolase	152	1.38	0.32	0.11	< 0.01	↑
Q5VSP4	Putative lipocalin 1-like protein 1	967	1.36	0.31	0.08	< 0.01	↑
P29401	Transketolase	171	1.34	0.29	0.12	< 0.01	↑
P14780	Matrix metalloproteinase-9	82	1.30	0.26	0.11	0.01	↑
P01860	Immunoglobulin heavy constant gamma 3	85	1.28	0.25	0.05	< 0.01	↑
P01861	Immunoglobulin heavy constant gamma 4	85	1.27	0.24	0.06	< 0.01	↑
P02810	Salivary acidic proline-rich phosphoprotein 1/2	2308	1.27	0.24	0.04	< 0.01	↑
P02647	Apolipoprotein A-I	4432	1.23	0.21	0.07	< 0.01	↑
P00338	L-lactate dehydrogenase A chain	117	1.22	0.20	0.10	0.04	↑
P05109	Protein S100-A8	452	1.07	0.07	0.03	0.02	↑
P01037	Cystatin-SN	3510	0.84	-0.18	0.03	< 0.01	↓
P06733	Alpha-enolase	236	0.74	-0.30	0.04	< 0.01	↓
P01036	Cystatin-S	6686	0.73	-0.32	0.05	< 0.01	↓
P02788	Lactotransferrin	58	0.73	-0.32	0.06	< 0.01	↓
P63261	Actin, cytoplasmic 2	1384	0.71	-0.34	0.04	< 0.01	↓
Q9UBG3	Cornulin	258	0.69	-0.37	0.12	< 0.01	↓
P02768	Albumin	2047	0.65	-0.43	0.01	< 0.01	↓
Q6S8J3	POTE ankyrin domain family member E	366	0.64	-0.44	0.06	< 0.01	↓
P60709	Actin, cytoplasmic 1	1384	0.63	-0.47	0.03	< 0.01	↓
A5A3E0	POTE ankyrin domain family member F	366	0.61	-0.49	0.05	< 0.01	↓
P01023	Alpha-2-macroglobulin	545	0.61	-0.50	0.09	< 0.01	↓
P68133	Actin, alpha skeletal muscle	904	0.55	-0.59	0.03	< 0.01	↓
Q6P5S2	Protein LEG1 homolog	404	0.54	-0.61	0.22	< 0.01	↓
Q9BYX7	Putative beta-actin-like protein 3	109	0.54	-0.62	0.04	< 0.01	↓
P59666	Neutrophil defensin 3	1062	0.53	-0.64	0.07	< 0.01	↓
P62736	Actin, aortic smooth muscle	904	0.52	-0.65	0.05	< 0.01	↓
P59665	Neutrophil defensin 1	1062	0.52	-0.65	0.08	< 0.01	↓
P61626	Lysozyme C	3223	0.51	-0.67	0.06	< 0.01	↓
P68032	Actin, alpha cardiac muscle 1	904	0.51	-0.68	0.05	< 0.01	↓
Q562R1	Beta-actin-like protein 2	362	0.51	-0.68	0.09	< 0.01	↓
**P23280**	**Carbonic anhydrase 6**	**1067**	**0.49**	**-0.71**	**0.17**	**< 0.01**	↓

**P01834**	**Immunoglobulin kappa constant**	**3138**	**0.46**	**-0.78**	**0.04**	**< 0.01**	↓
**Q8NBJ4**	**Golgi membrane protein 1**	**63**	**0.44**	**-0.81**	**0.27**	**0.02**	↓
**Q9H299**	**SH3 domain-binding glutamic acid-rich-like protein 3**	**354**	**0.44**	**-0.83**	**0.26**	**< 0.01**	↓
**P63267**	**Actin, gamma-enteric smooth muscle**	**904**	**0.43**	**-0.84**	**0.05**	**< 0.01**	↓
**Q96DA0**	**Zymogen granule protein 16 homolog B**	**1097**	**0.35**	**-1.04**	**0.03**	**< 0.01**	↓
**Q16378**	**Proline-rich protein 4**	**984**	**0.31**	**-1.17**	**0.08**	**0.01**	↓
**P09228**	**Cystatin-SA**	**808**	**0.26**	**-1.34**	**0.02**	**< 0.01**	↓
**Q8TAX7**	**Mucin-7**	**842**	**0.23**	**-1.48**	**0.04**	**< 0.01**	↓
**P12273**	**Prolactin-inducible protein**	**1165**	**0.19**	**-1.64**	**0.02**	**< 0.01**	↓
**P28325**	**Cystatin-D**	**352**	**0.18**	**-1.74**	**0.07**	**< 0.01**	↓
P31947	14-3-3 protein sigma	448	-	-	-	-	OP
Q6P587	Acylpyruvase FAHD1, mitochondrial	568	-	-	-	-	OP
P02763	Alpha-1-acid glycoprotein 1	280	-	-	-	-	OP
P02765	Alpha-2-HS-glycoprotein	427	-	-	-	-	OP
P12814	Alpha-actinin-1	62	-	-	-	-	OP
O43707	Alpha-actinin-4	50	-	-	-	-	OP
P01019	Angiotensinogen	180	-	-	-	-	OP
Q96LR9	Apolipoprotein L domain-containing protein 1	70	-	-	-	-	OP
P27482	Calmodulin-like protein 3	227	-	-	-	-	OP
P00450	Ceruloplasmin	590	-	-	-	-	OP
P23528	Cofilin-1	895	-	-	-	-	OP
Q92616	eIF-2-alpha kinase activator GCN1	45	-	-	-	-	OP
Q9P2K8	eIF-2-alpha kinase GCN2	306	-	-	-	-	OP
Q01469	Fatty acid-binding protein 5	769	-	-	-	-	OP
Q5W0V3	FHF complex subunit HOOK interacting protein 2A	152	-	-	-	-	OP
Q08380	Galectin-3-binding protein	99	-	-	-	-	OP
P06737	Glycogen phosphorylase, liver form	45	-	-	-	-	OP
Q8WYH8	Inhibitor of growth protein 5	568	-	-	-	-	OP
P18510	Interleukin-1 receptor antagonist protein	112	-	-	-	-	OP
P06870	Kallikrein-1	95	-	-	-	-	OP
P01033	Metalloproteinase inhibitor 1	175	-	-	-	-	OP
Q8NCY6	Myb/SANT-like DNA-binding domain-containing protein 4	165	-	-	-	-	OP
P80303	Nucleobindin-2	79	-	-	-	-	OP
Q8NGQ2	Olfactory receptor 6Q1	201	-	-	-	-	OP
Q9Y536	Peptidyl-prolyl cis-trans isomerase A-like 4A	130	-	-	-	-	OP
Q8N6L0	Protein KASH5	62	-	-	-	-	OP
Q9Y5F8	Protocadherin gamma-B7	47	-	-	-	-	OP
P50120	Retinol-binding protein 2	390	-	-	-	-	OP
Q96RM1	Small proline-rich protein 2F	243	-	-	-	-	OP
Q14515	SPARC-like protein 1	31	-	-	-	-	OP
P20061	Transcobalamin-1	81	-	-	-	-	OP
P49770	Translation initiation factor eIF-2B subunit beta	36	-	-	-	-	OP

P02766	Transthyretin	452	-	-	-	-	OP
P60174	Triosephosphate isomerase	137	-	-	-	-	OP
P36537	UDP-glucuronosyltransferase 2B10	23	-	-	-	-	OP
Q9BY64	UDP-glucuronosyltransferase 2B28	23	-	-	-	-	OP
Q9UJU3	Zinc finger protein 112	47	-	-	-	-	OP
P25311	Zinc-alpha-2-glycoprotein	210	-	-	-	-	OP
P27216	Annexin A13	392	-	-	-	-	NP
Q8TDL5	BPI fold-containing family B member 1	86	-	-	-	-	NP
P0DP23	Calmodulin-1	252	-	-	-	-	NP
P0DP24	Calmodulin-2	252	-	-	-	-	NP
P0DP25	Calmodulin-3	252	-	-	-	-	NP
Q8NEL0	Coiled-coil domain-containing protein 54	185	-	-	-	-	NP
Q99543	DnaJ homolog subfamily C member 2	51	-	-	-	-	NP
P15311	Ezrin	20	-	-	-	-	NP
Q9P2Q2	FERM domain-containing protein 4A	24	-	-	-	-	NP
Q5RHP9	Glutamate-rich protein 3	41	-	-	-	-	NP
O14556	Glyceraldehyde-3-phosphate dehydrogenase, testis-specific	93	-	-	-	-	NP
P02008	Hemoglobin subunit zeta	710	-	-	-	-	NP
A0A075B6P5	Immunoglobulin kappa variable 2-28	146	-	-	-	-	NP
A2NJV5	Immunoglobulin kappa variable 2-29	146	-	-	-	-	NP
P06310	Immunoglobulin kappa variable 2-30	146	-	-	-	-	NP
A0A087WW87	Immunoglobulin kappa variable 2-40	146	-	-	-	-	NP
A0A0A0MRZ7	Immunoglobulin kappa variable 2D-26	146	-	-	-	-	NP
P01615	Immunoglobulin kappa variable 2D-28	160	-	-	-	-	NP
A0A075B6S2	Immunoglobulin kappa variable 2D-29	146	-	-	-	-	NP
A0A075B6S6	Immunoglobulin kappa variable 2D-30	146	-	-	-	-	NP
P01614	Immunoglobulin kappa variable 2D-40	146	-	-	-	-	NP
Q6B0I6	Lysine-specific demethylase 4D	104	-	-	-	-	NP
P47874	Olfactory marker protein	102	-	-	-	-	NP
Q9Y2S7	Polymerase delta-interacting protein 2	70	-	-	-	-	NP
Q9UQ80	Proliferation-associated protein 2G4	41	-	-	-	-	NP
Q6MZM9	Proline-rich protein 27	248	-	-	-	-	NP
P80511	Protein S100-A12	209	-	-	-	-	NP
P35241	Radixin	19	-	-	-	-	NP
Q6UWP8	Suprabasin	60	-	-	-	-	NP
P26639	Threonine--tRNA ligase 1, cytoplasmic	92	-	-	-	-	NP
Q9Y4F4	TOG array regulator of axonemal microtubules protein 1	97	-	-	-	-	NP
P51809	Vesicle-associated membrane protein 7	100	-	-	-	-	NP
P02808	Statherin	2286	1.60	0.47	0.57	0.47	SE
P04280	Basic salivary proline-rich protein 1	366	1.57	0.45	0.39	0.85	SE
P01767	Immunoglobulin heavy variable 3-53	928	1.46	0.38	0.36	0.82	SE
P01772	Immunoglobulin heavy variable 3-33	928	1.46	0.38	0.35	0.86	SE
A0A0B4J1X5	Immunoglobulin heavy variable 3-74	928	1.43	0.36	0.42	0.77	SE

P0DP03	Immunoglobulin heavy variable 3-30-5	928	1.43	0.36	0.39	0.79	SE
P01764	Immunoglobulin heavy variable 3-23	928	1.36	0.31	0.43	0.80	SE
P01768	Immunoglobulin heavy variable 3-30	928	1.36	0.31	0.37	0.78	SE
P0DP02	Immunoglobulin heavy variable 3-30-3	928	1.28	0.25	0.37	0.74	SE
Q8N4F0	BPI fold-containing family B member 2	422	1.25	0.22	0.15	0.91	SE
A0A0C4DH42	Immunoglobulin heavy variable 3-66	928	1.22	0.20	0.45	0.74	SE
Q9UJ14	Glutathione hydrolase 7	64	1.22	0.20	0.15	0.86	SE
Q14508	WAP four-disulfide core domain protein 2	108	1.17	0.16	0.22	0.76	SE
P01034	Cystatin-C	141	1.15	0.14	0.12	0.82	SE
P54108	Cysteine-rich secretory protein 3	74	1.14	0.13	0.22	0.70	SE
P03973	Antileukoproteinase	1387	1.11	0.10	0.17	0.71	SE
P07108	Acyl-CoA-binding protein	195	1.08	0.08	0.20	0.65	SE
P04075	Fructose-bisphosphate aldolase A	194	1.06	0.06	0.15	0.71	SE
P0CG38	POTE ankyrin domain family member I	257	0.99	-0.01	0.10	0.53	SE
P02790	Hemopexin	153	0.98	-0.02	0.24	0.38	SE
P04406	Glyceraldehyde-3-phosphate dehydrogenase	1223	0.98	-0.02	0.12	0.39	SE
P04433	Immunoglobulin kappa variable 3-11	578	0.98	-0.02	0.51	0.57	SE
Q96DR5	BPI fold-containing family A member 2	221	0.98	-0.02	0.07	0.40	SE
A8K2U0	Alpha-2-macroglobulin-like protein 1	38	0.97	-0.03	0.15	0.38	SE
P24158	Myeloblastin	68	0.94	-0.06	0.25	0.46	SE
Q9UBC9	Small proline-rich protein 3	3688	0.93	-0.07	0.19	0.44	SE
P13797	Plastin-3	86	0.92	-0.08	0.56	0.55	SE
P13796	Plastin-2	322	0.91	-0.09	0.08	0.15	SE
A0A0A0MRZ8	Immunoglobulin kappa variable 3D-11	578	0.90	-0.10	0.51	0.51	SE
P07737	Profilin-1	869	0.90	-0.10	0.06	0.05	SE
P09211	Glutathione S-transferase P	247	0.90	-0.10	0.13	0.23	SE
P10599	Thioredoxin	143	0.89	-0.12	0.16	0.18	SE
Q01518	Adenylyl cyclase-associated protein 1	130	0.85	-0.16	0.12	0.10	SE
P0CG39	POTE ankyrin domain family member J	176	0.84	-0.18	0.11	0.07	SE
P15515	Histatin-1	202	0.41	-0.88	0.36	0.08	SE
P15516	Histatin-3	1825	0.41	-0.90	0.58	0.50	SE

Note: Ratio OP/NP (fold change) = ratio between pregnant women with obesity and periodontitis and control group proteins (pregnant women with normal BMI but with periodontitis); Log(e) ("e" is a constant = 2.71); SD, standard deviation; *p*, statistical significance (adjusted by False Discovery Rate–FDR = 4); ↑ = up-regulated (1-p > 0.95); ↓ = down-regulated (p < 0.05); SE = similar expression compared to control group; bold lines refer to up- or down-regulated proteins by more than 2-fold

S1–Table D. Proteins identified in saliva of OWP and NWP during T1 and their differences in expression

Accession number	Protein name	Score	Ratio OWP/NWP	Log(e)	SD	*p*	Expression differences
**Q14508**	**WAP four-disulfide core domain protein 2**	**2229**	**15.49**	**2.74**	**0.04**	**< 0.01**	↑
**P02100**	**Hemoglobin subunit epsilon**	**119**	**9.03**	**2.20**	**0.08**	**0.02**	↑
**P69891**	**Hemoglobin subunit gamma-1**	**119**	**9.03**	**2.20**	**0.08**	**0.03**	↑

**P69892**	**Hemoglobin subunit gamma-2**	**119**	**8.94**	**2.19**	**0.09**	**0.02**	↑
**P24158**	**Myeloblastin**	**177**	**8.25**	**2.11**	**0.10**	**< 0.01**	↑
**P02042**	**Hemoglobin subunit delta**	**151**	**7.54**	**2.02**	**0.07**	**< 0.01**	↑
**P01024**	**Complement C3**	**45**	**7.24**	**1.98**	**0.12**	**< 0.01**	↑
**P00739**	**Haptoglobin-related protein**	**43**	**6.89**	**1.93**	**0.19**	**< 0.01**	↑
**P02787**	**Serotransferrin**	**391**	**6.75**	**1.91**	**0.02**	**< 0.01**	↑
**P01861**	**Immunoglobulin heavy constant gamma 4**	**122**	**6.30**	**1.84**	**0.07**	**< 0.01**	↑
**P52209**	**6-phosphogluconate dehydrogenase, decarboxylating**	**144**	**5.05**	**1.62**	**0.05**	**< 0.01**	↑
**P01859**	**Immunoglobulin heavy constant gamma 2**	**30**	**5.05**	**1.62**	**0.07**	**< 0.01**	↑
**P01009**	**Alpha-1-antitrypsin**	**95**	**4.53**	**1.51**	**0.10**	**< 0.01**	↑
**P04080**	**Cystatin-B**	**5550**	**4.53**	**1.51**	**0.04**	**< 0.01**	↑
**P14618**	**Pyruvate kinase PKM**	**135**	**4.44**	**1.49**	**0.09**	**< 0.01**	↑
**P00738**	**Haptoglobin**	**132**	**4.31**	**1.46**	**0.10**	**< 0.01**	↑
**P07737**	**Profilin-1**	**859**	**4.31**	**1.46**	**0.09**	**< 0.01**	↑
**P01023**	**Alpha-2-macroglobulin**	**27**	**4.18**	**1.43**	**0.11**	**< 0.01**	↑
**P01860**	**Immunoglobulin heavy constant gamma 3**	**103**	**4.06**	**1.40**	**0.08**	**< 0.01**	↑
**P04406**	**Glyceraldehyde-3-phosphate dehydrogenase**	**705**	**3.97**	**1.38**	**0.10**	**< 0.01**	↑
**P37837**	**Transaldolase**	**199**	**3.71**	**1.31**	**0.09**	**< 0.01**	↑
**P23528**	**Cofilin-1**	**269**	**3.60**	**1.28**	**0.29**	**< 0.01**	↑
**Q02487**	**Desmocollin-2**	**38**	**3.25**	**1.18**	**0.30**	**< 0.01**	↑
**P04075**	**Fructose-bisphosphate aldolase A**	**182**	**2.77**	**1.02**	**0.12**	**< 0.01**	↑
**P07205**	**Phosphoglycerate kinase 2**	**83**	**2.77**	**1.02**	**0.35**	**0.03**	↑
**Q9H299**	**SH3 domain-binding glutamic acid-rich-like protein 3**	**305**	**2.77**	**1.02**	**0.34**	**< 0.01**	↑
**P06744**	**Glucose-6-phosphate isomerase**	**96**	**2.72**	**1.00**	**0.17**	**< 0.01**	↑
**Q6S8J3**	**POTE ankyrin domain family member E**	**337**	**2.64**	**0.97**	**0.03**	**< 0.01**	↑
**P68871**	**Hemoglobin subunit beta**	**151**	**2.48**	**0.91**	**0.11**	**< 0.01**	↑
**P0CG38**	**POTE ankyrin domain family member I**	**83**	**2.41**	**0.88**	**0.06**	**< 0.01**	↑
**P07602**	**Prosaposin**	**116**	**2.41**	**0.88**	**0.23**	**< 0.01**	↑
**P68032**	**Actin, alpha cardiac muscle 1**	**884**	**2.32**	**0.84**	**0.04**	**< 0.01**	↑
**P63267**	**Actin, gamma-enteric smooth muscle**	**884**	**2.32**	**0.84**	**0.04**	**< 0.01**	↑
**P62736**	**Actin, aortic smooth muscle**	**884**	**2.27**	**0.82**	**0.03**	**< 0.01**	↑
**P34931**	**Heat shock 70 kDa protein 1-like**	**90**	**2.25**	**0.81**	**0.14**	**< 0.01**	↑
**A5A3E0**	**POTE ankyrin domain family member F**	**337**	**2.25**	**0.81**	**0.05**	**< 0.01**	↑
**P59665**	**Neutrophil defensin 1**	**112**	**2.18**	**0.78**	**0.07**	**< 0.01**	↑
**P59666**	**Neutrophil defensin 3**	**112**	**2.12**	**0.75**	**0.07**	**< 0.01**	↑
**P68133**	**Actin, alpha skeletal muscle**	**884**	**2.03**	**0.71**	**0.06**	**< 0.01**	↑
Q9BYX7	Putative beta-actin-like protein 3	254	1.93	0.66	0.09	< 0.01	↑
P0DMV8	Heat shock 70 kDa protein 1A	116	1.90	0.64	0.12	< 0.01	↑
A0M8Q6	Immunoglobulin lambda constant 7	2152	1.88	0.63	0.10	< 0.01	↑
P0DMV9	Heat shock 70 kDa protein 1B	116	1.86	0.62	0.15	< 0.01	↑
P29401	Transketolase	89	1.84	0.61	0.18	< 0.01	↑
P0CG39	POTE ankyrin domain family member J	83	1.80	0.59	0.10	< 0.01	↑
P09211	Glutathione S-transferase P	1006	1.79	0.58	0.20	0.01	↑

P06396	Gelsolin	47	1.77	0.57	0.15	< 0.01	↑
P09104	Gamma-enolase	124	1.65	0.50	0.17	0.01	↑
P06702	Protein S100-A9	181	1.58	0.46	0.05	< 0.01	↑
P01857	Immunoglobulin heavy constant gamma 1	184	1.57	0.45	0.05	< 0.01	↑
P20061	Transcobalamin-1	201	1.49	0.40	0.17	0.01	↑
P0CF74	Immunoglobulin lambda constant 6	3630	1.48	0.39	0.07	< 0.01	↑
P61626	Lysozyme C	3743	1.48	0.39	0.09	< 0.01	↑
P13796	Plastin-2	123	1.45	0.37	0.11	< 0.01	↑
P02790	Hemopexin	108	1.43	0.36	0.15	0.01	↑
P0DOY3	Immunoglobulin lambda constant 3	4884	1.43	0.36	0.10	< 0.01	↑
P0DOX5	Immunoglobulin gamma-1 heavy chain	184	1.42	0.35	0.08	< 0.01	↑
P02788	Lactotransferrin	425	1.40	0.34	0.05	< 0.01	↑
P0CG04	Immunoglobulin lambda constant 1	3086	1.35	0.3	0.08	< 0.01	↑
P01591	Immunoglobulin J chain	9177	1.19	0.17	0.05	< 0.01	↑
P01833	Polymeric immunoglobulin receptor	16349	1.17	0.16	0.02	< 0.01	↑
Q96DR5	BPI fold-containing family A member 2	944	1.16	0.15	0.06	< 0.01	↑
P02768	Albumin	6357	1.07	0.07	0.02	< 0.01	↑
P01876	Immunoglobulin heavy constant alpha 1	9432	1.07	0.07	0.01	< 0.01	↑
P01877	Immunoglobulin heavy constant alpha 2	4178	1.05	0.05	0.02	0.02	↑
P09228	Cystatin-SA	3237	0.84	-0.18	0.04	< 0.01	↓
P01037	Cystatin-SN	10770	0.82	-0.20	0.02	< 0.01	↓
P06310	Immunoglobulin kappa variable 2-30	236	0.76	-0.28	0.21	0.04	↓
P54108	Cysteine-rich secretory protein 3	885	0.72	-0.33	0.14	0.01	↓
P01871	Immunoglobulin heavy constant mu	118	0.71	-0.34	0.12	< 0.01	↓
Q96DA0	Zymogen granule protein 16 homolog B	22215	0.71	-0.34	0.03	< 0.01	↓
Q8N4F0	BPI fold-containing family B member 2	612	0.66	-0.42	0.08	< 0.01	↓
P60709	Actin_ cytoplasmic 1	1017	0.65	-0.43	0.04	< 0.01	↓
P63261	Actin_ cytoplasmic 2	1014	0.63	-0.46	0.04	< 0.01	↓
P22079	Lactoperoxidase	617	0.58	-0.54	0.07	< 0.01	↓
P28325	Cystatin-D	333	0.57	-0.56	0.07	< 0.01	↓
Q9UBC9	Small proline-rich protein 3	1968	0.55	-0.59	0.10	< 0.01	↓
P0DTE7	Alpha-amylase 1B	41555	0.53	-0.63	0.01	< 0.01	↓
P04746	Pancreatic alpha-amylase	28592	0.53	-0.64	0.01	< 0.01	↓
P0DTE8	Alpha-amylase 1C	41555	0.52	-0.65	0.01	< 0.01	↓
P12273	Prolactin-inducible protein	25257	0.51	-0.67	0.02	< 0.01	↓
**A8K2U0**	**Alpha-2-macroglobulin-like protein 1**	**109**	**0.47**	**-0.75**	**0.29**	**0.01**	↓
**P10599**	**Thioredoxin**	**4451**	**0.47**	**-0.75**	**0.13**	**< 0.01**	↓
**P0DUB6**	**Alpha-amylase 1A**	**41555**	**0.45**	**-0.79**	**0.01**	**< 0.01**	↓
**P01034**	**Cystatin-C**	**3385**	**0.44**	**-0.81**	**0.09**	**< 0.01**	↓
**P02647**	**Apolipoprotein A-I**	**171**	**0.44**	**-0.83**	**0.14**	**< 0.01**	↓
**P19961**	**Alpha-amylase 2B**	**35774**	**0.43**	**-0.85**	**0.01**	**< 0.01**	↓
**P15516**	**Histatin-3**	**958**	**0.40**	**-0.92**	**0.20**	**< 0.01**	↓
**P01036**	**Cystatin-S**	**16480**	**0.36**	**-1.01**	**0.03**	**< 0.01**	↓
**P03973**	**Antileukoproteinase**	**1419**	**0.27**	**-1.30**	**0.13**	**< 0.01**	↓
**Q16378**	**Proline-rich protein 4**	**3936**	**0.21**	**-1.56**	**0.10**	**< 0.01**	↓

**P23280**	**Carbonic anhydrase 6**	**1177**	**0.16**	**-1.81**	**0.02**	**< 0.01**	↓
**Q6P5S2**	**Protein LEG1 homolog**	**2046**	**0.16**	**-1.86**	**0.09**	**< 0.01**	↓
**Q8TAX7**	**Mucin-7**	**1497**	**0.14**	**-2.00**	**0.03**	**< 0.01**	↓
**P02810**	**Salivary acidic proline-rich phosphoprotein 1/2**	**3931**	**0.10**	**-2.31**	**0.01**	**< 0.01**	↓
**Q8TDL5**	**BPI fold-containing family B member 1**	**912**	**0.10**	**-2.35**	**0.06**	**< 0.01**	↓
**P02808**	**Statherin**	**46710**	**0.02**	**-4.09**	**0.05**	**< 0.01**	↓
P02763	Alpha-1-acid glycoprotein 1	756	-	-	-	-	OWP
P02765	Alpha-2-HS-glycoprotein	579	-	-	-	-	OWP
P01019	Angiotensinogen	193	-	-	-	-	OWP
Q5SW79	Centrosomal protein of 170 kDa	90	-	-	-	-	OWP
O15078	Centrosomal protein of 290 kDa	162	-	-	-	-	OWP
P00450	Ceruloplasmin	882	-	-	-	-	OWP
P10909	Clusterin	191	-	-	-	-	OWP
Q9Y281	Cofilin-2	139	-	-	-	-	OWP
A2RUR9	Coiled-coil domain-containing protein 144A	52	-	-	-	-	OWP
Q3MJ40	Coiled-coil domain-containing protein 144B	45	-	-	-	-	OWP
P0C0L4	Complement C4-A	72	-	-	-	-	OWP
P0C0L5	Complement C4-B	70	-	-	-	-	OWP
Q9UBG3	Cornulin	100	-	-	-	-	OWP
P02671	Fibrinogen alpha chain	360	-	-	-	-	OWP
P02675	Fibrinogen beta chain	2032	-	-	-	-	OWP
P02679	Fibrinogen gamma chain	2273	-	-	-	-	OWP
Q92993	Histone acetyltransferase KAT5	157	-	-	-	-	OWP
P06870	Kallikrein-1	107	-	-	-	-	OWP
O14782	Kinesin-like protein KIF3C	52	-	-	-	-	OWP
P00338	L-lactate dehydrogenase A chain	877	-	-	-	-	OWP
Q6ZMR3	L-lactate dehydrogenase A-like 6A	351	-	-	-	-	OWP
P07195	L-lactate dehydrogenase B chain	325	-	-	-	-	OWP
P07864	L-lactate dehydrogenase C chain	325	-	-	-	-	OWP
O60449	Lymphocyte antigen 75	18	-	-	-	-	OWP
P40925	Malate dehydrogenase, cytoplasmic	117	-	-	-	-	OWP
O75556	Mammaglobin-B	522	-	-	-	-	OWP
P14780	Matrix metalloproteinase-9	179	-	-	-	-	OWP
O00255	Menin	47	-	-	-	-	OWP
Q8NEM0	Microcephalin	50	-	-	-	-	OWP
O43318	Mitogen-activated protein kinase kinase kinase 7	42	-	-	-	-	OWP
P26038	Moesin	110	-	-	-	-	OWP
P05164	Myeloperoxidase	87	-	-	-	-	OWP
O75161	Nephrocystin-4	62	-	-	-	-	OWP
P80188	Neutrophil gelatinase-associated lipocalin	2719	-	-	-	-	OWP
P30041	Peroxiredoxin-6	153	-	-	-	-	OWP
Q8TBY8	Polyamine-modulated factor 1-binding protein 1	45	-	-	-	-	OWP
P20742	Pregnancy zone protein	25	-	-	-	-	OWP
P02760	Protein AMBP	149	-	-	-	-	OWP
P07237	Protein disulfide-isomerase	180	-	-	-	-	OWP

Q6NUI1	Putative coiled-coil domain-containing protein 144 N-terminal-like	39	-	-	-	-	OWP
Q8IYA2	Putative coiled-coil domain-containing protein 144C	50	-	-	-	-	OWP
P35241	Radixin	88	-	-	-	-	OWP
P35249	Replication factor C subunit 4	66	-	-	-	-	OWP
Q8N392	Rho GTPase-activating protein 18	59	-	-	-	-	OWP
Q96QB1	Rho GTPase-activating protein 7	85	-	-	-	-	OWP
Q9NTJ3	Structural maintenance of chromosomes protein 4	46	-	-	-	-	OWP
Q6PKC3	Thioredoxin domain-containing protein 11	17	-	-	-	-	OWP
Q9Y4F4	TOG array regulator of axonemal microtubules protein 1	52	-	-	-	-	OWP
P02766	Transthyretin	255	-	-	-	-	OWP
Q9BXT4	Tudor domain-containing protein 1	19	-	-	-	-	OWP
P36941	Tumor necrosis factor receptor superfamily member 3	87	-	-	-	-	OWP
O94966	Ubiquitin carboxyl-terminal hydrolase 19	20	-	-	-	-	OWP
P02774	Vitamin D-binding protein	1144	-	-	-	-	OWP
P04004	Vitronectin	65	-	-	-	-	OWP
Q96KN7	X-linked retinitis pigmentosa GTPase regulator-interacting protein 1	61	-	-	-	-	OWP
Q63HK3	Zinc finger protein with KRAB and SCAN domains 2	43	-	-	-	-	OWP
Q15118	[Pyruvate dehydrogenase (acetyl-transferring)] kinase isozyme 1, mitochondrial	72	-	-	-	-	NWP
P01011	Alpha-1-antichymotrypsin	68	-	-	-	-	NWP
P04920	Anion exchange protein 2	45	-	-	-	-	NWP
Q9H115	Beta-soluble NSF attachment protein	84	-	-	-	-	NWP
P27482	Calmodulin-like protein 3	417	-	-	-	-	NWP
Q92616	eIF-2-alpha kinase activator GCN1	57	-	-	-	-	NWP
Q9GZZ8	Extracellular glycoprotein lacritin	2060	-	-	-	-	NWP
Q5W0V3	FHF complex subunit HOOK interacting protein 2A	159	-	-	-	-	NWP
Q08380	Galectin-3-binding protein	92	-	-	-	-	NWP
Q9UJ14	Glutathione hydrolase 7	123	-	-	-	-	NWP
P15515	Histatin-1	17313	-	-	-	-	NWP
Q9Y6R7	IgGFc-binding protein	45	-	-	-	-	NWP
Q96DR8	Mucin-like protein 1	606	-	-	-	-	NWP
Q15406	Nuclear receptor subfamily 6 group A member 1	48	-	-	-	-	NWP
A8MUU1	Putative fatty acid-binding protein 5-like protein 3	79	-	-	-	-	NWP
Q53EL9	Seizure protein 6 homolog	70	-	-	-	-	NWP
P29508	Serpin B3	82	-	-	-	-	NWP
P48594	Serpin B4	82	-	-	-	-	NWP
P35326	Small proline-rich protein 2A	733	-	-	-	-	NWP
P35325	Small proline-rich protein 2B	373	-	-	-	-	NWP
P22532	Small proline-rich protein 2D	373	-	-	-	-	NWP
P22531	Small proline-rich protein 2E	373	-	-	-	-	NWP

Q96RM1	Small proline-rich protein 2F	75	-	-	-	-	NWP
Q9BYE4	Small proline-rich protein 2G	347	-	-	-	-	NWP
Q8WXA9	Splicing regulatory glutamine/lysine-rich protein 1	63	-	-	-	-	NWP
Q8IXR9	Uncharacterized protein C12orf56	54	-	-	-	-	NWP
P11684	Uteroglobin	3363	-	-	-	-	NWP
P30613	Pyruvate kinase PKLR	91	2.14	0.76	0.48	0.89	SE
P62937	Peptidyl-prolyl cis-trans isomerase A	226	1.42	0.35	0.22	0.91	SE
P60174	Triosephosphate isomerase	181	1.36	0.31	0.26	0.81	SE
P00558	Phosphoglycerate kinase 1	83	1.34	0.29	0.22	0.87	SE
P0DOY2	Immunoglobulin lambda constant 2	4884	1.32	0.28	0.14	0.93	SE
P0DOX6	Immunoglobulin mu heavy chain	118	1.31	0.27	0.20	0.87	SE
O95274	Ly6/PLAUR domain-containing protein 3	118	1.21	0.19	0.33	0.72	SE
P61769	Beta-2-microglobulin	132	1.20	0.18	0.12	0.92	SE
P11142	Heat shock cognate 71 kDa protein	82	1.20	0.18	0.23	0.73	SE
P05109	Protein S100-A8	250	1.19	0.17	0.11	0.91	SE
P0DOX8	Immunoglobulin lambda-1 light chain	3256	1.17	0.16	0.15	0.78	SE
P0DOX7	Immunoglobulin kappa light chain	276	1.16	0.15	0.10	0.92	SE
B9A064	Immunoglobulin lambda-like polypeptide 5	3303	1.14	0.13	0.09	0.95	SE
P17066	Heat shock 70 kDa protein 6	95	1.09	0.09	0.36	0.50	SE
Q5VSP4	Putative lipocalin 1-like protein 1	4592	1.08	0.08	0.05	0.91	SE
P25311	Zinc-alpha-2-glycoprotein	290	1.08	0.08	0.23	0.52	SE
P48741	Putative heat shock 70 kDa protein 7	95	1.06	0.06	0.38	0.57	SE
P31025	Lipocalin-1	5807	1.05	0.05	0.05	0.86	SE
P54652	Heat shock-related 70 kDa protein 2	101	1.04	0.04	0.37	0.54	SE
Q01518	Adenylyl cyclase-associated protein 1	135	1.03	0.03	0.26	0.47	SE
P11021	Endoplasmic reticulum chaperone BiP	101	1.03	0.03	0.40	0.53	SE
P02814	Submaxillary gland androgen-regulated protein 3B	51408	1.03	0.03	0.05	0.54	SE
P0DOX2	Immunoglobulin alpha-2 heavy chain	4092	1.02	0.02	0.01	0.87	SE
P18510	Interleukin-1 receptor antagonist protein	113	1.01	0.01	0.26	0.49	SE
Q9Y536	Peptidyl-prolyl cis-trans isomerase A-like 4A	54	1.01	0.01	0.52	0.53	SE
Q562R1	Beta-actin-like protein 2	626	0.99	-0.01	0.06	0.44	SE
P01834	Immunoglobulin kappa constant	1907	0.96	-0.04	0.06	0.23	SE
P52566	Rho GDP-dissociation inhibitor 2	370	0.95	-0.05	0.19	0.45	SE
Q01469	Fatty acid-binding protein 5	557	0.89	-0.12	0.21	0.32	SE
Q9UGM3	Deleted in malignant brain tumors 1 protein	393	0.83	-0.19	0.14	0.08	SE
A2NJV5	Immunoglobulin kappa variable 2-29	236	0.82	-0.20	0.17	0.17	SE
P01615	Immunoglobulin kappa variable 2D-28	236	0.82	-0.20	0.25	0.15	SE
P69905	Hemoglobin subunit alpha	95	0.79	-0.23	0.17	0.11	SE
A0A075B6P5	Immunoglobulin kappa variable 2-28	236	0.79	-0.24	0.20	0.14	SE
A0A087WW87	Immunoglobulin kappa variable 2-40	236	0.77	-0.26	0.20	0.13	SE
A0A0A0MRZ7	Immunoglobulin kappa variable 2D-26	236	0.76	-0.28	0.21	0.06	SE
A0A075B6S2	Immunoglobulin kappa variable 2D-29	236	0.76	-0.28	0.14	0.06	SE
P01614	Immunoglobulin kappa variable 2D-40	236	0.76	-0.28	0.20	0.08	SE

A0A075B6S6	Immunoglobulin kappa variable 2D-30	236	0.74	-0.30	0.18	0.05	SE
P07108	Acyl-CoA-binding protein	297	0.66	-0.41	0.21	0.06	SE

Note: Ratio OWP/NWP (fold change) = ratio between pregnant women with obesity but without periodontitis and control group proteins (pregnant women with normal BMI and without periodontitis); Log(e) ("e" is a constant = 2.71); SD, standard deviation; *p*, statistical significance (adjusted by False Discovery Rate–FDR = 4); ↑ = up-regulated (1-p > 0.95); ↓ = down-regulated (p < 0.05); SE = similar expression compared to control group; bold lines refer to up- or down-regulated proteins by more than 2-fold
