# Supplementary material for: Label-Free Quantitative Proteomic Analysis Reveals Inflammatory Pattern Associated with Obesity and Periodontitis in Pregnant Women
Source: Metabolites. 2022 Nov 10;12(11):1091. doi: 10.3390/metabo12111091 (PMC9692340; doi:10.3390/metabo12111091)
Supplement: Supplementary file 1 [file metabolites-12-01091-s001.zip › Supplementary file S2.pdf]

S2–Table A. Proteins identified in saliva of OP and OWP during T2 and their differences in expression

Accession number	Protein name	Score	Ratio OP/OWP	Log(e)	SD	*p*	Expression differences
**P02042**	**Hemoglobin subunit delta**	**1039**	**19.11**	**2.95**	**0.09**	**< 0.01**	↑
**P68871**	**Hemoglobin subunit beta**	**4166**	**18.73**	**2.93**	**0.01**	**< 0.01**	↑
**P69905**	**Hemoglobin subunit alpha**	**1983**	**8.50**	**2.14**	**0.02**	**< 0.01**	↑
**P02810**	**Salivary acidic proline-rich phosphoprotein 1/2**	**659**	**6.89**	**1.93**	**0.03**	**< 0.01**	↑
**P02814**	**Submaxillary gland androgen-regulated protein 3B**	**2912**	**6.89**	**1.93**	**0.01**	**< 0.01**	↑
**P02808**	**Statherin**	**2512**	**5.05**	**1.62**	**0.06**	**< 0.01**	↑
**P0CG39**	**POTE ankyrin domain family member J**	**37**	**4.76**	**1.56**	**0.03**	**< 0.01**	↑
**P14618**	**Pyruvate kinase PKM**	**213**	**4.14**	**1.42**	**0.10**	**< 0.01**	↑
**P07737**	**Profilin-1**	**1975**	**3.97**	**1.38**	**0.05**	**< 0.01**	↑
**P01860**	**Immunoglobulin heavy constant gamma 3**	**559**	**2.44**	**0.89**	**0.07**	**< 0.01**	↑
**P01857**	**Immunoglobulin heavy constant gamma 1**	**2147**	**2.23**	**0.80**	**0.04**	**< 0.01**	↑
**Q9UBG3**	**Cornulin**	**48**	**2.18**	**0.78**	**0.28**	**< 0.01**	↑
**A0M8Q6**	**Immunoglobulin lambda constant 7**	**1011**	**2.08**	**0.73**	**0.07**	**< 0.01**	↑
**A8K2U0**	**Alpha-2-macroglobulin-like protein 1**	**33**	**2.05**	**0.72**	**0.26**	**0.01**	↑
**P34931**	**Heat shock 70 kDa protein 1-like**	**146**	**2.01**	**0.70**	**0.22**	**0.01**	↑
**P0DMV8**	**Heat shock 70 kDa protein 1A**	**151**	**2.01**	**0.70**	**0.20**	**< 0.01**	↑
P0DMV9	Heat shock 70 kDa protein 1B	150	1.95	0.67	0.18	0.01	↑
P0CF74	Immunoglobulin lambda constant 6	2283	1.79	0.58	0.07	< 0.01	↑
P27482	Calmodulin-like protein 3	121	1.77	0.57	0.16	0.01	↑
P68032	Actin, alpha cardiac muscle 1	180	1.65	0.50	0.06	< 0.01	↑
P61626	Lysozyme C	150	1.63	0.49	0.06	< 0.01	↑
P60709	Actin, cytoplasmic 1	277	1.55	0.44	0.08	< 0.01	↑
P63267	Actin, gamma-enteric smooth muscle	180	1.52	0.42	0.08	< 0.01	↑
P62736	Actin, aortic smooth muscle	180	1.51	0.41	0.09	< 0.01	↑
P0DOX5	Immunoglobulin gamma-1 heavy chain	2147	1.46	0.38	0.11	< 0.01	↑
P63261	Actin, cytoplasmic 2	272	1.36	0.31	0.04	< 0.01	↑
Q6S8J3	POTE ankyrin domain family member E	62	1.36	0.31	0.07	< 0.01	↑
P68133	Actin, alpha skeletal muscle	180	1.35	0.30	0.05	< 0.01	↑
A5A3E0	POTE ankyrin domain family member F	62	1.35	0.30	0.05	< 0.01	↑
Q562R1	Beta-actin-like protein 2	143	1.32	0.28	0.05	< 0.01	↑
Q9UGM3	Deleted in malignant brain tumors 1 protein	148	1.21	0.19	0.07	0.02	↑
P19961	Alpha-amylase 2B	7498	1.12	0.11	0.01	< 0.01	↑
P01037	Cystatin-SN	2007	1.09	0.09	0.02	< 0.01	↑
Q96DA0	Zymogen granule protein 16 homolog B	1714	1.08	0.08	0.03	< 0.01	↑
P04746	Pancreatic alpha-amylase	6383	1.05	0.05	0.01	< 0.01	↑
P0DUB6	Alpha-amylase 1A	8980	1.04	0.04	0.01	< 0.01	↑
P0DTE7	Alpha-amylase 1B	8980	1.04	0.04	0.01	< 0.01	↑
P0DTE8	Alpha-amylase 1C	8980	1.04	0.04	0.01	< 0.01	↑
P0DOX2	Immunoglobulin alpha-2 heavy chain	429	0.90	-0.11	0.02	< 0.01	↓

P02768	Albumin	5626	0.87	-0.14	0.02	< 0.01	↓
Q5VSP4	Putative lipocalin 1-like protein 1	1296	0.84	-0.17	0.07	0.01	↓
P22079	Lactoperoxidase	28	0.80	-0.22	0.11	0.02	↓
Q9BYX7	Putative beta-actin-like protein 3	1669	0.80	-0.22	0.05	< 0.01	↓
P01871	Immunoglobulin heavy constant mu	36	0.79	-0.24	0.09	< 0.01	↓
P02679	Fibrinogen gamma chain	189	0.78	-0.25	0.10	0.03	↓
P01833	Polymeric immunoglobulin receptor	2106	0.77	-0.26	0.06	< 0.01	↓
P0DOX6	Immunoglobulin mu heavy chain	36	0.76	-0.27	0.10	< 0.01	↓
P31025	Lipocalin-1	2310	0.76	-0.27	0.05	< 0.01	↓
P05109	Protein S100-A8	8350	0.67	-0.40	0.07	< 0.01	↓
P0DOY3	Immunoglobulin lambda constant 3	340	0.64	-0.44	0.09	< 0.01	↓
P01034	Cystatin-C	147	0.63	-0.46	0.08	< 0.01	↓
P02788	Lactotransferrin	148	0.63	-0.46	0.07	< 0.01	↓
P23280	Carbonic anhydrase 6	83	0.61	-0.49	0.06	< 0.01	↓
Q8N4F0	BPI fold-containing family B member 2	59	0.58	-0.54	0.08	< 0.01	↓
P13929	Beta-enolase	55	0.58	-0.55	0.15	< 0.01	↓
P01591	Immunoglobulin J chain	419	0.53	-0.63	0.05	< 0.01	↓
P62937	Peptidyl-prolyl cis-trans isomerase A	138	0.51	-0.68	0.26	0.03	↓
**P59666**	**Neutrophil defensin 3**	**271**	**0.47**	**-0.75**	**0.20**	**0.01**	↓
**P01834**	**Immunoglobulin kappa constant**	**3629**	**0.47**	**-0.76**	**0.05**	**< 0.01**	↓
**P02812**	**Basic salivary proline-rich protein 2**	**699**	**0.45**	**-0.79**	**0.07**	**< 0.01**	↓
**P0DOX7**	**Immunoglobulin kappa light chain**	**1094**	**0.45**	**-0.79**	**0.03**	**< 0.01**	↓
**P01036**	**Cystatin-S**	**1382**	**0.42**	**-0.86**	**0.02**	**< 0.01**	↓
**P09228**	**Cystatin-SA**	**500**	**0.32**	**-1.13**	**0.03**	**< 0.01**	↓
**P12273**	**Prolactin-inducible protein**	**2680**	**0.29**	**-1.23**	**0.02**	**< 0.01**	↓
**P10599**	**Thioredoxin**	**152**	**0.27**	**-1.32**	**0.16**	**< 0.01**	↓
**P06702**	**Protein S100-A9**	**2638**	**0.24**	**-1.43**	**0.05**	**< 0.01**	↓
**Q8TAX7**	**Mucin-7**	**62**	**0.19**	**-1.67**	**0.03**	**< 0.01**	↓
**P04080**	**Cystatin-B**	**2200**	**0.17**	**-1.76**	**0.03**	**< 0.01**	↓
**P69892**	**Hemoglobin subunit gamma-2**	**932**	**0.17**	**-1.78**	**0.13**	**< 0.01**	↓
**P69891**	**Hemoglobin subunit gamma-1**	**932**	**0.17**	**-1.79**	**0.14**	**< 0.01**	↓
**P02100**	**Hemoglobin subunit epsilon**	**932**	**0.16**	**-1.82**	**0.13**	**< 0.01**	↓
**P28325**	**Cystatin-D**	**540**	**0.16**	**-1.83**	**0.09**	**< 0.01**	↓
P52209	6-phosphogluconate dehydrogenase, decarboxylating	200	-	-	-	-	OP
P0DP23	Calmodulin-1	281	-	-	-	-	OP
P0DP24	Calmodulin-2	281	-	-	-	-	OP
P0DP25	Calmodulin-3	281	-	-	-	-	OP
Q8N126	Cell adhesion molecule 3	61	-	-	-	-	OP
O95196	Chondroitin sulfate proteoglycan 5	86	-	-	-	-	OP
P23528	Cofilin-1	295	-	-	-	-	OP
Q8N998	Coiled-coil domain-containing protein 89	34	-	-	-	-	OP
P54108	Cysteine-rich secretory protein 3	61	-	-	-	-	OP
Q14181	DNA polymerase alpha subunit B	43	-	-	-	-	OP
P49792	E3 SUMO-protein ligase RanBP2	13	-	-	-	-	OP

Q5W0V3	FHF complex subunit HOOK interacting protein 2A	199	-	-	-	-	OP
P04075	Fructose-bisphosphate aldolase A	69	-	-	-	-	OP
P06744	Glucose-6-phosphate isomerase	68	-	-	-	-	OP
Q9BX51	Glutathione hydrolase light chain 1	129	-	-	-	-	OP
Q86Z02	Homeodomain-interacting protein kinase 1	26	-	-	-	-	OP
O76013	Keratin, type I cuticular Ha6	82	-	-	-	-	OP
P00338	L-lactate dehydrogenase A chain	81	-	-	-	-	OP
Q8IZ02	Leucine-rich repeat-containing protein 34	20	-	-	-	-	OP
P26038	Moesin	68	-	-	-	-	OP
O15105	Mothers against decapentaplegic homolog 7	60	-	-	-	-	OP
P80188	Neutrophil gelatinase-associated lipocalin	635	-	-	-	-	OP
P62942	Peptidyl-prolyl cis-trans isomerase FKBP1A	238	-	-	-	-	OP
Q96BP3	Peptidylprolyl isomerase domain and WD repeat-containing protein 1	21	-	-	-	-	OP
P55201	Peregrin	37	-	-	-	-	OP
P00558	Phosphoglycerate kinase 1	123	-	-	-	-	OP
P52566	Rho GDP-dissociation inhibitor 2	488	-	-	-	-	OP
Q99986	Serine/threonine-protein kinase VRK1	87	-	-	-	-	OP
P29508	Serpin B3	199	-	-	-	-	OP
P48594	Serpin B4	181	-	-	-	-	OP
Q9H299	SH3 domain-binding glutamic acid-rich-like protein 3	2120	-	-	-	-	OP
Q9Y4F4	TOG array regulator of axonemal microtubules protein 1	27	-	-	-	-	OP
P02766	Transthyretin	211	-	-	-	-	OP
P07108	Acyl-CoA-binding protein	235	-	-	-	-	OWP
P02763	Alpha-1-acid glycoprotein 1	116	-	-	-	-	OWP
Q8TDL5	BPI fold-containing family B member 1	93	-	-	-	-	OWP
Q5SW79	Centrosomal protein of 170 kDa	150	-	-	-	-	OWP
P33991	DNA replication licensing factor MCM4	19	-	-	-	-	OWP
P32519	ETS-related transcription factor Elf-1	27	-	-	-	-	OWP
Q08380	Galectin-3-binding protein	15	-	-	-	-	OWP
P06396	Gelsolin	68	-	-	-	-	OWP
P17066	Heat shock 70 kDa protein 6	87	-	-	-	-	OWP
Q969F9	Hermansky-Pudlak syndrome 3 protein	29	-	-	-	-	OWP
P15515	Histatin-1	2908	-	-	-	-	OWP
Q9NR48	Histone-lysine N-methyltransferase ASH1L	17	-	-	-	-	OWP
Q2TBA0	Kelch-like protein 40	41	-	-	-	-	OWP
Q9BUT9	MAPK regulated corepressor interacting protein 2	42	-	-	-	-	OWP
Q02817	Mucin-2	33	-	-	-	-	OWP
A8MUU1	Putative fatty acid-binding protein 5-like protein 3	227	-	-	-	-	OWP
P48741	Putative heat shock 70 kDa protein 7	87	-	-	-	-	OWP
P30613	Pyruvate kinase PKLR	65	-	-	-	-	OWP

Q9NSD5	Sodium- and chloride-dependent GABA transporter 2	141	-	-	-	-	OWP
O60264	SWI/SNF-related matrix-associated actin-dependent regulator of chromatin subfamily A member 5	14	-	-	-	-	OWP
Q5JTD0	Tight junction-associated protein 1	19	-	-	-	-	OWP
Q6ZVM7	TOM1-like protein 2	66	-	-	-	-	OWP
Q9BXT4	Tudor domain-containing protein 1	33	-	-	-	-	OWP
Q502W6	von Willebrand factor A domain-containing protein 3B	65	-	-	-	-	OWP
P11142	Heat shock cognate 71 kDa protein	87	1.62	0.48	0.32	0.87	SE
Q9UBC9	Small proline-rich protein 3	394	1.49	0.40	0.19	0.95	SE
P11021	Endoplasmic reticulum chaperone BiP	57	1.45	0.37	0.30	0.86	SE
P37837	Transaldolase	102	1.40	0.34	0.27	0.88	SE
P54652	Heat shock-related 70 kDa protein 2	87	1.38	0.32	0.29	0.82	SE
P59665	Neutrophil defensin 1	271	1.27	0.24	0.17	0.95	SE
P61769	Beta-2-microglobulin	654	1.26	0.23	0.55	0.49	SE
P02647	Apolipoprotein A-I	286	1.19	0.17	0.11	0.94	SE
P01861	Immunoglobulin heavy constant gamma 4	125	1.19	0.17	0.26	0.60	SE
Q6P5S2	Protein LEG1 homolog	297	1.15	0.14	0.14	0.84	SE
A0A075B6S6	Immunoglobulin kappa variable 2D-30	231	1.07	0.07	0.17	0.60	SE
Q96DR5	BPI fold-containing family A member 2	193	1.06	0.06	0.06	0.85	SE
P02790	Hemopexin	235	1.06	0.06	0.12	0.68	SE
Q01518	Adenylyl cyclase-associated protein 1	208	1.04	0.04	0.24	0.54	SE
Q01469	Fatty acid-binding protein 5	582	1.04	0.04	0.14	0.61	SE
P06310	Immunoglobulin kappa variable 2-30	231	1.03	0.03	0.16	0.55	SE
P01876	Immunoglobulin heavy constant alpha 1	1507	1.02	0.02	0.01	0.95	SE
A0A0A0MRZ7	Immunoglobulin kappa variable 2D-26	231	1.02	0.02	0.18	0.55	SE
P01024	Complement C3	49	1.01	0.01	0.19	0.51	SE
P09104	Gamma-enolase	55	1.01	0.01	0.12	0.49	SE
P01877	Immunoglobulin heavy constant alpha 2	490	1.01	0.01	0.29	0.81	SE
A0A075B6P5	Immunoglobulin kappa variable 2-28	231	1.01	0.01	0.19	0.55	SE
A2NJV5	Immunoglobulin kappa variable 2-29	231	1.01	0.01	0.18	0.57	SE
A0A075B6S2	Immunoglobulin kappa variable 2D-29	231	1.01	0.01	0.18	0.49	SE
P01615	Immunoglobulin kappa variable 2D-28	231	1.00	0	0.15	0.54	SE
A0A087WW87	Immunoglobulin kappa variable 2-40	231	0.99	-0.01	0.19	0.50	SE
P01614	Immunoglobulin kappa variable 2D-40	231	0.99	-0.01	0.17	0.49	SE
P0CG04	Immunoglobulin lambda constant 1	164	0.99	-0.01	0.07	0.34	SE
P06733	Alpha-enolase	317	0.97	-0.03	0.10	0.39	SE
P0DOX8	Immunoglobulin lambda-1 light chain	164	0.97	-0.03	0.05	0.35	SE
P0CG38	POTE ankyrin domain family member I	37	0.97	-0.03	0.11	0.38	SE
P02675	Fibrinogen beta chain	160	0.96	-0.04	0.16	0.43	SE
P0DOY2	Immunoglobulin lambda constant 2	340	0.96	-0.04	0.04	0.26	SE
B9A064	Immunoglobulin lambda-like polypeptide 5	164	0.96	-0.04	0.04	0.23	SE
Q8NHQ9	ATP-dependent RNA helicase DDX55	906	0.92	-0.08	0.67	0.55	SE
P00739	Haptoglobin-related protein	86	0.92	-0.08	0.14	0.34	SE

P02787	Serotransferrin	433	0.92	-0.08	0.04	0.05	SE
P25311	Zinc-alpha-2-glycoprotein	95	0.91	-0.09	0.20	0.32	SE
P09211	Glutathione S-transferase P	85	0.90	-0.11	0.44	0.30	SE
P00738	Haptoglobin	699	0.87	-0.14	0.08	0.07	SE
P01023	Alpha-2-macroglobulin	50	0.86	-0.15	0.09	0.07	SE
P01859	Immunoglobulin heavy constant gamma 2	98	0.86	-0.15	0.10	0.06	SE
P15516	Histatin-3	3202	0.83	-0.19	0.18	0.16	SE
P04406	Glyceraldehyde-3-phosphate dehydrogenase	235	0.81	-0.21	0.14	0.12	SE
Q14508	WAP four-disulfide core domain protein 2	531	0.76	-0.27	0.15	0.05	SE
P13796	Plastin-2	176	0.76	-0.28	0.17	0.06	SE
P20742	Pregnancy zone protein	91	0.75	-0.29	0.29	0.14	SE
P04280	Basic salivary proline-rich protein 1	511	0.14	-1.99	0.24	0.05	SE

Note: Ratio OP/OWP (fold change) = ratio between women with obesity and periodontitis and control group proteins (women with obesity but without periodontitis); Log(e) ("e" is a constant = 2.71); SD, standard deviation; *p*, statistical significance (adjusted by False Discovery Rate–FDR = 4); ↑ = up-regulated (1-p > 0.95); ↓ = down-regulated (p < 0.05); SE = similar expression compared to control group; bold lines refer to up- or down-regulated proteins by more than 2-fold

S2–Table B. Proteins identified in saliva of NP and NWP during T2 and their differences in expression

Accession number	Protein name	Score	Ratio NP/NWP	Log(e)	SD	*p*	Expression differences
**P01009**	**Alpha-1-antitrypsin**	**171**	**6.82**	**1.92**	**0.05**	**< 0.01**	↑
**P02647**	**Apolipoprotein A-I**	**905**	**5.99**	**1.79**	**0.03**	**< 0.01**	↑
**P02042**	**Hemoglobin subunit delta**	**2300**	**5.37**	**1.68**	**0.04**	**< 0.01**	↑
**P68871**	**Hemoglobin subunit beta**	**1059**	**4.22**	**1.44**	**0.05**	**< 0.01**	↑
**P59666**	**Neutrophil defensin 3**	**887**	**4.14**	**1.42**	**0.06**	**< 0.01**	↑
**P59665**	**Neutrophil defensin 1**	**887**	**4.10**	**1.41**	**0.04**	**< 0.01**	↑
**P00739**	**Haptoglobin-related protein**	**68**	**3.60**	**1.28**	**0.19**	**< 0.01**	↑
**P04080**	**Cystatin-B**	**4062**	**3.53**	**1.26**	**0.05**	**< 0.01**	↑
**P61626**	**Lysozyme C**	**484**	**3.53**	**1.26**	**0.04**	**< 0.01**	↑
**P69905**	**Hemoglobin subunit alpha**	**339**	**3.35**	**1.21**	**0.12**	**< 0.01**	↑
**P02768**	**Albumin**	**15244**	**3.13**	**1.14**	**0.01**	**< 0.01**	↑
**Q8NHQ9**	**ATP-dependent RNA helicase DDX55**	**235**	**3.10**	**1.13**	**0.12**	**< 0.01**	↑
**P01023**	**Alpha-2-macroglobulin**	**91**	**2.94**	**1.08**	**0.04**	**< 0.01**	↑
**P00738**	**Haptoglobin**	**192**	**2.86**	**1.05**	**0.06**	**< 0.01**	↑
**P61769**	**Beta-2-microglobulin**	**784**	**2.77**	**1.02**	**0.06**	**< 0.01**	↑
**P10599**	**Thioredoxin**	**480**	**2.64**	**0.97**	**0.24**	**0.02**	↑
**P68133**	**Actin, alpha skeletal muscle**	**5916**	**2.56**	**0.94**	**0.06**	**< 0.01**	↑
**P22079**	**Lactoperoxidase**	**68**	**2.36**	**0.86**	**0.13**	**< 0.01**	↑
**P01871**	**Immunoglobulin heavy constant mu**	**148**	**2.25**	**0.81**	**0.09**	**< 0.01**	↑
**Q562R1**	**Beta-actin-like protein 2**	**4453**	**2.20**	**0.79**	**0.05**	**< 0.01**	↑
**P62736**	**Actin, aortic smooth muscle**	**5916**	**2.14**	**0.76**	**0.05**	**< 0.01**	↑
**P0DOX6**	**Immunoglobulin mu heavy chain**	**148**	**2.01**	**0.70**	**0.12**	**< 0.01**	↑

**P01591**	**Immunoglobulin J chain**	**4092**	**2.00**	**0.69**	**0.05**	**< 0.01**	↑
Q8TAX7	Mucin-7	1302	1.93	0.66	0.05	< 0.01	↑
P28325	Cystatin-D	1595	1.88	0.63	0.06	< 0.01	↑
P20742	Pregnancy zone protein	66	1.86	0.62	0.31	0.04	↑
P25311	Zinc-alpha-2-glycoprotein	80	1.84	0.61	0.25	0.02	↑
P0CF74	Immunoglobulin lambda constant 6	946	1.80	0.59	0.04	< 0.01	↑
P0DOY2	Immunoglobulin lambda constant 2	1261	1.79	0.58	0.04	< 0.01	↑
P0CG04	Immunoglobulin lambda constant 1	1469	1.77	0.57	0.04	< 0.01	↑
P0DOY3	Immunoglobulin lambda constant 3	1261	1.77	0.57	0.04	< 0.01	↑
P0DOX8	Immunoglobulin lambda-1 light chain	1469	1.77	0.57	0.04	< 0.01	↑
B9A064	Immunoglobulin lambda-like polypeptide 5	1469	1.77	0.57	0.04	< 0.01	↑
Q96DA0	Zymogen granule protein 16 homolog B	6943	1.72	0.54	0.02	< 0.01	↑
P02790	Hemopexin	468	1.67	0.51	0.10	< 0.01	↑
P01876	Immunoglobulin heavy constant alpha 1	7226	1.67	0.51	0.01	< 0.01	↑
P05109	Protein S100-A8	110	1.67	0.51	0.10	< 0.01	↑
P01860	Immunoglobulin heavy constant gamma 3	377	1.60	0.47	0.09	< 0.01	↑
P01859	Immunoglobulin heavy constant gamma 2	228	1.54	0.43	0.07	< 0.01	↑
P01861	Immunoglobulin heavy constant gamma 4	224	1.54	0.43	0.06	< 0.01	↑
P02787	Serotransferrin	703	1.52	0.42	0.04	< 0.01	↑
P0DOX5	Immunoglobulin gamma-1 heavy chain	3271	1.49	0.40	0.04	< 0.01	↑
P29401	Transketolase	115	1.49	0.40	0.16	0.01	↑
P01857	Immunoglobulin heavy constant gamma 1	3271	1.43	0.36	0.03	< 0.01	↑
Q8N4F0	BPI fold-containing family B member 2	343	1.38	0.32	0.06	< 0.01	↑
P0DOX7	Immunoglobulin kappa light chain	317	1.15	0.14	0.05	0.01	↑
P01834	Immunoglobulin kappa constant	2112	1.11	0.10	0.04	< 0.01	↑
P68032	Actin, alpha cardiac muscle 1	5916	0.90	-0.10	0.06	0.03	↓
P63261	Actin, cytoplasmic 2	7700	0.90	-0.11	0.04	< 0.01	↓
Q9UGM3	Deleted in malignant brain tumors 1 protein	153	0.82	-0.20	0.09	0.04	↓
O95196	Chondroitin sulfate proteoglycan 5	36	0.74	-0.30	0.06	0.01	↓
P02810	Salivary acidic proline-rich phosphoprotein 1/2	2359	0.71	-0.34	0.03	< 0.01	↓
P01877	Immunoglobulin heavy constant alpha 2	3603	0.70	-0.36	0.02	< 0.01	↓
P0DOX2	Immunoglobulin alpha-2 heavy chain	3309	0.69	-0.37	0.02	< 0.01	↓
P0CG39	POTE ankyrin domain family member J	457	0.66	-0.42	0.14	0.03	↓
P09228	Cystatin-SA	6385	0.64	-0.45	0.04	< 0.01	↓
P52209	6-phosphogluconate dehydrogenase, decarboxylating	81	0.62	-0.48	0.14	< 0.01	↓
P12273	Prolactin-inducible protein	13097	0.57	-0.56	0.02	< 0.01	↓
P0CG38	POTE ankyrin domain family member I	559	0.57	-0.57	0.15	< 0.01	↓
A0M8Q6	Immunoglobulin lambda constant 7	199	0.55	-0.60	0.25	0.04	↓
P00338	L-lactate dehydrogenase A chain	246	0.53	-0.63	0.24	< 0.01	↓
Q01518	Adenylyl cyclase-associated protein 1	464	0.52	-0.66	0.24	< 0.01	↓
P01036	Cystatin-S	15763	0.52	-0.66	0.06	< 0.01	↓
P37837	Transaldolase	133	0.52	-0.66	0.13	< 0.01	↓
**Q5VSP4**	**Putative lipocalin 1-like protein 1**	**1864**	**0.47**	**-0.75**	**0.05**	**< 0.01**	↓

**P0DTE8**	**Alpha-amylase 1C**	**21876**	**0.46**	**-0.77**	**0.03**	**< 0.01**	↓
**P31025**	**Lipocalin-1**	**3326**	**0.46**	**-0.78**	**0.04**	**< 0.01**	↓
**P19961**	**Alpha-amylase 2B**	**18787**	**0.44**	**-0.82**	**0.01**	**< 0.01**	↓
**P04746**	**Pancreatic alpha-amylase**	**13239**	**0.43**	**-0.85**	**0.01**	**< 0.01**	↓
**P0DUB6**	**Alpha-amylase 1A**	**21876**	**0.38**	**-0.96**	**0.01**	**< 0.01**	↓
**P0DTE7**	**Alpha-amylase 1B**	**21876**	**0.37**	**-0.99**	**0.01**	**< 0.01**	↓
**Q96DR5**	**BPI fold-containing family A member 2**	**323**	**0.30**	**-1.20**	**0.04**	**< 0.01**	↓
**P02814**	**Submaxillary gland androgen-regulated protein 3B**	**28616**	**0.29**	**-1.25**	**0.01**	**< 0.01**	↓
**P01037**	**Cystatin-SN**	**21655**	**0.21**	**-1.55**	**0.01**	**< 0.01**	↓
**P23280**	**Carbonic anhydrase 6**	**157**	**0.16**	**-1.84**	**0.04**	**< 0.01**	↓
**P69892**	**Hemoglobin subunit gamma-2**	**514**	**0.14**	**-1.95**	**0.05**	**< 0.01**	↓
**P02100**	**Hemoglobin subunit epsilon**	**514**	**0.14**	**-1.96**	**0.04**	**< 0.01**	↓
**P69891**	**Hemoglobin subunit gamma-1**	**514**	**0.14**	**-1.96**	**0.06**	**< 0.01**	↓
**P04280**	**Basic salivary proline-rich protein 1**	**5717**	**0.11**	**-2.19**	**0.03**	**< 0.01**	↓
**P02812**	**Basic salivary proline-rich protein 2**	**6851**	**0.06**	**-2.74**	**0.05**	**< 0.01**	↓
P63104	14-3-3 protein zeta/delta	239	-	-	-	-	NP
P02763	Alpha-1-acid glycoprotein 1	238	-	-	-	-	NP
P02765	Alpha-2-HS-glycoprotein	143	-	-	-	-	NP
O43707	Alpha-actinin-4	53	-	-	-	-	NP
P03973	Antileukoproteinase	482	-	-	-	-	NP
Q66GS9	Centrosomal protein of 135 kDa	61	-	-	-	-	NP
P01024	Complement C3	94	-	-	-	-	NP
P54108	Cysteine-rich secretory protein 3	55	-	-	-	-	NP
Q01469	Fatty acid-binding protein 5	476	-	-	-	-	NP
Q5W0V3	FHF complex subunit HOOK interacting protein 2A	78	-	-	-	-	NP
P02675	Fibrinogen beta chain	394	-	-	-	-	NP
P04075	Fructose-bisphosphate aldolase A	141	-	-	-	-	NP
P09211	Glutathione S-transferase P	267	-	-	-	-	NP
P02008	Hemoglobin subunit zeta	241	-	-	-	-	NP
A0A075B6P5	Immunoglobulin kappa variable 2-28	201	-	-	-	-	NP
A2NJV5	Immunoglobulin kappa variable 2-29	201	-	-	-	-	NP
P06310	Immunoglobulin kappa variable 2-30	201	-	-	-	-	NP
A0A087WW87	Immunoglobulin kappa variable 2-40	201	-	-	-	-	NP
A0A0A0MRZ7	Immunoglobulin kappa variable 2D-26	201	-	-	-	-	NP
P01615	Immunoglobulin kappa variable 2D-28	201	-	-	-	-	NP
A0A075B6S2	Immunoglobulin kappa variable 2D-29	201	-	-	-	-	NP
A0A075B6S6	Immunoglobulin kappa variable 2D-30	201	-	-	-	-	NP
P01614	Immunoglobulin kappa variable 2D-40	201	-	-	-	-	NP
Q2TBA0	Kelch-like protein 40	26	-	-	-	-	NP
Q5T7N2	LINE-1 type transposase domain-containing protein 1	30	-	-	-	-	NP
P24158	Myeloblastin	84	-	-	-	-	NP
P80188	Neutrophil gelatinase-associated lipocalin	1032	-	-	-	-	NP
Q9BXW6	Oxysterol-binding protein-related protein 1	50	-	-	-	-	NP

P62937	Peptidyl-prolyl cis-trans isomerase A	508	-	-	-	-	NP
Q9Y536	Peptidyl-prolyl cis-trans isomerase A-like 4A	353	-	-	-	-	NP
P00558	Phosphoglycerate kinase 1	62	-	-	-	-	NP
P07205	Phosphoglycerate kinase 2	70	-	-	-	-	NP
Q6UXT9	Protein ABHD15	61	-	-	-	-	NP
Q6P5S2	Protein LEG1 homolog	336	-	-	-	-	NP
Q9BVG4	Protein PBDC1	36	-	-	-	-	NP
Q96LQ0	Protein phosphatase 1 regulatory subunit 36	77	-	-	-	-	NP
Q9C0D5	Protein TANC1	28	-	-	-	-	NP
Q8NCN5	Pyruvate dehydrogenase phosphatase regulatory subunit, mitochondrial	30	-	-	-	-	NP
Q9UBC9	Small proline-rich protein 3	367	-	-	-	-	NP
Q99757	Thioredoxin, mitochondrial	95	-	-	-	-	NP
P02774	Vitamin D-binding protein	85	-	-	-	-	NP
Q14508	WAP four-disulfide core domain protein 2	151	-	-	-	-	NP
Q6ZQQ6	WD repeat-containing protein 87	10	-	-	-	-	NP
Q8TDL5	BPI fold-containing family B member 1	96	-	-	-	-	NWP
P11021	Endoplasmic reticulum chaperone BiP	169	-	-	-	-	NWP
P17066	Heat shock 70 kDa protein 6	261	-	-	-	-	NWP
P11142	Heat shock cognate 71 kDa protein	177	-	-	-	-	NWP
P54652	Heat shock-related 70 kDa protein 2	177	-	-	-	-	NWP
Q9HCD5	Nuclear receptor coactivator 5	46	-	-	-	-	NWP
Q16378	Proline-rich protein 4	2753	-	-	-	-	NWP
Q8N6L0	Protein KASH5	52	-	-	-	-	NWP
P48741	Putative heat shock 70 kDa protein 7	261	-	-	-	-	NWP
Q9NTJ3	Structural maintenance of chromosomes protein 4	43	-	-	-	-	NWP
P49848	Transcription initiation factor TFIID subunit 6	51	-	-	-	-	NWP
P04406	Glyceraldehyde-3-phosphate dehydrogenase	29	1.27	0.24	0.21	0.84	SE
P09104	Gamma-enolase	16	1.12	0.11	0.44	0.52	SE
P34931	Heat shock 70 kDa protein 1-like	232	1.11	0.10	0.18	0.72	SE
P02788	Lactotransferrin	82	1.09	0.09	0.16	0.75	SE
Q6S8J3	POTE ankyrin domain family member E	3652	1.09	0.09	0.08	0.86	SE
P01833	Polymeric immunoglobulin receptor	6275	1.03	0.03	0.02	0.92	SE
Q9BYX7	Putative beta-actin-like protein 3	3325	1.03	0.03	0.10	0.59	SE
A5A3E0	POTE ankyrin domain family member F	3652	1.02	0.02	0.07	0.55	SE
P60709	Actin, cytoplasmic 1	7700	1.01	0.01	0.05	0.51	SE
P06744	Glucose-6-phosphate isomerase	137	1.01	0.01	0.62	0.54	SE
P0DMV9	Heat shock 70 kDa protein 1B	240	0.98	-0.02	0.20	0.44	SE
P01034	Cystatin-C	2999	0.97	-0.03	0.05	0.29	SE
P06733	Alpha-enolase	231	0.94	-0.06	0.08	0.29	SE
P63267	Actin, gamma-enteric smooth muscle	5916	0.92	-0.08	0.10	0.19	SE
P07737	Profilin-1	1509	0.91	-0.09	0.08	0.16	SE
P14618	Pyruvate kinase PKM	214	0.91	-0.09	0.14	0.28	SE
P13796	Plastin-2	295	0.89	-0.12	0.25	0.24	SE

P0DMV8	Heat shock 70 kDa protein 1A	256	0.79	-0.23	0.16	0.11	SE
P52566	Rho GDP-dissociation inhibitor 2	90	0.79	-0.23	0.19	0.13	SE
P30613	Pyruvate kinase PKLR	45	0.76	-0.28	0.25	0.21	SE
P06396	Gelsolin	57	0.63	-0.46	0.39	0.21	SE
P02808	Statherin	3641	0.62	-0.48	0.82	0.33	SE
P13929	Beta-enolase	44	0.61	-0.49	0.25	0.07	SE
P15516	Histatin-3	2572	0.56	-0.58	0.76	0.41	SE
P15515	Histatin-1	4751	0.51	-0.68	0.39	0.34	SE

Note: Ratio NP/NWP (fold change) = ratio between women with normal BMI but with periodontitis and control group proteins (women with normal BMI and without periodontitis); Log(e) ("e" is a constant = 2.71); SD, standard deviation; *p*, statistical significance (adjusted by False Discovery Rate–FDR = 4); ↑ = up-regulated (1-p > 0.95); ↓ = down-regulated (p < 0.05); SE = similar expression compared to control group; bold lines refer to up- or down-regulated proteins by more than 2-fold

S2–Table C. Proteins identified in saliva of OP and NP during T2 and their differences in expression

Accession number	Protein name	Score	Ratio OP/NP	Log(e)	SD	*p*	Expression differences
**P04075**	**Fructose-bisphosphate aldolase A**	**141**	**3.86**	**1.35**	**0.23**	**< 0.01**	↑
**P52209**	**6-phosphogluconate dehydrogenase, decarboxylating**	**116**	**3.46**	**1.24**	**0.08**	**< 0.01**	↑
**P07737**	**Profilin-1**	**2737**	**3.39**	**1.22**	**0.04**	**< 0.01**	↑
**Q01469**	**Fatty acid-binding protein 5**	**476**	**3.25**	**1.18**	**0.27**	**0.01**	↑
**P00338**	**L-lactate dehydrogenase A chain**	**161**	**2.75**	**1.01**	**0.16**	**< 0.01**	↑
**P52566**	**Rho GDP-dissociation inhibitor 2**	**252**	**2.69**	**0.99**	**0.12**	**< 0.01**	↑
**P05109**	**Protein S100-A8**	**8263**	**2.61**	**0.96**	**0.05**	**< 0.01**	↑
**P80188**	**Neutrophil gelatinase-associated lipocalin**	**1032**	**2.56**	**0.94**	**0.10**	**< 0.01**	↑
**P14618**	**Pyruvate kinase PKM**	**616**	**2.34**	**0.85**	**0.12**	**< 0.01**	↑
**P04746**	**Pancreatic alpha-amylase**	**15692**	**2.29**	**0.83**	**0.01**	**< 0.01**	↑
**P01876**	**Immunoglobulin heavy constant alpha 1**	**12347**	**2.27**	**0.82**	**0.01**	**< 0.01**	↑
**P31025**	**Lipocalin-1**	**3571**	**2.23**	**0.80**	**0.05**	**< 0.01**	↑
**Q9UGM3**	**Deleted in malignant brain tumors 1 protein**	**218**	**2.16**	**0.77**	**0.05**	**< 0.01**	↑
**Q5VSP4**	**Putative lipocalin 1-like protein 1**	**3437**	**2.14**	**0.76**	**0.06**	**< 0.01**	↑
Q14508	WAP four-disulfide core domain protein 2	151	1.95	0.67	0.19	< 0.01	↑
Q96DR5	BPI fold-containing family A member 2	436	1.82	0.60	0.05	< 0.01	↑
P0DOX2	Immunoglobulin alpha-2 heavy chain	4357	1.80	0.59	0.01	< 0.01	↑
P34931	Heat shock 70 kDa protein 1-like	119	1.72	0.54	0.18	< 0.01	↑
P0DMV9	Heat shock 70 kDa protein 1B	135	1.70	0.53	0.20	< 0.01	↑
P0DMV8	Heat shock 70 kDa protein 1A	142	1.68	0.52	0.16	< 0.01	↑
Q6P5S2	Protein LEG1 homolog	336	1.67	0.51	0.15	0.01	↑
P37837	Transaldolase	168	1.52	0.42	0.16	< 0.01	↑
P01037	Cystatin-SN	9122	1.46	0.38	0.03	< 0.01	↑
P0DOY2	Immunoglobulin lambda constant 2	2811	1.46	0.38	0.04	< 0.01	↑
P01036	Cystatin-S	9562	1.38	0.32	0.02	< 0.01	↑

P09228	Cystatin-SA	3667	1.36	0.31	0.06	< 0.01	↑
P63267	Actin, gamma-enteric smooth muscle	5281	1.34	0.29	0.03	< 0.01	↑
P0DTE7	Alpha-amylase 1B	24717	1.34	0.29	0.01	< 0.01	↑
P0CG39	POTE ankyrin domain family member J	995	1.34	0.29	0.06	< 0.01	↑
P68133	Actin, alpha skeletal muscle	5299	1.32	0.28	0.03	< 0.01	↑
P62736	Actin, aortic smooth muscle	5303	1.32	0.28	0.03	< 0.01	↑
P0DTE8	Alpha-amylase 1C	24717	1.32	0.28	0.01	< 0.01	↑
P19961	Alpha-amylase 2B	20514	1.31	0.27	0.01	< 0.01	↑
P01591	Immunoglobulin J chain	1967	1.31	0.27	0.07	< 0.01	↑
P68032	Actin, alpha cardiac muscle 1	5299	1.30	0.26	0.04	< 0.01	↑
Q6S8J3	POTE ankyrin domain family member E	2018	1.26	0.23	0.05	< 0.01	↑
P60709	Actin, cytoplasmic 1	11816	1.25	0.22	0.03	< 0.01	↑
A5A3E0	POTE ankyrin domain family member F	1926	1.25	0.22	0.05	< 0.01	↑
P63261	Actin, cytoplasmic 2	11816	1.23	0.21	0.04	< 0.01	↑
P0CG38	POTE ankyrin domain family member I	1323	1.23	0.21	0.06	< 0.01	↑
O95196	Chondroitin sulfate proteoglycan 5	79	1.17	0.16	0.06	0.02	↑
P0DUB6	Alpha-amylase 1A	24717	1.15	0.14	0.01	< 0.01	↑
P01877	Immunoglobulin heavy constant alpha 2	4619	0.94	-0.06	0.02	< 0.01	↓
P01860	Immunoglobulin heavy constant gamma 3	986	0.90	-0.10	0.05	0.03	↓
P01833	Polymeric immunoglobulin receptor	3752	0.90	-0.10	0.03	< 0.01	↓
Q562R1	Beta-actin-like protein 2	2076	0.89	-0.12	0.07	0.03	↓
P0DOX5	Immunoglobulin gamma-1 heavy chain	5720	0.88	-0.13	0.03	< 0.01	↓
P01857	Immunoglobulin heavy constant gamma 1	5729	0.88	-0.13	0.03	< 0.01	↓
P06702	Protein S100-A9	13909	0.78	-0.25	0.07	< 0.01	↓
Q8N4F0	BPI fold-containing family B member 2	197	0.76	-0.28	0.10	0.01	↓
P23280	Carbonic anhydrase 6	221	0.69	-0.37	0.06	< 0.01	↓
P02814	Submaxillary gland androgen-regulated protein 3B	23046	0.69	-0.37	0.01	< 0.01	↓
P0DOX6	Immunoglobulin mu heavy chain	71	0.68	-0.38	0.07	< 0.01	↓
P69891	Hemoglobin subunit gamma-1	748	0.67	-0.40	0.11	< 0.01	↓
P01871	Immunoglobulin heavy constant mu	71	0.66	-0.41	0.07	< 0.01	↓
P01861	Immunoglobulin heavy constant gamma 4	675	0.64	-0.44	0.07	< 0.01	↓
P13796	Plastin-2	262	0.64	-0.44	0.16	0.01	↓
P02787	Serotransferrin	1159	0.64	-0.44	0.04	< 0.01	↓
P02100	Hemoglobin subunit epsilon	748	0.64	-0.45	0.14	< 0.01	↓
Q9BYX7	Putative beta-actin-like protein 3	1077	0.64	-0.45	0.08	< 0.01	↓
P69892	Hemoglobin subunit gamma-2	748	0.63	-0.46	0.12	< 0.01	↓
P02675	Fibrinogen beta chain	394	0.63	-0.47	0.13	< 0.01	↓
P22079	Lactoperoxidase	141	0.63	-0.47	0.17	0.02	↓
P01834	Immunoglobulin kappa constant	5873	0.60	-0.51	0.05	< 0.01	↓
P0DOX7	Immunoglobulin kappa light chain	2177	0.59	-0.53	0.05	< 0.01	↓
P02812	Basic salivary proline-rich protein 2	2639	0.57	-0.56	0.10	< 0.01	↓
P68871	Hemoglobin subunit beta	4087	0.57	-0.56	0.03	< 0.01	↓
P04280	Basic salivary proline-rich protein 1	1572	0.56	-0.58	0.12	0.03	↓
P01034	Cystatin-C	289	0.55	-0.60	0.08	< 0.01	↓

P00739	Haptoglobin-related protein	19	0.51	-0.67	0.20	< 0.01	↓
P02768	**Albumin**	**24819**	**0.50**	**-0.69**	**0.02**	**< 0.01**	↓
P0CF74	**Immunoglobulin lambda constant 6**	**2577**	**0.48**	**-0.73**	**0.05**	**< 0.01**	↓
P0DOY3	**Immunoglobulin lambda constant 3**	**2811**	**0.48**	**-0.74**	**0.06**	**< 0.01**	↓
P0DOX8	**Immunoglobulin lambda-1 light chain**	**1892**	**0.47**	**-0.75**	**0.05**	**< 0.01**	↓
P01859	**Immunoglobulin heavy constant gamma 2**	**583**	**0.46**	**-0.78**	**0.10**	**< 0.01**	↓
B9A064	**Immunoglobulin lambda-like polypeptide 5**	**1892**	**0.46**	**-0.78**	**0.05**	**< 0.01**	↓
P10599	**Thioredoxin**	**152**	**0.45**	**-0.80**	**0.15**	**< 0.01**	↓
P0CG04	**Immunoglobulin lambda constant 1**	**1892**	**0.44**	**-0.83**	**0.05**	**< 0.01**	↓
P00738	**Haptoglobin**	**184**	**0.40**	**-0.91**	**0.07**	**< 0.01**	↓
P02647	**Apolipoprotein A-I**	**1550**	**0.37**	**-0.99**	**0.06**	**< 0.01**	↓
P09211	**Glutathione S-transferase P**	**267**	**0.37**	**-1.00**	**0.19**	**< 0.01**	↓
P59665	**Neutrophil defensin 1**	**3782**	**0.36**	**-1.02**	**0.11**	**< 0.01**	↓
P59666	**Neutrophil defensin 3**	**3782**	**0.34**	**-1.07**	**0.08**	**< 0.01**	↓
P04080	**Cystatin-B**	**975**	**0.33**	**-1.11**	**0.04**	**< 0.01**	↓
P02790	**Hemopexin**	**187**	**0.31**	**-1.18**	**0.06**	**< 0.01**	↓
P12273	**Prolactin-inducible protein**	**12382**	**0.30**	**-1.21**	**0.02**	**< 0.01**	↓
P02042	**Hemoglobin subunit delta**	**976**	**0.26**	**-1.35**	**0.03**	**< 0.01**	↓
Q96DA0	**Zymogen granule protein 16 homolog B**	**6029**	**0.24**	**-1.42**	**0.03**	**< 0.01**	↓
P61626	**Lysozyme C**	**3594**	**0.23**	**-1.47**	**0.05**	**< 0.01**	↓
P01023	**Alpha-2-macroglobulin**	**144**	**0.21**	**-1.58**	**0.04**	**< 0.01**	↓
P02810	**Salivary acidic proline-rich phosphoprotein 1/2**	**3280**	**0.20**	**-1.61**	**0.04**	**< 0.01**	↓
P01009	**Alpha-1-antitrypsin**	**70**	**0.18**	**-1.70**	**0.08**	**< 0.01**	↓
P69905	**Hemoglobin subunit alpha**	**2143**	**0.10**	**-2.26**	**0.01**	**< 0.01**	↓
P28325	**Cystatin-D**	**181**	**0.09**	**-2.38**	**0.05**	**< 0.01**	↓
Q8TAX7	**Mucin-7**	**1008**	**0.07**	**-2.68**	**0.02**	**< 0.01**	↓
Q5T7N2	**LINE-1 type transposase domain-containing protein 1**	**30**	**0.05**	**-3.00**	**0.03**	**< 0.01**	↓
P0DP23	Calmodulin-1	281	-	-	-	-	OP
P0DP24	Calmodulin-2	281	-	-	-	-	OP
P0DP25	Calmodulin-3	281	-	-	-	-	OP
P27482	Calmodulin-like protein 3	502	-	-	-	-	OP
Q8N126	Cell adhesion molecule 3	61	-	-	-	-	OP
P23528	Cofilin-1	295	-	-	-	-	OP
Q8N998	Coiled-coil domain-containing protein 89	34	-	-	-	-	OP
Q9UBG3	Cornulin	526	-	-	-	-	OP
Q14181	DNA polymerase alpha subunit B	43	-	-	-	-	OP
P49792	E3 SUMO-protein ligase RanBP2	13	-	-	-	-	OP
P11021	Endoplasmic reticulum chaperone BiP	19	-	-	-	-	OP
Q9BX51	Glutathione hydrolase light chain 1	129	-	-	-	-	OP
P11142	Heat shock cognate 71 kDa protein	19	-	-	-	-	OP
P54652	Heat shock-related 70 kDa protein 2	19	-	-	-	-	OP
Q86Z02	Homeodomain-interacting protein kinase 1	26	-	-	-	-	OP
O76013	Keratin, type I cuticular Ha6	82	-	-	-	-	OP

Q8IZ02	Leucine-rich repeat-containing protein 34	20	-	-	-	-	OP
P26038	Moesin	68	-	-	-	-	OP
O15105	Mothers against decapentaplegic homolog 7	60	-	-	-	-	OP
P62942	Peptidyl-prolyl cis-trans isomerase FKBP1A	238	-	-	-	-	OP
Q96BP3	Peptidylprolyl isomerase domain and WD repeat-containing protein 1	21	-	-	-	-	OP
P55201	Peregrin	37	-	-	-	-	OP
Q99986	Serine/threonine-protein kinase VRK1	87	-	-	-	-	OP
P29508	Serpin B3	199	-	-	-	-	OP
P48594	Serpin B4	181	-	-	-	-	OP
Q9H299	SH3 domain-binding glutamic acid-rich-like protein 3	2120	-	-	-	-	OP
Q9Y4F4	TOG array regulator of axonemal microtubules protein 1	27	-	-	-	-	OP
P02766	Transthyretin	211	-	-	-	-	OP
P63104	14-3-3 protein zeta/delta	239	-	-	-	-	NP
P02763	Alpha-1-acid glycoprotein 1	238	-	-	-	-	NP
P02765	Alpha-2-HS-glycoprotein	143	-	-	-	-	NP
O43707	Alpha-actinin-4	53	-	-	-	-	NP
P03973	Antileukoproteinase	482	-	-	-	-	NP
Q66GS9	Centrosomal protein of 135 kDa	61	-	-	-	-	NP
P06396	Gelsolin	126	-	-	-	-	NP
P02008	Hemoglobin subunit zeta	241	-	-	-	-	NP
P15515	Histatin-1	588	-	-	-	-	NP
Q2TBA0	Kelch-like protein 40	26	-	-	-	-	NP
P24158	Myeloblastin	84	-	-	-	-	NP
Q9BXW6	Oxysterol-binding protein-related protein 1	50	-	-	-	-	NP
Q9Y536	Peptidyl-prolyl cis-trans isomerase A-like 4A	353	-	-	-	-	NP
P07205	Phosphoglycerate kinase 2	70	-	-	-	-	NP
Q6UXT9	Protein ABHD15	61	-	-	-	-	NP
Q9BVG4	Protein PBDC1	36	-	-	-	-	NP
Q96LQ0	Protein phosphatase 1 regulatory subunit 36	77	-	-	-	-	NP
Q9C0D5	Protein TANC1	28	-	-	-	-	NP
Q8NCN5	Pyruvate dehydrogenase phosphatase regulatory subunit, mitochondrial	30	-	-	-	-	NP
P30613	Pyruvate kinase PKLR	50	-	-	-	-	NP
Q99757	Thioredoxin, mitochondrial	95	-	-	-	-	NP
P02774	Vitamin D-binding protein	85	-	-	-	-	NP
Q6ZQQ6	WD repeat-containing protein 87	10	-	-	-	-	NP
P61769	Beta-2-microglobulin	576	2.48	0.91	0.40	0.92	SE
P54108	Cysteine-rich secretory protein 3	55	1.48	0.39	0.29	0.91	SE
P02808	Statherin	5950	1.34	0.29	0.81	0.53	SE
Q9UBC9	Small proline-rich protein 3	367	1.26	0.23	0.25	0.77	SE
Q01518	Adenylyl cyclase-associated protein 1	201	1.25	0.22	0.34	0.78	SE
P04406	Glyceraldehyde-3-phosphate dehydrogenase	868	1.15	0.14	0.12	0.87	SE
A0A087WW87	Immunoglobulin kappa variable 2-40	201	1.15	0.14	0.20	0.72	SE

P01615	Immunoglobulin kappa variable 2D-28	201	1.15	0.14	0.18	0.75	SE
A0A075B6S2	Immunoglobulin kappa variable 2D-29	201	1.13	0.12	0.17	0.76	SE
A0A075B6P5	Immunoglobulin kappa variable 2-28	201	1.12	0.11	0.18	0.74	SE
A0A075B6S6	Immunoglobulin kappa variable 2D-30	201	1.11	0.10	0.21	0.71	SE
P06310	Immunoglobulin kappa variable 2-30	201	1.09	0.09	0.17	0.65	SE
A0A0A0MRZ7	Immunoglobulin kappa variable 2D-26	201	1.09	0.09	0.20	0.68	SE
P01614	Immunoglobulin kappa variable 2D-40	201	1.09	0.09	0.14	0.65	SE
P06733	Alpha-enolase	920	1.07	0.07	0.06	0.84	SE
A2NJV5	Immunoglobulin kappa variable 2-29	201	1.07	0.07	0.22	0.69	SE
P09104	Gamma-enolase	152	1.04	0.04	0.38	0.56	SE
P01024	Complement C3	94	0.93	-0.07	0.09	0.21	SE
P06744	Glucose-6-phosphate isomerase	141	0.93	-0.07	0.32	0.44	SE
Q5W0V3	FHF complex subunit HOOK interacting protein 2A	78	0.90	-0.10	0.19	0.34	SE
A0M8Q6	Immunoglobulin lambda constant 7	741	0.89	-0.12	0.13	0.19	SE
P02788	Lactotransferrin	320	0.89	-0.12	0.11	0.20	SE
P13929	Beta-enolase	226	0.87	-0.14	0.28	0.36	SE
P00558	Phosphoglycerate kinase 1	62	0.83	-0.19	0.19	0.20	SE
P29401	Transketolase	119	0.80	-0.22	0.47	0.41	SE
P02679	Fibrinogen gamma chain	418	0.79	-0.23	0.12	0.06	SE
Q8NHQ9	ATP-dependent RNA helicase DDX55	186	0.73	-0.31	0.55	0.51	SE
P62937	Peptidyl-prolyl cis-trans isomerase A	508	0.70	-0.36	0.24	0.10	SE
P25311	Zinc-alpha-2-glycoprotein	356	0.59	-0.52	0.26	0.05	SE
P15516	Histatin-3	1761	0.30	-1.20	0.49	0.10	SE

Note: Ratio OP/NP (fold change) = ratio between women with obesity and periodontitis and control group proteins (women with normal BMI but with periodontitis); Log(e) ("e" is a constant = 2.71); SD, standard deviation; *p*, statistical significance (adjusted by False Discovery Rate–FDR = 4); ↑ = up-regulated (1-p > 0.95); ↓ = down-regulated (p < 0.05); SE = similar expression compared to control group; bold lines refer to up- or down-regulated proteins by more than 2-fold

S2–Table D. Proteins identified in saliva of OWP and NWP during T2 and their differences in expression

Accession number	Protein name	Score	Ratio OWP/NWP	Log(e)	SD	*p*	Expression differences
**Q8TDL5**	**BPI fold-containing family B member 1**	**96**	**6.89**	**1.93**	**0.14**	**< 0.01**	↑
**Q8TAX7**	**Mucin-7**	**1302**	**6.17**	**1.82**	**0.03**	**< 0.01**	↑
**P04080**	**Cystatin-B**	**4062**	**5.99**	**1.79**	**0.03**	**< 0.01**	↑
**P10599**	**Thioredoxin**	**480**	**3.53**	**1.26**	**0.12**	**< 0.01**	↑
**P0DOY2**	**Immunoglobulin lambda constant 2**	**1261**	**2.94**	**1.08**	**0.04**	**< 0.01**	↑
**P0DOY3**	**Immunoglobulin lambda constant 3**	**1261**	**2.94**	**1.08**	**0.06**	**< 0.01**	↑
**P61769**	**Beta-2-microglobulin**	**784**	**2.89**	**1.06**	**0.06**	**< 0.01**	↑
**P0DOX8**	**Immunoglobulin lambda-1 light chain**	**1469**	**2.80**	**1.03**	**0.04**	**< 0.01**	↑
**P0CG04**	**Immunoglobulin lambda constant 1**	**1469**	**2.75**	**1.01**	**0.05**	**< 0.01**	↑
**P05109**	**Protein S100-A8**	**110**	**2.64**	**0.97**	**0.03**	**< 0.01**	↑
**B9A064**	**Immunoglobulin lambda-like polypeptide 5**	**1469**	**2.20**	**0.79**	**0.11**	**< 0.01**	↑

**Q96DR5**	**BPI fold-containing family A member 2**	**323**	**2.00**	**0.69**	**0.04**	**< 0.01**	↑
P01871	Immunoglobulin heavy constant mu	148	1.92	0.65	0.10	< 0.01	↑
P0DOX6	Immunoglobulin mu heavy chain	148	1.92	0.65	0.10	< 0.01	↑
P00739	Haptoglobin-related protein	68	1.90	0.64	0.18	< 0.01	↑
P0CF74	Immunoglobulin lambda constant 6	946	1.90	0.64	0.05	< 0.01	↑
P29401	Transketolase	115	1.80	0.59	0.16	< 0.01	↑
P02768	Albumin OS=Homo sapiens	15244	1.75	0.56	0.01	< 0.01	↑
P01834	Immunoglobulin kappa constant	2112	1.63	0.49	0.05	< 0.01	↑
P0DOX7	Immunoglobulin kappa light chain	317	1.63	0.49	0.04	< 0.01	↑
P01876	Immunoglobulin heavy constant alpha 1	7226	1.54	0.43	0.01	< 0.01	↑
P01877	Immunoglobulin heavy constant alpha 2	3603	1.54	0.43	0.01	< 0.01	↑
Q8N4F0	BPI fold-containing family B member 2	343	1.49	0.40	0.07	< 0.01	↑
P59665	Neutrophil defensin 1	887	1.32	0.28	0.11	0.02	↑
P59666	Neutrophil defensin 3	887	1.28	0.25	0.11	0.02	↑
P0DOX2	Immunoglobulin alpha-2 heavy chain	3309	1.27	0.24	0.08	< 0.01	↑
P68032	Actin, alpha cardiac muscle 1	5916	1.19	0.17	0.07	0.01	↑
P02812	Basic salivary proline-rich protein 2	6851	1.14	0.13	0.03	< 0.01	↑
P31025	Lipocalin-1	3326	1.12	0.11	0.04	< 0.01	↑
P07737	Profilin-1	1509	0.86	-0.15	0.08	0.04	↓
P0DTE7	Alpha-amylase 1B	21876	0.85	-0.16	0.01	< 0.01	↓
P01833	Polymeric immunoglobulin receptor	6275	0.85	-0.16	0.02	< 0.01	↓
P04746	Pancreatic alpha-amylase	13239	0.84	-0.17	0.01	< 0.01	↓
P19961	Alpha-amylase 2B	18787	0.84	-0.18	0.01	< 0.01	↓
Q9BYX7	Putative beta-actin-like protein 3	3325	0.80	-0.22	0.12	0.04	↓
P23280	Carbonic anhydrase 6	157	0.79	-0.24	0.07	< 0.01	↓
P01034	Cystatin-C	2999	0.79	-0.24	0.07	0.01	↓
P02787	Serotransferrin	703	0.76	-0.27	0.05	< 0.01	↓
P02790	Hemopexin	468	0.75	-0.29	0.12	0.01	↓
P13796	Plastin-2	295	0.73	-0.31	0.13	0.02	↓
P02810	Salivary acidic proline-rich phosphoprotein 1/2	2359	0.71	-0.34	0.03	< 0.01	↓
P0DUB6	Alpha-amylase 1A	21876	0.70	-0.35	0.01	< 0.01	↓
P0DTE8	Alpha-amylase 1C	21876	0.70	-0.35	0.01	< 0.01	↓
P01861	Immunoglobulin heavy constant gamma 4	224	0.69	-0.37	0.10	< 0.01	↓
A5A3E0	POTE ankyrin domain family member F	3652	0.67	-0.40	0.07	< 0.01	↓
Q96DA0	Zymogen granule protein 16 homolog B	6943	0.67	-0.40	0.04	< 0.01	↓
P37837	Transaldolase	133	0.64	-0.44	0.19	0.03	↓
Q6S8J3	POTE ankyrin domain family member E	3652	0.63	-0.46	0.08	< 0.01	↓
P0CG39	POTE ankyrin domain family member J	457	0.61	-0.50	0.10	< 0.01	↓
P69891	Hemoglobin subunit gamma-1	514	0.58	-0.54	0.21	< 0.01	↓
P01860	Immunoglobulin heavy constant gamma 3	377	0.58	-0.54	0.07	< 0.01	↓
P02647	Apolipoprotein A-I	905	0.57	-0.56	0.07	< 0.01	↓
P69892	Hemoglobin subunit gamma-2	514	0.56	-0.58	0.23	< 0.01	↓
Q01518	Adenylyl cyclase-associated protein 1	464	0.55	-0.60	0.21	0.01	↓
P02100	Hemoglobin subunit epsilon	514	0.55	-0.60	0.23	< 0.01	↓
P0CG38	POTE ankyrin domain family member I	559	0.55	-0.60	0.08	< 0.01	↓

Q9UGM3	Deleted in malignant brain tumors 1 protein	153	0.54	-0.61	0.09	< 0.01	↓
P01857	Immunoglobulin heavy constant gamma 1	3271	0.53	-0.64	0.03	< 0.01	↓
P0DOX5	Immunoglobulin gamma-1 heavy chain	3271	0.52	-0.65	0.04	< 0.01	↓
**P01037**	**Cystatin-SN**	**21655**	**0.46**	**-0.77**	**0.02**	**< 0.01**	↓
**P68871**	**Hemoglobin subunit beta**	**1059**	**0.44**	**-0.82**	**0.04**	**< 0.01**	↓
**P01036**	**Cystatin-S OS=Homo sapiens**	**15763**	**0.42**	**-0.86**	**0.02**	**< 0.01**	↓
**P12273**	**Prolactin-inducible protein**	**13097**	**0.39**	**-0.93**	**0.02**	**< 0.01**	↓
**P69905**	**Hemoglobin subunit alpha**	**339**	**0.38**	**-0.96**	**0.18**	**< 0.01**	↓
**P02042**	**Hemoglobin subunit delta**	**2300**	**0.37**	**-0.99**	**0.06**	**< 0.01**	↓
**P09228**	**Cystatin-SA**	**6385**	**0.27**	**-1.31**	**0.02**	**< 0.01**	↓
**A0M8Q6**	**Immunoglobulin lambda constant 7**	**199**	**0.24**	**-1.43**	**0.05**	**< 0.01**	↓
**P04280**	**Basic salivary proline-rich protein 1**	**5717**	**0.21**	**-1.58**	**0.04**	**< 0.01**	↓
**P02808**	**Statherin**	**3641**	**0.20**	**-1.63**	**0.03**	**< 0.01**	↓
**P17066**	**Heat shock 70 kDa protein 6**	**261**	**0.18**	**-1.69**	**0.19**	**0.01**	↓
**P48741**	**Putative heat shock 70 kDa protein 7**	**261**	**0.16**	**-1.81**	**0.17**	**< 0.01**	↓
**P02814**	**Submaxillary gland androgen-regulated protein 3B**	**28616**	**0.13**	**-2.04**	**0.01**	**< 0.01**	↓
**P15516**	**Histatin-3**	**2572**	**0.12**	**-2.14**	**0.46**	**0.01**	↓
P07108	Acyl-CoA-binding protein	235	-	-	-	-	OWP
P02763	Alpha-1-acid glycoprotein 1	116	-	-	-	-	OWP
A8K2U0	Alpha-2-macroglobulin-like protein 1	33	-	-	-	-	OWP
P27482	Calmodulin-like protein 3	121	-	-	-	-	OWP
Q5SW79	Centrosomal protein of 170 kDa	150	-	-	-	-	OWP
P01024	Complement C3	49	-	-	-	-	OWP
Q9UBG3	Cornulin	48	-	-	-	-	OWP
P33991	DNA replication licensing factor MCM4	19	-	-	-	-	OWP
P32519	ETS-related transcription factor Elf-1	27	-	-	-	-	OWP
Q01469	Fatty acid-binding protein 5	582	-	-	-	-	OWP
P02675	Fibrinogen beta chain	160	-	-	-	-	OWP
Q08380	Galectin-3-binding protein	15	-	-	-	-	OWP
P09211	Glutathione S-transferase P	85	-	-	-	-	OWP
Q969F9	Hermansky-Pudlak syndrome 3 protein	29	-	-	-	-	OWP
Q9NR48	Histone-lysine N-methyltransferase ASH1L	17	-	-	-	-	OWP
A0A075B6P5	Immunoglobulin kappa variable 2-28	231	-	-	-	-	OWP
A2NJV5	Immunoglobulin kappa variable 2-29	231	-	-	-	-	OWP
P06310	Immunoglobulin kappa variable 2-30	231	-	-	-	-	OWP
A0A087WW87	Immunoglobulin kappa variable 2-40	231	-	-	-	-	OWP
A0A0A0MRZ7	Immunoglobulin kappa variable 2D-26	231	-	-	-	-	OWP
P01615	Immunoglobulin kappa variable 2D-28	231	-	-	-	-	OWP
A0A075B6S2	Immunoglobulin kappa variable 2D-29	231	-	-	-	-	OWP
A0A075B6S6	Immunoglobulin kappa variable 2D-30	231	-	-	-	-	OWP
P01614	Immunoglobulin kappa variable 2D-40	231	-	-	-	-	OWP
Q2TBA0	Kelch-like protein 40	41	-	-	-	-	OWP
Q9BUT9	MAPK regulated corepressor interacting protein 2	42	-	-	-	-	OWP

Q02817	Mucin-2	33	-	-	-	-	OWP
P62937	Peptidyl-prolyl cis-trans isomerase A	138	-	-	-	-	OWP
Q6P5S2	Protein LEG1 homolog	297	-	-	-	-	OWP
A8MUU1	Putative fatty acid-binding protein 5-like protein 3	227	-	-	-	-	OWP
Q9UBC9	Small proline-rich protein 3	394	-	-	-	-	OWP
Q9NSD5	Sodium- and chloride-dependent GABA transporter 2	141	-	-	-	-	OWP
O60264	SWI/SNF-related matrix-associated actin-dependent regulator of chromatin subfamily A member 5	14	-	-	-	-	OWP
Q5JTD0	Tight junction-associated protein 1	19	-	-	-	-	OWP
Q6ZVM7	TOM1-like protein 2	66	-	-	-	-	OWP
Q9BXT4	Tudor domain-containing protein 1	33	-	-	-	-	OWP
Q502W6	von Willebrand factor A domain-containing protein 3B	65	-	-	-	-	OWP
Q14508	WAP four-disulfide core domain protein 2	531	-	-	-	-	OWP
P52209	6-phosphogluconate dehydrogenase, decarboxylating	81	-	-	-	-	NWP
O95196	Chondroitin sulfate proteoglycan 5	36	-	-	-	-	NWP
P06744	Glucose-6-phosphate isomerase	137	-	-	-	-	NWP
P00338	L-lactate dehydrogenase A chain	246	-	-	-	-	NWP
Q9HCD5	Nuclear receptor coactivator 5	46	-	-	-	-	NWP
Q16378	Proline-rich protein 4	2753	-	-	-	-	NWP
Q8N6L0	Protein KASH5	52	-	-	-	-	NWP
P52566	Rho GDP-dissociation inhibitor 2	90	-	-	-	-	NWP
Q9NTJ3	Structural maintenance of chromosomes protein 4	43	-	-	-	-	NWP
P49848	Transcription initiation factor TFIID subunit 6	51	-	-	-	-	NWP
P04406	Glyceraldehyde-3-phosphate dehydrogenase	29	3.22	1.17	0.42	0.94	SE
P25311	Zinc-alpha-2-glycoprotein	80	1.25	0.22	0.19	0.83	SE
P09104	Gamma-enolase	16	1.19	0.17	0.43	0.60	SE
P62736	Actin, aortic smooth muscle	5916	1.15	0.14	0.11	0.92	SE
P00738	Haptoglobin	192	1.14	0.13	0.08	0.91	SE
P01591	Immunoglobulin J chain	4092	1.13	0.12	0.08	0.91	SE
P02788	Lactotransferrin	82	1.13	0.12	0.12	0.79	SE
P63267	Actin, gamma-enteric smooth muscle	5916	1.12	0.11	0.14	0.70	SE
P01023	Alpha-2-macroglobulin	91	1.11	0.10	0.09	0.91	SE
P15515	Histatin-1	4751	1.11	0.10	0.41	0.43	SE
P30613	Pyruvate kinase PKLR	45	1.11	0.10	0.25	0.63	SE
P06733	Alpha-enolase	231	1.05	0.05	0.07	0.71	SE
Q5VSP4	Putative lipocalin 1-like protein 1	1864	1.05	0.05	0.06	0.78	SE
P13929	Beta-enolase	44	1.04	0.04	0.20	0.57	SE
P34931	Heat shock 70 kDa protein 1-like	232	1.04	0.04	0.21	0.64	SE
P20742	Pregnancy zone protein	66	1.03	0.03	0.25	0.52	SE
P68133	Actin, alpha skeletal muscle	5916	1.00	0	0.06	0.54	SE
P60709	Actin, cytoplasmic 1	7700	1.00	0	0.08	0.54	SE

P63261	Actin, cytoplasmic 2	7700	0.98	-0.02	0.06	0.25	SE
P28325	Cystatin-D	1595	0.98	-0.02	0.05	0.34	SE
P14618	Pyruvate kinase PKM	214	0.97	-0.03	0.12	0.44	SE
Q562R1	Beta-actin-like protein 2	4453	0.92	-0.08	0.05	0.11	SE
P0DMV9	Heat shock 70 kDa protein 1B	240	0.91	-0.09	0.20	0.36	SE
Q8NHQ9	ATP-dependent RNA helicase DDX55	235	0.89	-0.12	0.67	0.45	SE
P01859	Immunoglobulin heavy constant gamma 2	228	0.89	-0.12	0.08	0.13	SE
P0DMV8	Heat shock 70 kDa protein 1A	256	0.88	-0.13	0.18	0.27	SE
P61626	Lysozyme C	484	0.86	-0.15	0.08	0.07	SE
P22079	Lactoperoxidase	68	0.84	-0.17	0.22	0.33	SE
P11021	Endoplasmic reticulum chaperone BiP	169	0.81	-0.21	0.39	0.26	SE
P01009	Alpha-1-antitrypsin	171	0.73	-0.31	0.19	0.09	SE
P06396	Gelsolin	57	0.73	-0.31	0.20	0.11	SE
P54652	Heat shock-related 70 kDa protein 2	177	0.37	-0.99	0.44	0.13	SE
P11142	Heat shock cognate 71 kDa protein	177	0.26	-1.34	0.44	0.06	SE

Note: Ratio OWP/NWP (fold change) = ratio between women with obesity but without periodontitis and control group proteins (women with normal BMI and without periodontitis); Log(e) (“e” is a constant = 2.71); SD, standard deviation; *p*, statistical significance (adjusted by False Discovery Rate–FDR = 4); ↑ = up-regulated (1-p > 0.95); ↓ = down-regulated ($p < 0.05$); SE = similar expression compared to control group; bold lines refer to up- or down-regulated proteins by more than 2-fold
