# Supplementary material for: Label-Free Quantitative Proteomic Analysis Reveals Inflammatory Pattern Associated with Obesity and Periodontitis in Pregnant Women
Source: Metabolites. 2022 Nov 10;12(11):1091. doi: 10.3390/metabo12111091 (PMC9692340; doi:10.3390/metabo12111091)
Supplement: Supplementary file 1 [file metabolites-12-01091-s001.zip › Supplementary file S3.pdf]

S3–Table A. Proteins identified in saliva in OP and their differences in expression during T1 and T2

Accession number	Protein name	Score	Ratio T1/T2	Log(e)	SD	*p*	Expression differences
**P69891**	**Hemoglobin subunit gamma-1**	**335**	**22.42**	**3.11**	**0.02**	**< 0.01**	↑
**P69892**	**Hemoglobin subunit gamma-2**	**335**	**21.76**	**3.08**	**0.02**	**< 0.01**	↑
**P02100**	**Hemoglobin subunit epsilon**	**335**	**21.12**	**3.05**	**0.02**	**0.01**	↑
**P20742**	**Pregnancy zone protein**	**24**	**4.85**	**1.58**	**0.09**	**< 0.01**	↑
**P06702**	**Protein S100-A9**	**4498**	**4.18**	**1.43**	**0.05**	**< 0.01**	↑
**P00739**	**Haptoglobin-related protein**	**65**	**4.14**	**1.42**	**0.11**	**< 0.01**	↑
**P02675**	**Fibrinogen beta chain**	**181**	**3.82**	**1.34**	**0.15**	**< 0.01**	↑
**P02679**	**Fibrinogen gamma chain**	**185**	**3.74**	**1.32**	**0.06**	**< 0.01**	↑
**Q5T7N2**	**LINE-1 type transposase domain-containing protein 1**	**18**	**3.03**	**1.11**	**0.05**	**< 0.01**	↑
**P02787**	**Serotransferrin**	**426**	**2.89**	**1.06**	**0.03**	**< 0.01**	↑
**P62937**	**Peptidyl-prolyl cis-trans isomerase A**	**243**	**2.72**	**1.00**	**0.11**	**< 0.01**	↑
**P13929**	**Beta-enolase**	**154**	**2.64**	**0.97**	**0.14**	**< 0.01**	↑
**P29401**	**Transketolase**	**114**	**2.61**	**0.96**	**0.12**	**< 0.01**	↑
**A0M8Q6**	**Immunoglobulin lambda constant 7**	**719**	**2.51**	**0.92**	**0.13**	**< 0.01**	↑
**P01034**	**Cystatin-C**	**203**	**2.46**	**0.90**	**0.05**	**< 0.01**	↑
**P10599**	**Thioredoxin**	**2278**	**2.27**	**0.82**	**0.16**	**< 0.01**	↑
**P01591**	**Immunoglobulin J chain**	**2426**	**2.12**	**0.75**	**0.04**	**< 0.01**	↑
**P0DOX6**	**Immunoglobulin mu heavy chain**	**211**	**2.03**	**0.71**	**0.09**	**< 0.01**	↑
**P01857**	**Immunoglobulin heavy constant gamma 1**	**3229**	**2.00**	**0.69**	**0.03**	**< 0.01**	↑
P02647	Apolipoprotein A-I	276	1.97	0.68	0.15	< 0.01	↑
Q8TAX7	Mucin-7	197	1.97	0.68	0.05	< 0.01	↑
P14618	Pyruvate kinase PKM	207	1.93	0.66	0.07	< 0.01	↑
P37837	Transaldolase	222	1.93	0.66	0.09	< 0.01	↑
P01859	Immunoglobulin heavy constant gamma 2	535	1.92	0.65	0.09	< 0.01	↑
P13796	Plastin-2	291	1.92	0.65	0.12	< 0.01	↑
P01024	Complement C3	82	1.84	0.61	0.07	< 0.01	↑
P00738	Haptoglobin	369	1.82	0.60	0.05	< 0.01	↑
P26038	Moesin	68	1.82	0.60	0.19	< 0.01	↑
P02766	Transthyretin	211	1.80	0.59	0.24	0.03	↑
P01861	Immunoglobulin heavy constant gamma 4	458	1.73	0.55	0.09	< 0.01	↑
P0DOX8	Immunoglobulin lambda-1 light chain	1492	1.68	0.52	0.05	< 0.01	↑
Q8NHQ9	ATP-dependent RNA helicase DDX55	184	1.67	0.51	0.25	0.01	↑
P0CG04	Immunoglobulin lambda constant 1	1450	1.67	0.51	0.05	< 0.01	↑
P02788	Lactotransferrin	116	1.67	0.51	0.09	< 0.01	↑
B9A064	Immunoglobulin lambda-like polypeptide 5	1492	1.65	0.50	0.05	< 0.01	↑
P0CF74	Immunoglobulin lambda constant 6	1450	1.63	0.49	0.05	< 0.01	↑
P01833	Polymeric immunoglobulin receptor	4846	1.60	0.47	0.02	< 0.01	↑
Q9UBG3	Cornulin	526	1.57	0.45	0.12	< 0.01	↑
P04080	Cystatin-B	10571	1.57	0.45	0.07	< 0.01	↑

P01876	Immunoglobulin heavy constant alpha 1	9018	1.57	0.45	0.01	< 0.01	↑
P0DOY2	Immunoglobulin lambda constant 2	1766	1.52	0.42	0.05	< 0.01	↑
P0DOY3	Immunoglobulin lambda constant 3	1766	1.52	0.42	0.05	< 0.01	↑
P00338	L-lactate dehydrogenase A chain	81	1.39	0.33	0.13	0.01	↑
Q9UGM3	Deleted in malignant brain tumors 1 protein	180	1.35	0.30	0.05	< 0.01	↑
Q96DR5	BPI fold-containing family A member 2	129	1.31	0.27	0.10	< 0.01	↑
P04406	Glyceraldehyde-3-phosphate dehydrogenase	142	1.30	0.26	0.11	0.01	↑
P01860	Immunoglobulin heavy constant gamma 3	655	1.30	0.26	0.06	< 0.01	↑
P01834	Immunoglobulin kappa constant	1013	1.30	0.26	0.04	< 0.01	↑
P0DOX5	Immunoglobulin gamma-1 heavy chain	3229	1.28	0.25	0.04	< 0.01	↑
P01877	Immunoglobulin heavy constant alpha 2	5207	1.21	0.19	0.01	< 0.01	↑
P0DOX7	Immunoglobulin kappa light chain	242	1.21	0.19	0.04	< 0.01	↑
P52209	6-phosphogluconate dehydrogenase. decarboxylating	200	1.20	0.18	0.10	0.02	↑
P0DOX2	Immunoglobulin alpha-2 heavy chain	4593	1.19	0.17	0.01	< 0.01	↑
P02814	Submaxillary gland androgen-regulated protein 3B	16189	1.19	0.17	0.01	< 0.01	↑
P22079	Lactoperoxidase	66	1.16	0.15	0.05	0.02	↑
Q562R1	Beta-actin-like protein 2	2598	1.15	0.14	0.05	0.01	↑
P0DTE8	Alpha-amylase 1C	31544	0.94	-0.06	0.01	< 0.01	↓
P0DUB6	Alpha-amylase 1A	31544	0.93	-0.07	0.01	< 0.01	↓
P0DTE7	Alpha-amylase 1B	31544	0.93	-0.07	0.01	< 0.01	↓
P19961	Alpha-amylase 2B	26381	0.93	-0.07	0.01	< 0.01	↓
P12273	Prolactin-inducible protein	6644	0.93	-0.07	0.03	0.02	↓
P02768	Albumin	14908	0.92	-0.08	0.02	< 0.01	↓
A5A3E0	POTE ankyrin domain family member F	1647	0.92	-0.08	0.04	0.04	↓
P04746	Pancreatic alpha-amylase	17881	0.91	-0.09	0.01	< 0.01	↓
Q6S8J3	POTE ankyrin domain family member E	1644	0.91	-0.09	0.03	< 0.01	↓
P02810	Salivary acidic proline-rich phosphoprotein 1/2	471	0.88	-0.13	0.04	< 0.01	↓
P60709	Actin. cytoplasmic 1	3921	0.85	-0.16	0.03	< 0.01	↓
P63261	Actin. cytoplasmic 2	3921	0.85	-0.16	0.04	< 0.01	↓
P0CG38	POTE ankyrin domain family member I	518	0.85	-0.16	0.05	< 0.01	↓
P0CG39	POTE ankyrin domain family member J	376	0.85	-0.16	0.04	< 0.01	↓
P05109	Protein S100-A8	8609	0.84	-0.18	0.06	< 0.01	↓
P01037	Cystatin-SN	7951	0.83	-0.19	0.02	< 0.01	↓
P63267	Actin. gamma-enteric smooth muscle	3447	0.82	-0.20	0.03	< 0.01	↓
P68032	Actin. alpha cardiac muscle 1	3447	0.81	-0.21	0.04	< 0.01	↓
P62736	Actin. aortic smooth muscle	3447	0.81	-0.21	0.03	< 0.01	↓
P01023	Alpha-2-macroglobulin	73	0.81	-0.21	0.12	0.02	↓
P68133	Actin. alpha skeletal muscle	3438	0.79	-0.23	0.03	< 0.01	↓
P80188	Neutrophil gelatinase-associated lipocalin	635	0.79	-0.23	0.08	< 0.01	↓
P02790	Hemopexin	240	0.78	-0.25	0.09	< 0.01	↓
P01036	Cystatin-S	5469	0.76	-0.27	0.02	< 0.01	↓
P31025	Lipocalin-1	10909	0.76	-0.27	0.05	< 0.01	↓
P09228	Cystatin-SA	3211	0.74	-0.30	0.03	< 0.01	↓

P01871	Immunoglobulin heavy constant mu	211	0.73	-0.32	0.09	< 0.01	↓
Q96DA0	Zymogen granule protein 16 homolog B	6349	0.69	-0.37	0.04	< 0.01	↓
P69905	Hemoglobin subunit alpha	5064	0.67	-0.40	0.03	< 0.01	↓
Q9BYX7	Putative beta-actin-like protein 3	1126	0.63	-0.47	0.11	< 0.01	↓
Q5VSP4	Putative lipocalin 1-like protein 1	6173	0.63	-0.47	0.12	< 0.01	↓
Q14508	WAP four-disulfide core domain protein 2	440	0.59	-0.53	0.16	< 0.01	↓
P27482	Calmodulin-like protein 3	502	0.57	-0.57	0.19	0.01	↓
P23280	Carbonic anhydrase 6	172	0.57	-0.57	0.06	< 0.01	↓
P61626	Lysozyme C	421	0.55	-0.59	0.06	< 0.01	↓
**Q01469**	**Fatty acid-binding protein 5**	**1736**	**0.45**	**-0.80**	**0.13**	**< 0.01**	↓
**Q9H299**	**SH3 domain-binding glutamic acid-rich-like protein 3**	**2120**	**0.42**	**-0.86**	**0.20**	**< 0.01**	↓
**P52566**	**Rho GDP-dissociation inhibitor 2**	**488**	**0.40**	**-0.91**	**0.09**	**< 0.01**	↓
**P61769**	**Beta-2-microglobulin**	**719**	**0.38**	**-0.98**	**0.10**	**< 0.01**	↓
**P59665**	**Neutrophil defensin 1**	**1074**	**0.37**	**-0.99**	**0.10**	**< 0.01**	↓
**Q8N4F0**	**BPI fold-containing family B member 2**	**227**	**0.23**	**-1.48**	**0.04**	**< 0.01**	↓
**P04075**	**Fructose-bisphosphate aldolase A**	**69**	**0.23**	**-1.49**	**0.12**	**< 0.01**	↓
**P68871**	**Hemoglobin subunit beta**	**7839**	**0.22**	**-1.51**	**0.04**	**< 0.01**	↓
**P02042**	**Hemoglobin subunit delta**	**1633**	**0.14**	**-2.00**	**0.05**	**< 0.01**	↓
**P07737**	**Profilin-1**	**924**	**0.11**	**-2.21**	**0.03**	**< 0.01**	↓
P31947	14-3-3 protein sigma	448	-	-	-	-	T1
P07108	Acyl-CoA-binding protein	826	-	-	-	-	T1
Q6P587	Acylpyruvase FAHD1. mitochondrial	568	-	-	-	-	T1
P02763	Alpha-1-acid glycoprotein 1	280	-	-	-	-	T1
P02765	Alpha-2-HS-glycoprotein	427	-	-	-	-	T1
P12814	Alpha-actinin-1	62	-	-	-	-	T1
O43707	Alpha-actinin-4	50	-	-	-	-	T1
P01019	Angiotensinogen	180	-	-	-	-	T1
P03973	Antileukoproteinase	1114	-	-	-	-	T1
Q96LR9	Apolipoprotein L domain-containing protein 1	70	-	-	-	-	T1
P00450	Ceruloplasmin	590	-	-	-	-	T1
Q92616	eIF-2-alpha kinase activator GCN1	45	-	-	-	-	T1
Q9P2K8	eIF-2-alpha kinase GCN2	306	-	-	-	-	T1
Q08380	Galectin-3-binding protein	99	-	-	-	-	T1
P06396	Gelsolin	110	-	-	-	-	T1
Q9UJ14	Glutathione hydrolase 7	114	-	-	-	-	T1
P06737	Glycogen phosphorylase. liver form	45	-	-	-	-	T1
P17066	Heat shock 70 kDa protein 6	31	-	-	-	-	T1
P15515	Histatin-1	3253	-	-	-	-	T1
P01764	Immunoglobulin heavy variable 3-23	458	-	-	-	-	T1
P01768	Immunoglobulin heavy variable 3-30	458	-	-	-	-	T1
P0DP02	Immunoglobulin heavy variable 3-30-3	458	-	-	-	-	T1
P0DP03	Immunoglobulin heavy variable 3-30-5	458	-	-	-	-	T1
P01772	Immunoglobulin heavy variable 3-33	458	-	-	-	-	T1
P01767	Immunoglobulin heavy variable 3-53	458	-	-	-	-	T1

A0A0C4DH42	Immunoglobulin heavy variable 3-66	458	-	-	-	-	T1
A0A0B4J1X5	Immunoglobulin heavy variable 3-74	458	-	-	-	-	T1
P04433	Immunoglobulin kappa variable 3-11	692	-	-	-	-	T1
A0A0A0MRZ8	Immunoglobulin kappa variable 3D-11	692	-	-	-	-	T1
Q8WYH8	Inhibitor of growth protein 5	568	-	-	-	-	T1
P18510	Interleukin-1 receptor antagonist protein	112	-	-	-	-	T1
P06870	Kallikrein-1	95	-	-	-	-	T1
Q6ZMR3	L-lactate dehydrogenase A-like 6A	24	-	-	-	-	T1
P07195	L-lactate dehydrogenase B chain	24	-	-	-	-	T1
P07864	L-lactate dehydrogenase C chain	24	-	-	-	-	T1
P14780	Matrix metalloproteinase-9	58	-	-	-	-	T1
P01033	Metalloproteinase inhibitor 1	175	-	-	-	-	T1
Q02817	Mucin-2	45	-	-	-	-	T1
Q8NCY6	Myb/SANT-like DNA-binding domain-containing protein 4	165	-	-	-	-	T1
P24158	Myeloblastin	87	-	-	-	-	T1
P80303	Nucleobindin-2	79	-	-	-	-	T1
Q8NGQ2	Olfactory receptor 6Q1	201	-	-	-	-	T1
Q9Y536	Peptidyl-prolyl cis-trans isomerase A-like 4A	130	-	-	-	-	T1
P07205	Phosphoglycerate kinase 2	19	-	-	-	-	T1
Q14651	Plastin-1	88	-	-	-	-	T1
P13797	Plastin-3	34	-	-	-	-	T1
Q16378	Proline-rich protein 4	80	-	-	-	-	T1
Q8N6L0	Protein KASH5	62	-	-	-	-	T1
Q9Y5F8	Protocadherin gamma-B7	47	-	-	-	-	T1
P48741	Putative heat shock 70 kDa protein 7	31	-	-	-	-	T1
P30613	Pyruvate kinase PKLR	80	-	-	-	-	T1
P50120	Retinol-binding protein 2	390	-	-	-	-	T1
P35326	Small proline-rich protein 2A	711	-	-	-	-	T1
P35325	Small proline-rich protein 2B	950	-	-	-	-	T1
P22532	Small proline-rich protein 2D	950	-	-	-	-	T1
P22531	Small proline-rich protein 2E	781	-	-	-	-	T1
Q96RM1	Small proline-rich protein 2F	243	-	-	-	-	T1
Q9BYE4	Small proline-rich protein 2G	539	-	-	-	-	T1
Q14515	SPARC-like protein 1	31	-	-	-	-	T1
P20061	Transcobalamin-1	81	-	-	-	-	T1
P49770	Translation initiation factor eIF-2B subunit beta	36	-	-	-	-	T1
P60174	Triosephosphate isomerase	137	-	-	-	-	T1
P36537	UDP-glucuronosyltransferase 2B10	23	-	-	-	-	T1
Q9BY64	UDP-glucuronosyltransferase 2B28	23	-	-	-	-	T1
P02774	Vitamin D-binding protein	171	-	-	-	-	T1
Q9UJU3	Zinc finger protein 112	47	-	-	-	-	T1
P0DP23	Calmodulin-1	281	-	-	-	-	T2
P0DP24	Calmodulin-2	281	-	-	-	-	T2
P0DP25	Calmodulin-3	281	-	-	-	-	T2

Q8N126	Cell adhesion molecule 3	61	-	-	-	-	T2
O95196	Chondroitin sulfate proteoglycan 5	86	-	-	-	-	T2
Q8N998	Coiled-coil domain-containing protein 89	34	-	-	-	-	T2
Q14181	DNA polymerase alpha subunit B	43	-	-	-	-	T2
P49792	E3 SUMO-protein ligase RanBP2	13	-	-	-	-	T2
Q9BX51	Glutathione hydrolase light chain 1	129	-	-	-	-	T2
Q86Z02	Homeodomain-interacting protein kinase 1	26	-	-	-	-	T2
A0A075B6P5	Immunoglobulin kappa variable 2-28	176	-	-	-	-	T2
A2NJV5	Immunoglobulin kappa variable 2-29	176	-	-	-	-	T2
P06310	Immunoglobulin kappa variable 2-30	176	-	-	-	-	T2
A0A087WW87	Immunoglobulin kappa variable 2-40	176	-	-	-	-	T2
A0A0A0MRZ7	Immunoglobulin kappa variable 2D-26	176	-	-	-	-	T2
P01615	Immunoglobulin kappa variable 2D-28	176	-	-	-	-	T2
A0A075B6S2	Immunoglobulin kappa variable 2D-29	176	-	-	-	-	T2
A0A075B6S6	Immunoglobulin kappa variable 2D-30	176	-	-	-	-	T2
P01614	Immunoglobulin kappa variable 2D-40	176	-	-	-	-	T2
O76013	Keratin. type I cuticular Ha6	82	-	-	-	-	T2
Q8IZ02	Leucine-rich repeat-containing protein 34	20	-	-	-	-	T2
O15105	Mothers against decapentaplegic homolog 7	60	-	-	-	-	T2
P62942	Peptidyl-prolyl cis-trans isomerase FKBP1A	238	-	-	-	-	T2
Q96BP3	Peptidylprolyl isomerase domain and WD repeat-containing protein 1	21	-	-	-	-	T2
P55201	Peregrin	37	-	-	-	-	T2
Q99986	Serine/threonine-protein kinase VRK1	87	-	-	-	-	T2
P29508	Serpin B3	199	-	-	-	-	T2
P48594	Serpin B4	181	-	-	-	-	T2
Q9Y4F4	TOG array regulator of axonemal microtubules protein 1	27	-	-	-	-	T2
Q9UBC9	Small proline-rich protein 3	68	1.68	0.52	0.36	0.87	SE
P02808	Statherin	6871	1.38	0.32	0.57	0.43	SE
P23528	Cofilin-1	295	1.34	0.29	0.36	0.62	SE
P25311	Zinc-alpha-2-glycoprotein	283	1.30	0.26	0.21	0.89	SE
Q01518	Adenylyl cyclase-associated protein 1	348	1.25	0.22	0.17	0.90	SE
P09211	Glutathione S-transferase P	342	1.23	0.21	0.16	0.86	SE
P28325	Cystatin-D	85	1.22	0.20	0.19	0.83	SE
P09104	Gamma-enolase	23	1.20	0.18	0.30	0.68	SE
P06744	Glucose-6-phosphate isomerase	68	1.16	0.15	0.08	0.95	SE
P02812	Basic salivary proline-rich protein 2	189	1.14	0.13	0.08	0.95	SE
P04280	Basic salivary proline-rich protein 1	189	1.13	0.12	0.11	0.89	SE
P11142	Heat shock cognate 71 kDa protein	19	1.12	0.11	0.23	0.60	SE
P59666	Neutrophil defensin 3	1074	1.12	0.11	0.09	0.84	SE
P00558	Phosphoglycerate kinase 1	123	1.12	0.11	0.11	0.80	SE
P15516	Histatin-3	1882	1.11	0.10	0.15	0.69	SE
P54652	Heat shock-related 70 kDa protein 2	19	1.09	0.09	0.22	0.65	SE
P54108	Cysteine-rich secretory protein 3	61	0.98	-0.02	0.24	0.47	SE

P11021	Endoplasmic reticulum chaperone BiP	19	0.96	-0.04	0.24	0.45	SE
P06733	Alpha-enolase	430	0.93	-0.07	0.05	0.10	SE
Q5W0V3	FHF complex subunit HOOK interacting protein 2A	199	0.89	-0.12	0.23	0.32	SE
P34931	Heat shock 70 kDa protein 1-like	73	0.87	-0.14	0.14	0.16	SE
P0DMV8	Heat shock 70 kDa protein 1A	75	0.85	-0.16	0.14	0.14	SE
P0DMV9	Heat shock 70 kDa protein 1B	75	0.82	-0.20	0.15	0.12	SE
Q6P5S2	Protein LEG1 homolog	1163	0.61	-0.49	0.40	0.41	SE

Note: Ratio T1/T2 (fold change) = ratio between pregnancy (T1) and after delivery (T2) for OP; Log(e) ("e" is a constant = 2.71); SD, standard deviation; *p*, statistical significance (adjusted by False Discovery Rate–FDR = 4); ↑ = up-regulated (1-p > 0.95); ↓ = down-regulated (p < 0.05); SE = similar expression compared to T2; bold lines refer to up- or down-regulated proteins by more than 2-fold

S3–Table B. Proteins identified in saliva of OWP and their differences in expression during T1 and T2

Accession number	Protein name	Score	Ratio T1/T2	Log(e)	SD	*p*	Expression differences
**P04280**	**Basic salivary proline-rich protein 1**	**511**	**29.08**	**3.37**	**0.02**	**< 0.01**	↑
**P02788**	**Lactotransferrin**	**148**	**8.50**	**2.14**	**0.02**	**< 0.01**	↑
**P02812**	**Basic salivary proline-rich protein 2**	**699**	**6.36**	**1.85**	**0.03**	**< 0.01**	↑
**Q14508**	**WAP four-disulfide core domain protein 2**	**531**	**6.05**	**1.80**	**0.06**	**< 0.01**	↑
**P02787**	**Serotransferrin**	**433**	**4.44**	**1.49**	**0.02**	**< 0.01**	↑
**P37837**	**Transaldolase**	**102**	**3.60**	**1.28**	**0.11**	**< 0.01**	↑
**P25311**	**Zinc-alpha-2-glycoprotein**	**95**	**3.35**	**1.21**	**0.10**	**< 0.01**	↑
**Q9UBG3**	**Cornulin**	**48**	**3.19**	**1.16**	**0.12**	**< 0.01**	↑
**P0CF74**	**Immunoglobulin lambda constant 6**	**2283**	**3.06**	**1.12**	**0.05**	**< 0.01**	↑
**P13796**	**Plastin-2**	**176**	**3.00**	**1.10**	**0.05**	**< 0.01**	↑
**P02790**	**Hemopexin**	**235**	**2.89**	**1.06**	**0.07**	**< 0.01**	↑
**A0M8Q6**	**Immunoglobulin lambda constant 7**	**1011**	**2.80**	**1.03**	**0.08**	**< 0.01**	↑
**P02675**	**Fibrinogen beta chain**	**160**	**2.77**	**1.02**	**0.16**	**< 0.01**	↑
**P14618**	**Pyruvate kinase PKM**	**213**	**2.72**	**1.00**	**0.12**	**< 0.01**	↑
**P02647**	**Apolipoprotein A-I**	**286**	**2.48**	**0.91**	**0.05**	**< 0.01**	↑
**P0DOY2**	**Immunoglobulin lambda constant 2**	**340**	**2.18**	**0.78**	**0.04**	**< 0.01**	↑
**P0CG38**	**POTE ankyrin domain family member I**	**37**	**2.14**	**0.76**	**0.05**	**< 0.01**	↑
**P0CG39**	**POTE ankyrin domain family member J**	**37**	**2.14**	**0.76**	**0.06**	**< 0.01**	↑
**P07737**	**Profilin-1**	**1975**	**2.14**	**0.76**	**0.05**	**< 0.01**	↑
**P01591**	**Immunoglobulin J chain**	**419**	**2.03**	**0.71**	**0.04**	**< 0.01**	↑
**Q562R1**	**Beta-actin-like protein 2**	**143**	**2.00**	**0.69**	**0.06**	**< 0.01**	↑
P04406	Glyceraldehyde-3-phosphate dehydrogenase	235	1.90	0.64	0.08	< 0.01	↑
Q8N4F0	BPI fold-containing family B member 2	59	1.88	0.63	0.19	< 0.01	↑
Q9BYX7	Putative beta-actin-like protein 3	1669	1.79	0.58	0.09	< 0.01	↑
Q9UGM3	Deleted in malignant brain tumors 1 protein	148	1.75	0.56	0.07	< 0.01	↑
Q5VSP4	Putative lipocalin 1-like protein 1	1296	1.72	0.54	0.04	< 0.01	↑
P63267	Actin. gamma-enteric smooth muscle	180	1.67	0.51	0.05	< 0.01	↑

Q9UBC9	Small proline-rich protein 3	394	1.67	0.51	0.19	0.02	↑
A5A3E0	POTE ankyrin domain family member F	62	1.65	0.50	0.07	< 0.01	↑
P01024	Complement C3	49	1.63	0.49	0.07	< 0.01	↑
P34931	Heat shock 70 kDa protein 1-like	146	1.63	0.49	0.15	< 0.01	↑
P02814	Submaxillary gland androgen-regulated protein 3B	2912	1.62	0.48	0.03	< 0.01	↑
P68133	Actin. alpha skeletal muscle	180	1.60	0.47	0.09	< 0.01	↑
P68032	Actin. alpha cardiac muscle 1	180	1.58	0.46	0.03	< 0.01	↑
P0DMV8	Heat shock 70 kDa protein 1A	151	1.57	0.45	0.12	< 0.01	↑
Q6S8J3	POTE ankyrin domain family member E	62	1.57	0.45	0.10	< 0.01	↑
P02679	Fibrinogen gamma chain	189	1.55	0.44	0.14	0.01	↑
Q96DA0	Zymogen granule protein 16 homolog B	1714	1.55	0.44	0.03	< 0.01	↑
P01861	Immunoglobulin heavy constant gamma 4	125	1.54	0.43	0.06	< 0.01	↑
P60709	Actin. cytoplasmic 1	277	1.49	0.40	0.03	< 0.01	↑
P0DMV9	Heat shock 70 kDa protein 1B	150	1.48	0.39	0.13	< 0.01	↑
P62736	Actin. aortic smooth muscle	180	1.40	0.34	0.04	< 0.01	↑
P01833	Polymeric immunoglobulin receptor	2106	1.40	0.34	0.02	< 0.01	↑
P59666	Neutrophil defensin 3	271	1.36	0.31	0.10	< 0.01	↑
P68871	Hemoglobin subunit beta	4166	1.34	0.29	0.04	< 0.01	↑
P01023	Alpha-2-macroglobulin	50	1.31	0.27	0.08	< 0.01	↑
P59665	Neutrophil defensin 1	271	1.31	0.27	0.10	0.02	↑
P02042	Hemoglobin subunit delta	1039	1.30	0.26	0.07	< 0.01	↑
P12273	Prolactin-inducible protein	2680	1.28	0.25	0.02	< 0.01	↑
P63261	Actin. cytoplasmic 2	272	1.25	0.22	0.04	< 0.01	↑
P23280	Carbonic anhydrase 6	83	1.22	0.20	0.04	< 0.01	↑
P69905	Hemoglobin subunit alpha	1983	1.22	0.20	0.08	0.01	↑
P01860	Immunoglobulin heavy constant gamma 3	559	1.17	0.16	0.08	0.03	↑
P05109	Protein S100-A8	8350	1.16	0.15	0.07	0.02	↑
P06733	Alpha-enolase	317	1.13	0.12	0.06	0.03	↑
P01857	Immunoglobulin heavy constant gamma 1	2147	1.13	0.12	0.06	0.04	↑
P01876	Immunoglobulin heavy constant alpha 1	1507	1.12	0.11	0.01	< 0.01	↑
P0DOX2	Immunoglobulin alpha-2 heavy chain	429	1.08	0.08	0.01	< 0.01	↑
P01877	Immunoglobulin heavy constant alpha 2	490	1.08	0.08	0.01	< 0.01	↑
P04746	Pancreatic alpha-amylase	6383	1.03	0.03	0.02	0.02	↑
P0DUB6	Alpha-amylase 1A	8980	0.98	-0.02	0.01	0.01	↓
P0DTE7	Alpha-amylase 1B	8980	0.90	-0.10	0.01	< 0.01	↓
P0DOX7	Immunoglobulin kappa light chain	1094	0.86	-0.15	0.05	0.01	↓
P28325	Cystatin-D	540	0.79	-0.24	0.07	< 0.01	↓
P01036	Cystatin-S	1382	0.77	-0.26	0.02	< 0.01	↓
P61626	Lysozyme C	150	0.76	-0.27	0.05	< 0.01	↓
P09228	Cystatin-SA	500	0.76	-0.28	0.02	< 0.01	↓
P0DOY3	Immunoglobulin lambda constant 3	340	0.76	-0.28	0.06	< 0.01	↓
A0A075B6S6	Immunoglobulin kappa variable 2D-30	231	0.75	-0.29	0.15	0.04	↓
P06310	Immunoglobulin kappa variable 2-30	231	0.73	-0.31	0.17	0.03	↓
P02810	Salivary acidic proline-rich phosphoprotein 1/2	659	0.73	-0.31	0.04	< 0.01	↓

Q9BXT4	Tudor domain-containing protein 1	33	0.73	-0.31	0.02	< 0.01	↓
P01034	Cystatin-C	147	0.72	-0.33	0.05	< 0.01	↓
A0A0A0MRZ7	Immunoglobulin kappa variable 2D-26	231	0.71	-0.34	0.19	0.04	↓
P01614	Immunoglobulin kappa variable 2D-40	231	0.71	-0.34	0.18	0.04	↓
P10599	Thioredoxin	152	0.70	-0.36	0.10	< 0.01	↓
P04080	Cystatin-B	2200	0.69	-0.37	0.04	< 0.01	↓
P09211	Glutathione S-transferase P	85	0.66	-0.42	0.13	0.01	↓
Q96DR5	BPI fold-containing family A member 2	193	0.65	-0.43	0.04	< 0.01	↓
P00738	Haptoglobin	699	0.59	-0.52	0.10	< 0.01	↓
P00739	Haptoglobin-related protein	86	0.59	-0.53	0.15	< 0.01	↓
P22079	Lactoperoxidase	28	0.58	-0.54	0.04	< 0.01	↓
P0DOX5	Immunoglobulin gamma-1 heavy chain	2147	0.55	-0.60	0.05	< 0.01	↓
P01834	Immunoglobulin kappa constant	3629	0.53	-0.63	0.03	< 0.01	↓
P15516	Histatin-3	3202	0.53	-0.64	0.12	< 0.01	↓
B9A064	Immunoglobulin lambda-like polypeptide 5	164	0.53	-0.64	0.11	< 0.01	↓
**P0DOX8**	**Immunoglobulin lambda-1 light chain**	**164**	**0.50**	**-0.69**	**0.09**	**< 0.01**	↓
**P01037**	**Cystatin-SN**	**2007**	**0.49**	**-0.71**	**0.01**	**< 0.01**	↓
**P0CG04**	**Immunoglobulin lambda constant 1**	**164**	**0.48**	**-0.73**	**0.12**	**< 0.01**	↓
**P02768**	**Albumin**	**5626**	**0.44**	**-0.82**	**0.02**	**< 0.01**	↓
**Q6P5S2**	**Protein LEG1 homolog**	**297**	**0.38**	**-0.97**	**0.35**	**0.01**	↓
**Q01469**	**Fatty acid-binding protein 5**	**582**	**0.36**	**-1.03**	**0.37**	**0.04**	↓
**Q8TAX7**	**Mucin-7**	**62**	**0.35**	**-1.04**	**0.05**	**< 0.01**	↓
**P02808**	**Statherin**	**2512**	**0.31**	**-1.18**	**0.11**	**< 0.01**	↓
**P0DOX6**	**Immunoglobulin mu heavy chain**	**36**	**0.29**	**-1.24**	**0.07**	**< 0.01**	↓
**P01871**	**Immunoglobulin heavy constant mu**	**36**	**0.29**	**-1.25**	**0.07**	**< 0.01**	↓
**P06702**	**Protein S100-A9**	**2638**	**0.27**	**-1.31**	**0.04**	**< 0.01**	↓
**P61769**	**Beta-2-microglobulin**	**654**	**0.16**	**-1.86**	**0.04**	**< 0.01**	↓
P52209	6-phosphogluconate dehydrogenase. decarboxylating	159	-	-	-	-	T1
P02765	Alpha-2-HS-glycoprotein	579	-	-	-	-	T1
P01019	Angiotensinogen	193	-	-	-	-	T1
P03973	Antileukoproteinase	459	-	-	-	-	T1
O15078	Centrosomal protein of 290 kDa	162	-	-	-	-	T1
P00450	Ceruloplasmin	882	-	-	-	-	T1
P10909	Clusterin	191	-	-	-	-	T1
P23528	Cofilin-1	930	-	-	-	-	T1
Q9Y281	Cofilin-2	139	-	-	-	-	T1
A2RUR9	Coiled-coil domain-containing protein 144A	52	-	-	-	-	T1
Q3MJ40	Coiled-coil domain-containing protein 144B	45	-	-	-	-	T1
P0C0L4	Complement C4-A	72	-	-	-	-	T1
P0C0L5	Complement C4-B	70	-	-	-	-	T1
P54108	Cysteine-rich secretory protein 3	245	-	-	-	-	T1
Q02487	Desmocollin-2	76	-	-	-	-	T1
P02671	Fibrinogen alpha chain	360	-	-	-	-	T1
P04075	Fructose-bisphosphate aldolase A	177	-	-	-	-	T1

P06744	Glucose-6-phosphate isomerase	436	-	-	-	-	T1
Q92993	Histone acetyltransferase KAT5	157	-	-	-	-	T1
P18510	Interleukin-1 receptor antagonist protein	241	-	-	-	-	T1
P06870	Kallikrein-1	107	-	-	-	-	T1
O14782	Kinesin-like protein KIF3C	52	-	-	-	-	T1
P00338	L-lactate dehydrogenase A chain	877	-	-	-	-	T1
Q6ZMR3	L-lactate dehydrogenase A-like 6A	351	-	-	-	-	T1
P07195	L-lactate dehydrogenase B chain	325	-	-	-	-	T1
P07864	L-lactate dehydrogenase C chain	325	-	-	-	-	T1
O95274	Ly6/PLAUR domain-containing protein 3	144	-	-	-	-	T1
O60449	Lymphocyte antigen 75	18	-	-	-	-	T1
P40925	Malate dehydrogenase. cytoplasmic	117	-	-	-	-	T1
O75556	Mammaglobin-B	522	-	-	-	-	T1
P14780	Matrix metalloproteinase-9	179	-	-	-	-	T1
O00255	Menin	47	-	-	-	-	T1
Q8NEM0	Microcephalin	50	-	-	-	-	T1
O43318	Mitogen-activated protein kinase kinase kinase 7	42	-	-	-	-	T1
P26038	Moesin	110	-	-	-	-	T1
P24158	Myeloblastin	379	-	-	-	-	T1
P05164	Myeloperoxidase	87	-	-	-	-	T1
O75161	Nephrocystin-4	62	-	-	-	-	T1
P80188	Neutrophil gelatinase-associated lipocalin	2719	-	-	-	-	T1
Q9Y536	Peptidyl-prolyl cis-trans isomerase A-like 4A	40	-	-	-	-	T1
P30041	Peroxiredoxin-6	153	-	-	-	-	T1
P00558	Phosphoglycerate kinase 1	35	-	-	-	-	T1
P07205	Phosphoglycerate kinase 2	127	-	-	-	-	T1
P13797	Plastin-3	34	-	-	-	-	T1
Q8TBY8	Polyamine-modulated factor 1-binding protein 1	45	-	-	-	-	T1
Q16378	Proline-rich protein 4	903	-	-	-	-	T1
P07602	Prosaposin	96	-	-	-	-	T1
P02760	Protein AMBP	149	-	-	-	-	T1
P07237	Protein disulfide-isomerase	180	-	-	-	-	T1
Q6NUI1	Putative coiled-coil domain-containing protein 144 N-terminal-like	39	-	-	-	-	T1
Q8IYA2	Putative coiled-coil domain-containing protein 144C	50	-	-	-	-	T1
P35241	Radixin	88	-	-	-	-	T1
P35249	Replication factor C subunit 4	66	-	-	-	-	T1
P52566	Rho GDP-dissociation inhibitor 2	206	-	-	-	-	T1
Q8N392	Rho GTPase-activating protein 18	59	-	-	-	-	T1
Q96QB1	Rho GTPase-activating protein 7	85	-	-	-	-	T1
Q9H299	SH3 domain-binding glutamic acid-rich-like protein 3	413	-	-	-	-	T1
Q9NTJ3	Structural maintenance of chromosomes protein 4	46	-	-	-	-	T1
Q6PKC3	Thioredoxin domain-containing protein 11	17	-	-	-	-	T1

Q9Y4F4	TOG array regulator of axonemal microtubules protein 1	52	-	-	-	-	T1
P20061	Transcobalamin-1	127	-	-	-	-	T1
P02766	Transthyretin	255	-	-	-	-	T1
P60174	Triosephosphate isomerase	520	-	-	-	-	T1
P36941	Tumor necrosis factor receptor superfamily member 3	87	-	-	-	-	T1
O94966	Ubiquitin carboxyl-terminal hydrolase 19	20	-	-	-	-	T1
P02774	Vitamin D-binding protein	1144	-	-	-	-	T1
P04004	Vitronectin	65	-	-	-	-	T1
Q96KN7	X-linked retinitis pigmentosa GTPase regulator-interacting protein 1	61	-	-	-	-	T1
Q63HK3	Zinc finger protein with KRAB and SCAN domains 2	43	-	-	-	-	T1
Q8NHQ9	ATP-dependent RNA helicase DDX55	906	-	-	-	-	T2
P27482	Calmodulin-like protein 3	121	-	-	-	-	T2
P33991	DNA replication licensing factor MCM4	19	-	-	-	-	T2
P32519	ETS-related transcription factor Elf-1	27	-	-	-	-	T2
Q08380	Galectin-3-binding protein	15	-	-	-	-	T2
Q969F9	Hermansky-Pudlak syndrome 3 protein	29	-	-	-	-	T2
P15515	Histatin-1	2908	-	-	-	-	T2
Q9NR48	Histone-lysine N-methyltransferase ASH1L	17	-	-	-	-	T2
Q2TBA0	Kelch-like protein 40	41	-	-	-	-	T2
Q9BUT9	MAPK regulated corepressor interacting protein 2	42	-	-	-	-	T2
Q02817	Mucin-2	33	-	-	-	-	T2
A8MUU1	Putative fatty acid-binding protein 5-like protein 3	227	-	-	-	-	T2
Q9NSD5	Sodium- and chloride-dependent GABA transporter 2	141	-	-	-	-	T2
O60264	SWI/SNF-related matrix-associated actin-dependent regulator of chromatin subfamily A member 5	14	-	-	-	-	T2
Q5JTD0	Tight junction-associated protein 1	19	-	-	-	-	T2
Q6ZVM7	TOM1-like protein 2	66	-	-	-	-	T2
Q502W6	von Willebrand factor A domain-containing protein 3B	65	-	-	-	-	T2
Q5SW79	Centrosomal protein of 170 kDa	150	1.68	0.52	0.66	0.71	SE
P06396	Gelsolin	68	1.42	0.35	0.26	0.86	SE
Q01518	Adenylyl cyclase-associated protein 1	208	1.30	0.26	0.20	0.89	SE
P62937	Peptidyl-prolyl cis-trans isomerase A	138	1.27	0.24	0.20	0.86	SE
P09104	Gamma-enolase	55	1.21	0.19	0.23	0.83	SE
P01859	Immunoglobulin heavy constant gamma 2	98	1.19	0.17	0.09	0.94	SE
P48741	Putative heat shock 70 kDa protein 7	87	1.13	0.12	0.22	0.67	SE
P17066	Heat shock 70 kDa protein 6	87	1.08	0.08	0.20	0.64	SE
P11142	Heat shock cognate 71 kDa protein	87	1.08	0.08	0.18	0.66	SE
P54652	Heat shock-related 70 kDa protein 2	87	1.08	0.08	0.18	0.63	SE
P0DTE8	Alpha-amylase 1C	8980	1.04	0.04	0.02	0.92	SE

P19961	Alpha-amylase 2B	7498	1.02	0.02	0.01	0.94	SE
P02763	Alpha-1-acid glycoprotein 1	116	0.99	-0.01	0.19	0.52	SE
P30613	Pyruvate kinase PKLR	65	0.92	-0.08	0.48	0.51	SE
P11021	Endoplasmic reticulum chaperone BiP	57	0.90	-0.11	0.29	0.36	SE
P31025	Lipocalin-1	2310	0.90	-0.11	0.06	0.05	SE
P07108	Acyl-CoA-binding protein	235	0.89	-0.12	0.16	0.28	SE
P13929	Beta-enolase	55	0.85	-0.16	0.22	0.22	SE
P69891	Hemoglobin subunit gamma-1	932	0.80	-0.22	0.28	0.46	SE
P69892	Hemoglobin subunit gamma-2	932	0.79	-0.23	0.55	0.51	SE
A0A087WW87	Immunoglobulin kappa variable 2-40	231	0.75	-0.29	0.22	0.11	SE
A2NJV5	Immunoglobulin kappa variable 2-29	231	0.74	-0.30	0.19	0.11	SE
P01615	Immunoglobulin kappa variable 2D-28	231	0.74	-0.30	0.21	0.06	SE
A0A075B6S2	Immunoglobulin kappa variable 2D-29	231	0.74	-0.30	0.21	0.05	SE
A0A075B6P5	Immunoglobulin kappa variable 2-28	231	0.73	-0.31	0.20	0.07	SE
A8K2U0	Alpha-2-macroglobulin-like protein 1	33	0.73	-0.32	0.32	0.20	SE
P02100	Hemoglobin subunit epsilon	932	0.70	-0.36	0.54	0.38	SE
P20742	Pregnancy zone protein	91	0.67	-0.40	0.28	0.16	SE

Note: Ratio T1/T2 (fold change) = ratio between pregnancy (T1) and after delivery (T2) for OWP; Log(e) ("e" is a constant = 2.71); SD, standard deviation; *p*, statistical significance (adjusted by False Discovery Rate–FDR = 4); ↑ = up-regulated (1-$p > 0.95$); ↓ = down-regulated ($p < 0.05$); SE = similar expression compared to T2; bold lines refer to up- or down-regulated proteins by more than 2-fold

S3–Table C. Proteins identified in saliva from NP and their differences in expression during T1 and T2

Accession number	Protein name	Score	Ratio T1/T2	Log(e)	SD	*p*	Expression differences
**P05109**	**Protein S100-A8**	**8263**	**8.33**	**2.12**	**0.02**	**< 0.01**	**↑**
**P02100**	**Hemoglobin subunit epsilon**	**748**	**7.32**	**1.99**	**0.06**	**< 0.01**	**↑**
**P06744**	**Glucose-6-phosphate isomerase**	**141**	**5.64**	**1.73**	**0.15**	**< 0.01**	**↑**
**Q01518**	**Adenylyl cyclase-associated protein 1**	**201**	**4.62**	**1.53**	**0.18**	**< 0.01**	**↑**
**P15515**	**Histatin-1**	**588**	**4.39**	**1.48**	**0.26**	**< 0.01**	**↑**
**P00338**	**L-lactate dehydrogenase A chain**	**161**	**3.03**	**1.11**	**0.21**	**< 0.01**	**↑**
**Q9UGM3**	**Deleted in malignant brain tumors 1 protein**	**218**	**3.00**	**1.10**	**0.05**	**< 0.01**	**↑**
**P0DUB6**	**Alpha-amylase 1A**	**24717**	**2.56**	**0.94**	**0.01**	**< 0.01**	**↑**
**P19961**	**Alpha-amylase 2B**	**20514**	**2.56**	**0.94**	**0.01**	**< 0.01**	**↑**
**P0DTE7**	**Alpha-amylase 1B**	**24717**	**2.48**	**0.91**	**0.01**	**< 0.01**	**↑**
**Q9UBC9**	**Small proline-rich protein 3**	**367**	**2.48**	**0.91**	**0.06**	**< 0.01**	**↑**
**P0DTE8**	**Alpha-amylase 1C**	**24717**	**2.46**	**0.90**	**0.01**	**< 0.01**	**↑**
**P60709**	**Actin. cytoplasmic 1**	**11816**	**2.23**	**0.80**	**0.02**	**< 0.01**	**↑**
**P04746**	**Pancreatic alpha-amylase**	**15692**	**2.08**	**0.73**	**0.01**	**< 0.01**	**↑**
**P07737**	**Profilin-1**	**2737**	**2.03**	**0.71**	**0.04**	**< 0.01**	**↑**
Q562R1	Beta-actin-like protein 2	2076	1.88	0.63	0.05	< 0.01	↑
P62736	Actin. aortic smooth muscle	5303	1.79	0.58	0.04	< 0.01	↑
P63261	Actin. cytoplasmic 2	11816	1.73	0.55	0.03	< 0.01	↑

P63267	Actin. gamma-enteric smooth muscle	5281	1.70	0.53	0.03	< 0.01	↑
P68032	Actin. alpha cardiac muscle 1	5299	1.70	0.53	0.02	< 0.01	↑
P68133	Actin. alpha skeletal muscle	5299	1.70	0.53	0.03	< 0.01	↑
Q6S8J3	POTE ankyrin domain family member E	2018	1.70	0.53	0.05	< 0.01	↑
Q9BYX7	Putative beta-actin-like protein 3	1077	1.70	0.53	0.06	< 0.01	↑
A5A3E0	POTE ankyrin domain family member F	1926	1.60	0.47	0.06	< 0.01	↑
P04075	Fructose-bisphosphate aldolase A	141	1.60	0.47	0.13	< 0.01	↑
P30613	Pyruvate kinase PKLR	50	1.49	0.40	0.16	0.02	↑
P37837	Transaldolase	168	1.43	0.36	0.12	0.01	↑
P0DMV8	Heat shock 70 kDa protein 1A	142	1.35	0.30	0.13	0.01	↑
P13796	Plastin-2	262	1.25	0.22	0.12	0.03	↑
P0CG38	POTE ankyrin domain family member I	1323	1.19	0.17	0.04	0.01	↑
P06733	Alpha-enolase	920	1.17	0.16	0.04	< 0.01	↑
P01037	Cystatin-SN	9122	1.14	0.13	0.03	< 0.01	↑
P0CG39	POTE ankyrin domain family member J	995	1.12	0.11	0.06	0.02	↑
P06702	Protein S100-A9	13909	0.87	-0.14	0.03	< 0.01	↓
P02810	Salivary acidic proline-rich phosphoprotein 1/2	3280	0.82	-0.20	0.06	< 0.01	↓
P23280	Carbonic anhydrase 6	221	0.79	-0.23	0.08	< 0.01	↓
P09228	Cystatin-SA	3667	0.79	-0.24	0.05	< 0.01	↓
P31025	Lipocalin-1	3571	0.79	-0.24	0.05	< 0.01	↓
Q96DR5	BPI fold-containing family A member 2	436	0.79	-0.24	0.10	< 0.01	↓
P69892	Hemoglobin subunit gamma-2	748	0.76	-0.27	0.13	0.02	↓
A0M8Q6	Immunoglobulin lambda constant 7	741	0.75	-0.29	0.13	< 0.01	↓
P69891	Hemoglobin subunit gamma-1	748	0.75	-0.29	0.10	0.01	↓
P02812	Basic salivary proline-rich protein 2	2639	0.72	-0.33	0.15	0.02	↓
P29401	Transketolase	119	0.70	-0.35	0.11	0.01	↓
P0DOY3	Immunoglobulin lambda constant 3	2811	0.70	-0.36	0.06	< 0.01	↓
P52566	Rho GDP-dissociation inhibitor 2	252	0.70	-0.36	0.13	0.01	↓
P0CF74	Immunoglobulin lambda constant 6	2577	0.68	-0.39	0.05	< 0.01	↓
B9A064	Immunoglobulin lambda-like polypeptide 5	1892	0.66	-0.42	0.08	< 0.01	↓
P0DOY2	Immunoglobulin lambda constant 2	2811	0.65	-0.43	0.07	< 0.01	↓
P01876	Immunoglobulin heavy constant alpha 1	12347	0.64	-0.44	0.02	< 0.01	↓
P09211	Glutathione S-transferase P	267	0.64	-0.44	0.11	< 0.01	↓
Q8N4F0	BPI fold-containing family B member 2	197	0.64	-0.44	0.08	< 0.01	↓
P0DOX2	Immunoglobulin alpha-2 heavy chain	4357	0.63	-0.46	0.03	< 0.01	↓
P01877	Immunoglobulin heavy constant alpha 2	4619	0.63	-0.47	0.02	< 0.01	↓
P0DOX5	Immunoglobulin gamma-1 heavy chain	5720	0.63	-0.47	0.06	< 0.01	↓
P01861	Immunoglobulin heavy constant gamma 4	675	0.61	-0.49	0.05	< 0.01	↓
P22079	Lactoperoxidase	141	0.61	-0.49	0.14	< 0.01	↓
P01591	Immunoglobulin J chain	1967	0.60	-0.51	0.06	< 0.01	↓
P01023	Alpha-2-macroglobulin	144	0.59	-0.52	0.06	< 0.01	↓
P02768	Albumin	24819	0.59	-0.52	0.01	< 0.01	↓
P01860	Immunoglobulin heavy constant gamma 3	986	0.59	-0.53	0.05	< 0.01	↓
P02790	Hemopexin	187	0.59	-0.53	0.10	< 0.01	↓
Q96DA0	Zymogen granule protein 16 homolog B	6029	0.53	-0.63	0.03	< 0.01	↓

P01034	Cystatin-C	289	0.52	-0.66	0.07	< 0.01	↓
P02647	Apolipoprotein A-I	1550	0.52	-0.66	0.09	< 0.01	↓
P01024	Complement C3	94	0.51	-0.67	0.10	< 0.01	↓
**P01833**	**Polymeric immunoglobulin receptor**	**3752**	**0.49**	**-0.71**	**0.02**	**< 0.01**	↓
**P06396**	**Gelsolin**	**126**	**0.49**	**-0.72**	**0.26**	**< 0.01**	↓
**P01834**	**Immunoglobulin kappa constant**	**5873**	**0.48**	**-0.74**	**0.05**	**< 0.01**	↓
**P28325**	**Cystatin-D**	**181**	**0.48**	**-0.74**	**0.05**	**< 0.01**	↓
**P02814**	**Submaxillary gland androgen-regulated protein 3B**	**23046**	**0.47**	**-0.75**	**0.02**	**< 0.01**	↓
**P07205**	**Phosphoglycerate kinase 2**	**70**	**0.47**	**-0.76**	**0.32**	**0.02**	↓
**P02787**	**Serotransferrin**	**1159**	**0.45**	**-0.79**	**0.03**	**< 0.01**	↓
**P0DOX7**	**Immunoglobulin kappa light chain**	**2177**	**0.43**	**-0.84**	**0.05**	**< 0.01**	↓
**P68871**	**Hemoglobin subunit beta**	**4087**	**0.37**	**-1.00**	**0.03**	**< 0.01**	↓
**P01009**	**Alpha-1-antitrypsin**	**70**	**0.34**	**-1.09**	**0.10**	**< 0.01**	↓
**P01857**	**Immunoglobulin heavy constant gamma 1**	**5729**	**0.31**	**-1.17**	**0.05**	**< 0.01**	↓
**P59665**	**Neutrophil defensin 1**	**3782**	**0.30**	**-1.20**	**0.11**	**< 0.01**	↓
**P0DOX8**	**Immunoglobulin lambda-1 light chain**	**1892**	**0.28**	**-1.26**	**0.08**	**< 0.01**	↓
**P0CG04**	**Immunoglobulin lambda constant 1**	**1892**	**0.28**	**-1.29**	**0.05**	**< 0.01**	↓
**P01859**	**Immunoglobulin heavy constant gamma 2**	**583**	**0.27**	**-1.32**	**0.09**	**< 0.01**	↓
**P04080**	**Cystatin-B**	**975**	**0.25**	**-1.37**	**0.04**	**< 0.01**	↓
**P02042**	**Hemoglobin subunit delta**	**976**	**0.23**	**-1.48**	**0.03**	**< 0.01**	↓
**P03973**	**Antileukoproteinase**	**482**	**0.22**	**-1.50**	**0.09**	**< 0.01**	↓
**P61626**	**Lysozyme C**	**3594**	**0.21**	**-1.56**	**0.04**	**< 0.01**	↓
**P00739**	**Haptoglobin-related protein**	**19**	**0.21**	**-1.57**	**0.23**	**< 0.01**	↓
**Q5T7N2**	**LINE-1 type transposase domain-containing protein 1**	**30**	**0.21**	**-1.57**	**0.03**	**< 0.01**	↓
**P59666**	**Neutrophil defensin 3**	**3782**	**0.20**	**-1.59**	**0.04**	**< 0.01**	↓
**P00738**	**Haptoglobin**	**184**	**0.18**	**-1.73**	**0.04**	**< 0.01**	↓
**P69905**	**Hemoglobin subunit alpha**	**2143**	**0.14**	**-1.94**	**0.01**	**< 0.01**	↓
**P02008**	**Hemoglobin subunit zeta**	**241**	**0.08**	**-2.53**	**0.10**	**0.01**	↓
**P61769**	**Beta-2-microglobulin**	**576**	**0.07**	**-2.60**	**0.06**	**< 0.01**	↓
P07108	Acyl-CoA-binding protein	195	-	-	-	-	T1
A8K2U0	Alpha-2-macroglobulin-like protein 1	38	-	-	-	-	T1
P27216	Annexin A13	392	-	-	-	-	T1
Q8TDL5	BPI fold-containing family B member 1	86	-	-	-	-	T1
P0DP23	Calmodulin-1	252	-	-	-	-	T1
P0DP24	Calmodulin-2	252	-	-	-	-	T1
P0DP25	Calmodulin-3	252	-	-	-	-	T1
Q8NEL0	Coiled-coil domain-containing protein 54	185	-	-	-	-	T1
Q9UBG3	Cornulin	258	-	-	-	-	T1
Q99543	DnaJ homolog subfamily C member 2	51	-	-	-	-	T1
P11021	Endoplasmic reticulum chaperone BiP	30	-	-	-	-	T1
P15311	Ezrin	20	-	-	-	-	T1
Q9P2Q2	FERM domain-containing protein 4A	24	-	-	-	-	T1
Q5RHP9	Glutamate-rich protein 3	41	-	-	-	-	T1

Q9UJ14	Glutathione hydrolase 7	64	-	-	-	-	T1
O14556	Glyceraldehyde-3-phosphate dehydrogenase. testis-specific	93	-	-	-	-	T1
Q8NBJ4	Golgi membrane protein 1	63	-	-	-	-	T1
P17066	Heat shock 70 kDa protein 6	52	-	-	-	-	T1
P11142	Heat shock cognate 71 kDa protein	67	-	-	-	-	T1
P54652	Heat shock-related 70 kDa protein 2	58	-	-	-	-	T1
P01764	Immunoglobulin heavy variable 3-23	928	-	-	-	-	T1
P01768	Immunoglobulin heavy variable 3-30	928	-	-	-	-	T1
P0DP02	Immunoglobulin heavy variable 3-30-3	928	-	-	-	-	T1
P0DP03	Immunoglobulin heavy variable 3-30-5	928	-	-	-	-	T1
P01772	Immunoglobulin heavy variable 3-33	928	-	-	-	-	T1
P01767	Immunoglobulin heavy variable 3-53	928	-	-	-	-	T1
A0A0C4DH42	Immunoglobulin heavy variable 3-66	928	-	-	-	-	T1
A0A0B4J1X5	Immunoglobulin heavy variable 3-74	928	-	-	-	-	T1
P04433	Immunoglobulin kappa variable 3-11	578	-	-	-	-	T1
A0A0A0MRZ8	Immunoglobulin kappa variable 3D-11	578	-	-	-	-	T1
Q6ZMR3	L-lactate dehydrogenase A-like 6A	56	-	-	-	-	T1
P07195	L-lactate dehydrogenase B chain	56	-	-	-	-	T1
P07864	L-lactate dehydrogenase C chain	56	-	-	-	-	T1
Q6B0I6	Lysine-specific demethylase 4D	104	-	-	-	-	T1
P14780	Matrix metalloproteinase-9	82	-	-	-	-	T1
P26038	Moesin	50	-	-	-	-	T1
Q02817	Mucin-2	20	-	-	-	-	T1
P47874	Olfactory marker protein	102	-	-	-	-	T1
Q14651	Plastin-1	33	-	-	-	-	T1
P13797	Plastin-3	86	-	-	-	-	T1
Q9Y2S7	Polymerase delta-interacting protein 2	70	-	-	-	-	T1
Q9UQ80	Proliferation-associated protein 2G4	41	-	-	-	-	T1
Q6MZM9	Proline-rich protein 27	248	-	-	-	-	T1
Q16378	Proline-rich protein 4	984	-	-	-	-	T1
P80511	Protein S100-A12	209	-	-	-	-	T1
P48741	Putative heat shock 70 kDa protein 7	52	-	-	-	-	T1
P35241	Radixin	19	-	-	-	-	T1
Q9H299	SH3 domain-binding glutamic acid-rich-like protein	354	-	-	-	-	T1
P35326	Small proline-rich protein 2A	526	-	-	-	-	T1
P35325	Small proline-rich protein 2B	245	-	-	-	-	T1
P22532	Small proline-rich protein 2D	245	-	-	-	-	T1
P22531	Small proline-rich protein 2E	245	-	-	-	-	T1
Q9BYE4	Small proline-rich protein 2G	245	-	-	-	-	T1
Q6UWP8	Suprabasin	60	-	-	-	-	T1
P26639	Threonine--tRNA ligase 1. cytoplasmic	92	-	-	-	-	T1
Q9Y4F4	TOG array regulator of axonemal microtubules protein 1	97	-	-	-	-	T1
P51809	Vesicle-associated membrane protein 7	100	-	-	-	-	T1

P63104	14-3-3 protein zeta/delta	239	-	-	-	-	T2
P02763	Alpha-1-acid glycoprotein 1	238	-	-	-	-	T2
P02765	Alpha-2-HS-glycoprotein	143	-	-	-	-	T2
O43707	Alpha-actinin-4	53	-	-	-	-	T2
Q66GS9	Centrosomal protein of 135 kDa	61	-	-	-	-	T2
O95196	Chondroitin sulfate proteoglycan 5	79	-	-	-	-	T2
Q01469	Fatty acid-binding protein 5	476	-	-	-	-	T2
Q5W0V3	FHF complex subunit HOOK interacting protein 2A	78	-	-	-	-	T2
Q2TBA0	Kelch-like protein 40	26	-	-	-	-	T2
Q9BXW6	Oxysterol-binding protein-related protein 1	50	-	-	-	-	T2
Q9Y536	Peptidyl-prolyl cis-trans isomerase A-like 4A	353	-	-	-	-	T2
Q6UXT9	Protein ABHD15	61	-	-	-	-	T2
Q9BVG4	Protein PBDC1	36	-	-	-	-	T2
Q96LQ0	Protein phosphatase 1 regulatory subunit 36	77	-	-	-	-	T2
Q9C0D5	Protein TANC1	28	-	-	-	-	T2
Q8NCN5	Pyruvate dehydrogenase phosphatase regulatory subunit. mitochondrial	30	-	-	-	-	T2
Q99757	Thioredoxin. mitochondrial	95	-	-	-	-	T2
Q6ZQQ6	WD repeat-containing protein 87	10	-	-	-	-	T2
P25311	Zinc-alpha-2-glycoprotein	356	-	-	-	-	T2
A0A087WW87	Immunoglobulin kappa variable 2-40	201	1.38	0.32	0.28	0.84	SE
P01614	Immunoglobulin kappa variable 2D-40	201	1.38	0.32	0.26	0.84	SE
A0A075B6S2	Immunoglobulin kappa variable 2D-29	201	1.35	0.30	0.24	0.80	SE
P02808	Statherin	5950	1.34	0.29	0.80	0.57	SE
P06310	Immunoglobulin kappa variable 2-30	201	1.34	0.29	0.33	0.81	SE
A0A075B6S6	Immunoglobulin kappa variable 2D-30	201	1.32	0.28	0.26	0.80	SE
P01615	Immunoglobulin kappa variable 2D-28	201	1.32	0.28	0.26	0.76	SE
A0A0A0MRZ7	Immunoglobulin kappa variable 2D-26	201	1.30	0.26	0.31	0.82	SE
A2NJV5	Immunoglobulin kappa variable 2-29	201	1.27	0.24	0.27	0.71	SE
P13929	Beta-enolase	226	1.26	0.23	0.15	0.95	SE
A0A075B6P5	Immunoglobulin kappa variable 2-28	201	1.25	0.22	0.26	0.72	SE
Q6P5S2	Protein LEG1 homolog	336	1.22	0.20	0.18	0.79	SE
P0DMV9	Heat shock 70 kDa protein 1B	135	1.21	0.19	0.17	0.86	SE
P54108	Cysteine-rich secretory protein 3	55	1.14	0.13	0.19	0.73	SE
P0DOX6	Immunoglobulin mu heavy chain	71	1.12	0.11	0.07	0.94	SE
P02774	Vitamin D-binding protein	85	1.09	0.09	0.22	0.62	SE
P02788	Lactotransferrin	320	1.07	0.07	0.11	0.70	SE
Q14508	WAP four-disulfide core domain protein 2	151	1.06	0.06	0.22	0.55	SE
P04406	Glyceraldehyde-3-phosphate dehydrogenase	868	1.05	0.05	0.13	0.61	SE
P14618	Pyruvate kinase PKM	616	1.05	0.05	0.09	0.69	SE
P80188	Neutrophil gelatinase-associated lipocalin	1032	1.05	0.05	0.17	0.61	SE
P62937	Peptidyl-prolyl cis-trans isomerase A	508	1.04	0.04	0.15	0.60	SE
P12273	Prolactin-inducible protein	12382	1.02	0.02	0.06	0.68	SE
P01036	Cystatin-S	9562	1.01	0.01	0.03	0.64	SE

P34931	Heat shock 70 kDa protein 1-like	119	1.01	0.01	0.21	0.52	SE
P24158	Myeloblastin	84	1.00	0	0.23	0.50	SE
P10599	Thioredoxin	152	0.96	-0.04	0.17	0.41	SE
Q8TAX7	Mucin-7	1008	0.94	-0.06	0.05	0.11	SE
Q8NHQ9	ATP-dependent RNA helicase DDX55	186	0.93	-0.07	0.61	0.47	SE
P02679	Fibrinogen gamma chain	418	0.90	-0.10	0.12	0.16	SE
Q5VSP4	Putative lipocalin 1-like protein 1	3437	0.84	-0.17	0.12	0.09	SE
P02675	Fibrinogen beta chain	394	0.82	-0.20	0.15	0.08	SE
P52209	6-phosphogluconate dehydrogenase. decarboxylating	116	0.82	-0.20	0.13	0.07	SE
P09104	Gamma-enolase	152	0.78	-0.25	0.23	0.10	SE
P15516	Histatin-3	1761	0.77	-0.26	0.68	0.54	SE
P01871	Immunoglobulin heavy constant mu	71	0.73	-0.31	0.24	0.13	SE
P00558	Phosphoglycerate kinase 1	62	0.61	-0.49	0.27	0.06	SE
P04280	Basic salivary proline-rich protein 1	1572	0.44	-0.82	0.39	0.11	SE

Note: Ratio T1/T2 (fold change) = ratio between pregnancy (T1) and after delivery (T2) for NP; Log(e) ("e" is a constant = 2.71); SD, standard deviation; *p*, statistical significance (adjusted by False Discovery Rate–FDR = 4); ↑ = up-regulated (1-p > 0.95); ↓ = down-regulated (p < 0.05); SE = similar expression compared to T2; bold lines refer to up- or down-regulated proteins by more than 2-fold

S3–Table D. Proteins identified in saliva of NWP and their differences in expression during T1 and T2

Accession number	Protein name	Score	Ratio T1/T2	Log(e)	SD	*p*	Expression differences
**Q8TDL5**	**BPI fold-containing family B member 1**	**96**	**11.70**	**2.46**	**0.08**	**< 0.01**	↑
**P02808**	**Statherin**	**3641**	**4.26**	**1.45**	**0.37**	**0.01**	↑
**P02788**	**Lactotransferrin**	**82**	**4.10**	**1.41**	**0.13**	**< 0.01**	↑
**Q16378**	**Proline-rich protein 4**	**2753**	**3.06**	**1.12**	**0.49**	**0.04**	↑
**P02812**	**Basic salivary proline-rich protein 2**	**6851**	**2.66**	**0.98**	**0.02**	**< 0.01**	↑
**Q6S8J3**	**POTE ankyrin domain family member E**	**3652**	**2.59**	**0.95**	**0.06**	**< 0.01**	↑
**A5A3E0**	**POTE ankyrin domain family member F**	**3652**	**2.56**	**0.94**	**0.06**	**< 0.01**	↑
**P02810**	**Salivary acidic proline-rich phosphoprotein 1/2**	**2359**	**2.53**	**0.93**	**0.01**	**< 0.01**	↑
**P68133**	**Actin, alpha skeletal muscle**	**5916**	**2.14**	**0.76**	**0.07**	**< 0.01**	↑
**P63267**	**Actin, gamma-enteric smooth muscle**	**5916**	**2.05**	**0.72**	**0.06**	**< 0.01**	↑
**P62736**	**Actin, aortic smooth muscle**	**5916**	**2.01**	**0.70**	**0.05**	**< 0.01**	↑
P09228	Cystatin-SA	6385	1.97	0.68	0.02	< 0.01	↑
P01036	Cystatin-S	15763	1.90	0.64	0.02	< 0.01	↑
P63261	Actin, cytoplasmic 2	7700	1.88	0.63	0.04	< 0.01	↑
P22079	Lactoperoxidase	68	1.82	0.60	0.12	< 0.01	↑
P19961	Alpha-amylase 2B	18787	1.80	0.59	0.01	< 0.01	↑
P12273	Prolactin-inducible protein	13097	1.70	0.53	0.03	< 0.01	↑
P60709	Actin, cytoplasmic 1	7700	1.63	0.49	0.08	< 0.01	↑
P23280	Carbonic anhydrase 6	157	1.62	0.48	0.06	< 0.01	↑
Q96DA0	Zymogen granule protein 16 homolog B	6943	1.55	0.44	0.02	< 0.01	↑

Q5VSP4	Putative lipocalin 1-like protein 1	1864	1.51	0.41	0.06	< 0.01	↑
P0DTE8	Alpha-amylase 1C	21876	1.43	0.36	0.01	< 0.01	↑
P31025	Lipocalin-1	3326	1.43	0.36	0.04	< 0.01	↑
P04746	Pancreatic alpha-amylase	13239	1.39	0.33	0.02	< 0.01	↑
P0DTE7	Alpha-amylase 1B	21876	1.34	0.29	0.01	< 0.01	↑
P0DUB6	Alpha-amylase 1A	21876	1.34	0.29	0.01	< 0.01	↑
P28325	Cystatin-D	1595	1.34	0.29	0.08	< 0.01	↑
Q96DR5	BPI fold-containing family A member 2	323	1.19	0.17	0.04	< 0.01	↑
P01877	Immunoglobulin heavy constant alpha 2	3603	0.90	-0.10	0.02	< 0.01	↓
P0DOX2	Immunoglobulin alpha-2 heavy chain	3309	0.90	-0.10	0.02	< 0.01	↓
P01876	Immunoglobulin heavy constant alpha 1	7226	0.86	-0.15	0.02	< 0.01	↓
Q8N4F0	BPI fold-containing family B member 2	343	0.83	-0.19	0.08	0.01	↓
P06733	Alpha-enolase	231	0.82	-0.20	0.10	0.01	↓
P0DOX8	Immunoglobulin lambda-1 light chain	1469	0.76	-0.27	0.10	< 0.01	↓
P02647	Apolipoprotein A-I	905	0.76	-0.28	0.10	< 0.01	↓
B9A064	Immunoglobulin lambda-like polypeptide 5	1469	0.73	-0.32	0.12	< 0.01	↓
P0CG04	Immunoglobulin lambda constant 1	1469	0.72	-0.33	0.09	< 0.01	↓
P0DMV9	Heat shock 70 kDa protein 1B	240	0.69	-0.37	0.17	0.01	↓
P37837	Transaldolase	133	0.64	-0.44	0.19	< 0.01	↓
P01037	Cystatin-SN	21655	0.61	-0.50	0.04	< 0.01	↓
P0CG39	POTE ankyrin domain family member J	457	0.58	-0.54	0.17	< 0.01	↓
P02768	Albumin	15244	0.57	-0.57	0.01	< 0.01	↓
P0DOY2	Immunoglobulin lambda constant 2	1261	0.57	-0.57	0.09	< 0.01	↓
P52209	6-phosphogluconate dehydrogenase, decarboxylating	81	0.57	-0.57	0.18	< 0.01	↓
P0CF74	Immunoglobulin lambda constant 6	946	0.56	-0.58	0.07	< 0.01	↓
P0DOY3	Immunoglobulin lambda constant 3	1261	0.55	-0.59	0.09	< 0.01	↓
P0DMV8	Heat shock 70 kDa protein 1A	256	0.54	-0.61	0.22	0.01	↓
P14618	Pyruvate kinase PKM	214	0.53	-0.64	0.16	< 0.01	↓
P02787	Serotransferrin	703	0.52	-0.65	0.04	< 0.01	↓
P02814	Submaxillary gland androgen-regulated protein 3B	28616	0.52	-0.65	0.02	< 0.01	↓
P52566	Rho GDP-dissociation inhibitor 2	90	0.52	-0.66	0.21	0.01	↓
**P07737**	**Profilin-1**	**1509**	**0.50**	**-0.69**	**0.11**	**< 0.01**	↓
**Q9UGM3**	**Deleted in malignant brain tumors 1 protein**	**153**	**0.49**	**-0.71**	**0.06**	**< 0.01**	↓
**P68032**	**Actin, alpha cardiac muscle 1**	**5916**	**0.46**	**-0.77**	**0.07**	**< 0.01**	↓
**P13796**	**Plastin-2**	**295**	**0.46**	**-0.78**	**0.13**	**< 0.01**	↓
**P61769**	**Beta-2-microglobulin**	**784**	**0.45**	**-0.80**	**0.14**	**< 0.01**	↓
**Q562R1**	**Beta-actin-like protein 2**	**4453**	**0.42**	**-0.86**	**0.05**	**< 0.01**	↓
**P01857**	**Immunoglobulin heavy constant gamma 1**	**3271**	**0.41**	**-0.89**	**0.04**	**< 0.01**	↓
**P0DOX5**	**Immunoglobulin gamma-1 heavy chain**	**3271**	**0.38**	**-0.97**	**0.06**	**< 0.01**	↓
**P02790**	**Hemopexin**	**468**	**0.36**	**-1.02**	**0.12**	**< 0.01**	↓
**P05109**	**Protein S100-A8**	**110**	**0.35**	**-1.06**	**0.14**	**< 0.01**	↓
**P01023**	**Alpha-2-macroglobulin**	**91**	**0.34**	**-1.07**	**0.13**	**< 0.01**	↓
**Q9BYX7**	**Putative beta-actin-like protein 3**	**3325**	**0.33**	**-1.12**	**0.05**	**< 0.01**	↓

**P00738**	**Haptoglobin**	**192**	**0.32**	**-1.13**	**0.09**	**< 0.01**	**↓**
**P04280**	**Basic salivary proline-rich protein 1**	**5717**	**0.31**	**-1.16**	**0.04**	**< 0.01**	**↓**
**P01860**	**Immunoglobulin heavy constant gamma 3**	**377**	**0.29**	**-1.23**	**0.09**	**< 0.01**	**↓**
**P01859**	**Immunoglobulin heavy constant gamma 2**	**228**	**0.29**	**-1.25**	**0.10**	**< 0.01**	**↓**
**P69905**	**Hemoglobin subunit alpha**	**339**	**0.27**	**-1.30**	**0.07**	**< 0.01**	**↓**
**A0M8Q6**	**Immunoglobulin lambda constant 7**	**199**	**0.27**	**-1.32**	**0.12**	**< 0.01**	**↓**
**P01861**	**Immunoglobulin heavy constant gamma 4**	**224**	**0.25**	**-1.39**	**0.08**	**< 0.01**	**↓**
**P69891**	**Hemoglobin subunit gamma-1**	**514**	**0.05**	**-2.91**	**0.05**	**< 0.01**	**↓**
**P69892**	**Hemoglobin subunit gamma-2**	**514**	**0.05**	**-2.95**	**0.04**	**< 0.01**	**↓**
**P02100**	**Hemoglobin subunit epsilon**	**514**	**0.05**	**-2.97**	**0.04**	**< 0.01**	**↓**
**P68871**	**Hemoglobin subunit beta**	**1059**	**0.05**	**-2.97**	**0.02**	**< 0.01**	**↓**
**P02042**	**Hemoglobin subunit delta**	**2300**	**0.05**	**-3.09**	**0.03**	**< 0.01**	**↓**
Q15118	[Pyruvate dehydrogenase (acetyl-transferring)] kinase isozyme 1, mitochondrial	72	-	-	-	-	T1
P07108	Acyl-CoA-binding protein	297	-	-	-	-	T1
P01011	Alpha-1-antichymotrypsin	68	-	-	-	-	T1
A8K2U0	Alpha-2-macroglobulin-like protein 1	109	-	-	-	-	T1
P04920	Anion exchange protein 2	45	-	-	-	-	T1
P03973	Antileukoproteinase	1419	-	-	-	-	T1
Q9H115	Beta-soluble NSF attachment protein	84	-	-	-	-	T1
P27482	Calmodulin-like protein 3	417	-	-	-	-	T1
P23528	Cofilin-1	269	-	-	-	-	T1
P01024	Complement C3	45	-	-	-	-	T1
P54108	Cysteine-rich secretory protein 3	885	-	-	-	-	T1
Q02487	Desmocollin-2	38	-	-	-	-	T1
Q92616	eIF-2-alpha kinase activator GCN1	57	-	-	-	-	T1
Q9GZZ8	Extracellular glycoprotein lacritin	2060	-	-	-	-	T1
Q01469	Fatty acid-binding protein 5	557	-	-	-	-	T1
Q5W0V3	FHF complex subunit HOOK interacting protein 2A	159	-	-	-	-	T1
P04075	Fructose-bisphosphate aldolase A	182	-	-	-	-	T1
Q08380	Galectin-3-binding protein	92	-	-	-	-	T1
Q9UJ14	Glutathione hydrolase 7	123	-	-	-	-	T1
P09211	Glutathione S-transferase P	1006	-	-	-	-	T1
Q9Y6R7	IgGFc-binding protein	45	-	-	-	-	T1
A0A075B6P5	Immunoglobulin kappa variable 2-28	236	-	-	-	-	T1
A2NJV5	Immunoglobulin kappa variable 2-29	236	-	-	-	-	T1
P06310	Immunoglobulin kappa variable 2-30	236	-	-	-	-	T1
A0A087WW87	Immunoglobulin kappa variable 2-40	236	-	-	-	-	T1
A0A0A0MRZ7	Immunoglobulin kappa variable 2D-26	236	-	-	-	-	T1
P01615	Immunoglobulin kappa variable 2D-28	236	-	-	-	-	T1
A0A075B6S2	Immunoglobulin kappa variable 2D-29	236	-	-	-	-	T1
A0A075B6S6	Immunoglobulin kappa variable 2D-30	236	-	-	-	-	T1
P01614	Immunoglobulin kappa variable 2D-40	236	-	-	-	-	T1
P18510	Interleukin-1 receptor antagonist protein	113	-	-	-	-	T1

O95274	Ly6/PLAUR domain-containing protein 3	118	-	-	-	-	T1
Q96DR8	Mucin-like protein 1	606	-	-	-	-	T1
P24158	Myeloblastin	177	-	-	-	-	T1
Q15406	Nuclear receptor subfamily 6 group A member 1	48	-	-	-	-	T1
P62937	Peptidyl-prolyl cis-trans isomerase A	226	-	-	-	-	T1
Q9Y536	Peptidyl-prolyl cis-trans isomerase A-like 4A	54	-	-	-	-	T1
P00558	Phosphoglycerate kinase 1	83	-	-	-	-	T1
P07205	Phosphoglycerate kinase 2	83	-	-	-	-	T1
P07602	Prosaposin	116	-	-	-	-	T1
Q6P5S2	Protein LEG1 homolog	2046	-	-	-	-	T1
A8MUU1	Putative fatty acid-binding protein 5-like protein 3	79	-	-	-	-	T1
Q53EL9	Seizure protein 6 homolog	70	-	-	-	-	T1
P29508	Serpin B3	82	-	-	-	-	T1
P48594	Serpin B4	82	-	-	-	-	T1
Q9H299	SH3 domain-binding glutamic acid-rich-like protein 3	305	-	-	-	-	T1
P35326	Small proline-rich protein 2A	733	-	-	-	-	T1
P35325	Small proline-rich protein 2B	373	-	-	-	-	T1
P22532	Small proline-rich protein 2D	373	-	-	-	-	T1
P22531	Small proline-rich protein 2E	373	-	-	-	-	T1
Q96RM1	Small proline-rich protein 2F	75	-	-	-	-	T1
Q9BYE4	Small proline-rich protein 2G	347	-	-	-	-	T1
Q9UBC9	Small proline-rich protein 3	1968	-	-	-	-	T1
Q8WXA9	Splicing regulatory glutamine/lysine-rich protein 1	63	-	-	-	-	T1
P20061	Transcobalamin-1	201	-	-	-	-	T1
P60174	Triosephosphate isomerase	181	-	-	-	-	T1
Q8IXR9	Uncharacterized protein C12orf56	54	-	-	-	-	T1
P11684	Uteroglobin	3363	-	-	-	-	T1
Q14508	WAP four-disulfide core domain protein 2	2229	-	-	-	-	T1
Q8NHQ9	ATP-dependent RNA helicase DDX55	235	-	-	-	-	T2
O95196	Chondroitin sulfate proteoglycan 5	36	-	-	-	-	T2
P00338	L-lactate dehydrogenase A chain	246	-	-	-	-	T2
Q9HCD5	Nuclear receptor coactivator 5	46	-	-	-	-	T2
P20742	Pregnancy zone protein	66	-	-	-	-	T2
Q8N6L0	Protein KASH5	52	-	-	-	-	T2
Q9NTJ3	Structural maintenance of chromosomes protein 4	43	-	-	-	-	T2
P49848	Transcription initiation factor TFIID subunit 6	51	-	-	-	-	T2
P15515	Histatin-1	4751	4.57	1.52	0.53	0.85	SE
P09104	Gamma-enolase	16	1.48	0.39	0.39	0.83	SE
P10599	Thioredoxin	480	1.36	0.31	0.16	0.94	SE
P04406	Glyceraldehyde-3-phosphate dehydrogenase	29	1.27	0.24	0.27	0.83	SE
P0DOX6	Immunoglobulin mu heavy chain	148	1.15	0.14	0.16	0.77	SE
P01871	Immunoglobulin heavy constant mu	148	1.13	0.12	0.13	0.81	SE

P61626	Lysozyme C	484	1.05	0.05	0.07	0.66	SE
P01833	Polymeric immunoglobulin receptor	6275	1.02	0.02	0.04	0.55	SE
P01591	Immunoglobulin J chain	4092	1.01	0.01	0.06	0.59	SE
P04080	Cystatin-B	4062	0.99	-0.01	0.24	0.46	SE
P01834	Immunoglobulin kappa constant	2112	0.97	-0.03	0.06	0.21	SE
P0CG38	POTE ankyrin domain family member I	559	0.97	-0.03	0.24	0.68	SE
P01034	Cystatin-C	2999	0.95	-0.05	0.06	0.28	SE
P59666	Neutrophil defensin 3	887	0.95	-0.05	0.13	0.39	SE
P0DOX7	Immunoglobulin kappa light chain	317	0.91	-0.09	0.07	0.12	SE
P59665	Neutrophil defensin 1	887	0.91	-0.09	0.13	0.36	SE
P25311	Zinc-alpha-2-glycoprotein	80	0.87	-0.14	0.21	0.34	SE
Q8TAX7	Mucin-7	1302	0.85	-0.16	0.15	0.16	SE
P11142	Heat shock cognate 71 kDa protein	177	0.84	-0.18	0.36	0.34	SE
P13929	Beta-enolase	44	0.80	-0.22	0.25	0.14	SE
P29401	Transketolase	115	0.80	-0.22	0.29	0.21	SE
P11021	Endoplasmic reticulum chaperone BiP	169	0.79	-0.23	0.51	0.30	SE
Q01518	Adenylyl cyclase-associated protein 1	464	0.79	-0.23	0.30	0.31	SE
P48741	Putative heat shock 70 kDa protein 7	261	0.77	-0.26	0.42	0.25	SE
P54652	Heat shock-related 70 kDa protein 2	177	0.75	-0.29	0.45	0.28	SE
P17066	Heat shock 70 kDa protein 6	261	0.74	-0.30	0.46	0.27	SE
P00739	Haptoglobin-related protein	68	0.72	-0.33	0.44	0.31	SE
P01009	Alpha-1-antitrypsin	171	0.71	-0.34	0.19	0.07	SE
P06396	Gelsolin	57	0.70	-0.35	0.23	0.09	SE
P34931	Heat shock 70 kDa protein 1-like	232	0.70	-0.36	0.21	0.06	SE
P30613	Pyruvate kinase PKLR	45	0.63	-0.46	0.40	0.15	SE
P06744	Glucose-6-phosphate isomerase	137	0.59	-0.53	0.27	0.05	SE
P15516	Histatin-3	2572	0.15	-1.93	0.54	0.12	SE

Note: Ratio T1/T2 (fold change) = ratio between pregnancy (T1) and after delivery (T2) for NWP; Log(e) ("e" is a constant = 2.71); SD, standard deviation; *p*, statistical significance (adjusted by False Discovery Rate–FDR = 4); ↑ = up-regulated (1-p > 0.95); ↓ = down-regulated (p < 0.05); SE = similar expression compared to T2; bold lines refer to up- or down-regulated proteins by more than 2-fold
