# Supplementary figures and images for: Label-Free Quantitative Proteomic Analysis Reveals Inflammatory Pattern Associated with Obesity and Periodontitis in Pregnant Women
Source: Metabolites. 2022 Nov 10;12(11):1091. doi: 10.3390/metabo12111091 (PMC9692340; doi:10.3390/metabo12111091)

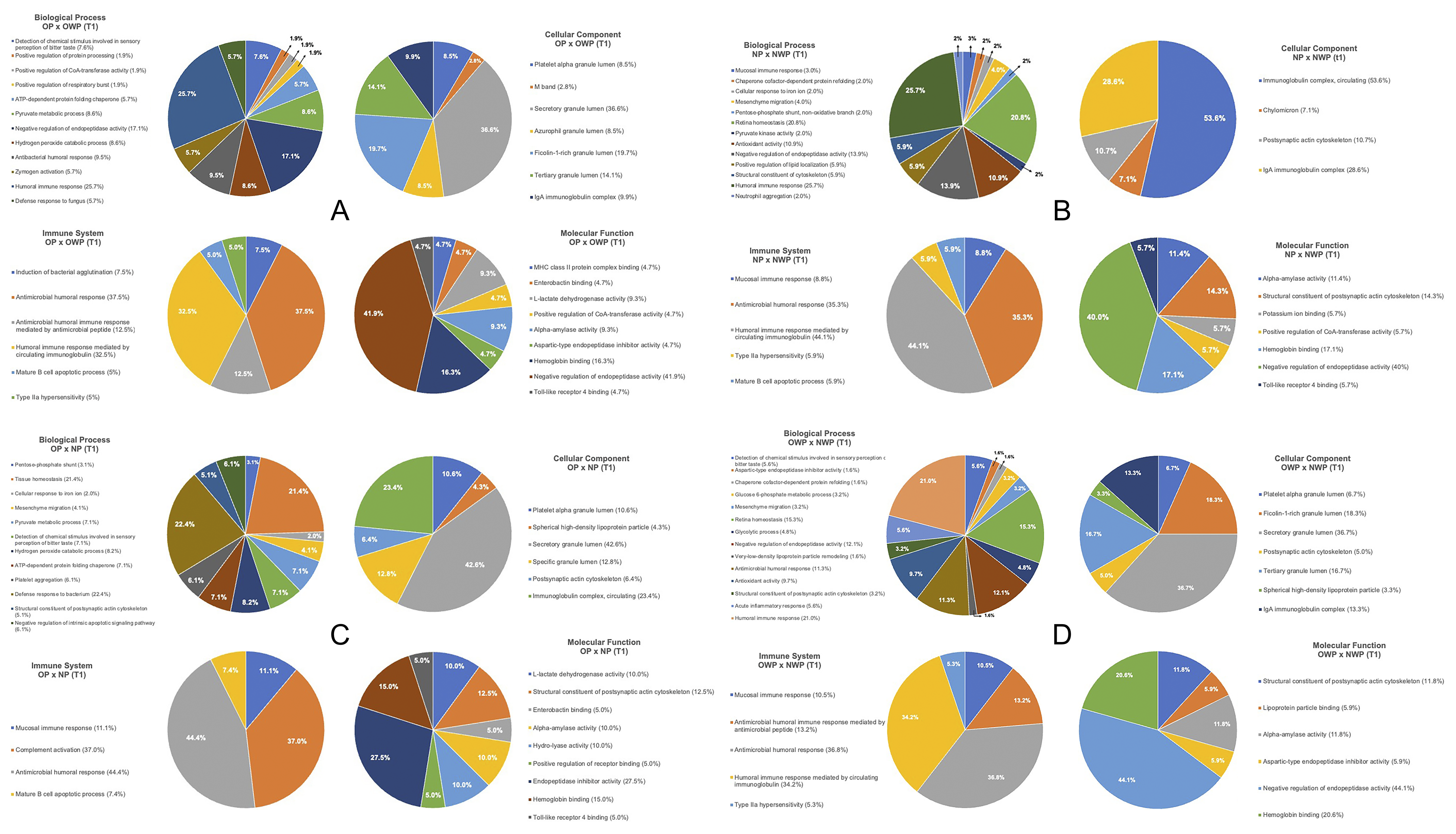

Supplement: Supplementary file 1 [file metabolites-12-01091-s001.zip › Supplementary file S4.tif]

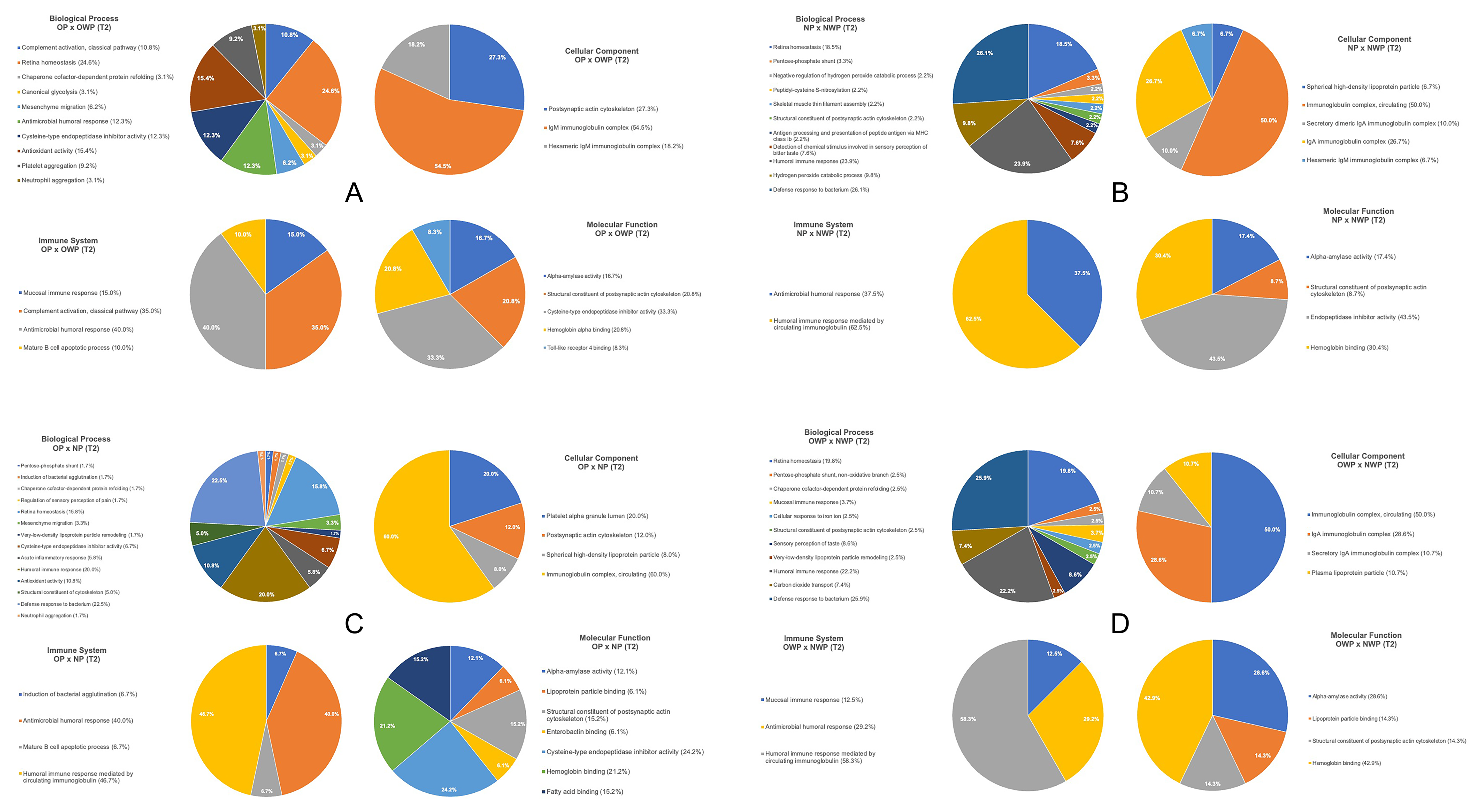

Supplement: Supplementary file 1 [file metabolites-12-01091-s001.zip › Supplementary file S5.tif]

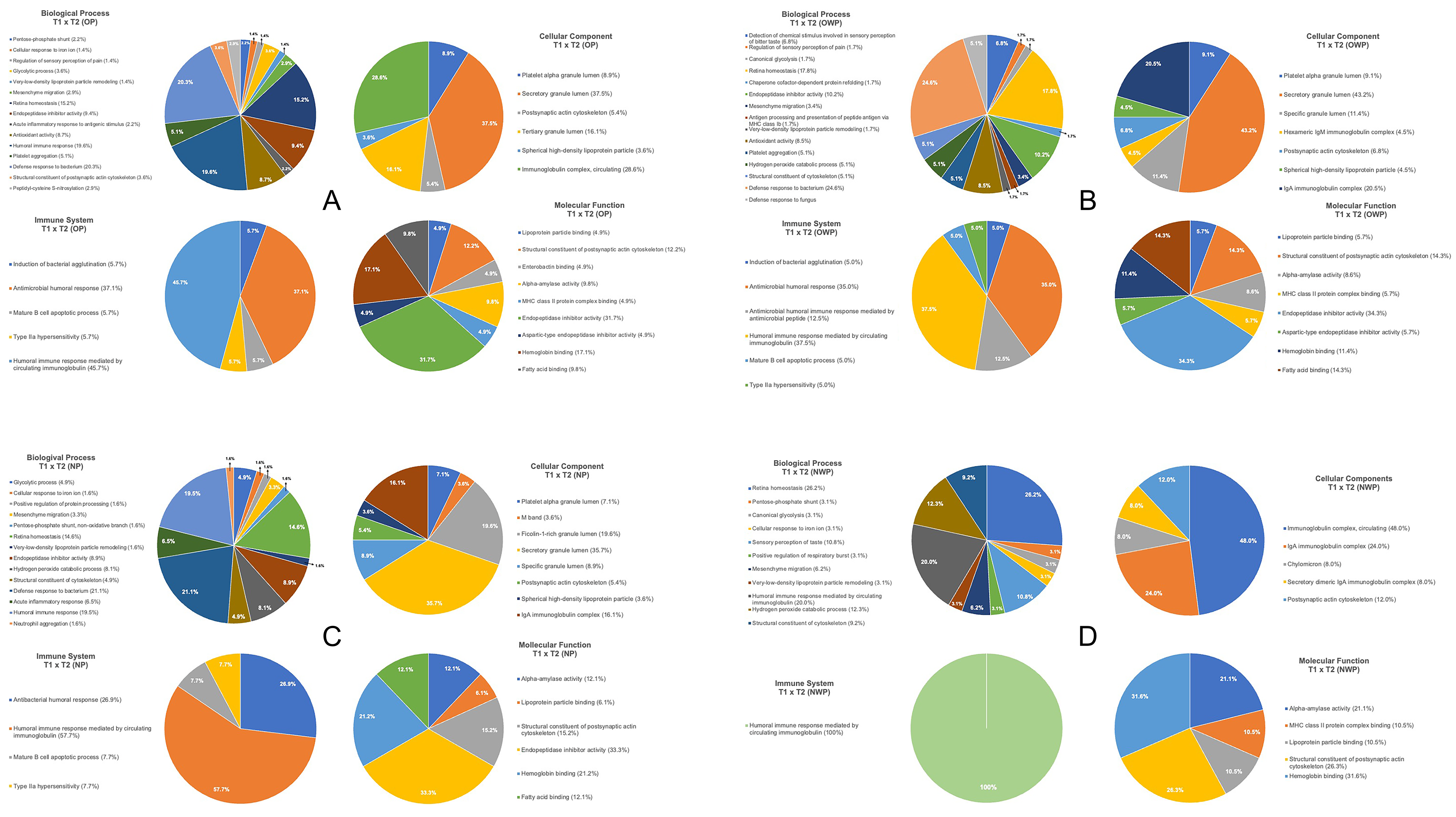

Supplement: Supplementary file 1 [file metabolites-12-01091-s001.zip › Supplementary file S6.tif]
